# Differential oxidation of protein-tyrosine phosphatases during zebrafish caudal fin regeneration

**DOI:** 10.1038/s41598-017-07109-8

**Published:** 2017-08-16

**Authors:** Wei Wu, Alexander James Hale, Simone Lemeer, Jeroen den Hertog

**Affiliations:** 1 Biomolecular Mass Spectrometry and Proteomics, Bijvoet Center for Biomolecular Research and Utrecht Institute for Pharmaceutical Sciences, Utrecht University and Netherlands Proteomics Centre, Utrecht, The Netherlands; 2 Hubrecht Institute – KNAW and University Medical Center Utrecht, Utrecht, The Netherlands; 3 Institute Biology Leiden, Leiden University, Leiden, The Netherlands

## Abstract

Zebrafish have the capacity to regenerate lost tissues and organs. Amputation of the caudal fin results in a rapid, transient increase in H_2_O_2_ levels emanating from the wound margin, which is essential for regeneration, because quenching of reactive oxygen species blocks regeneration. Protein-tyrosine phosphatases (PTPs) have a central role in cell signalling and are susceptible to oxidation, which results in transient inactivation of their catalytic activity. We hypothesized that PTPs may become oxidized in response to amputation of the caudal fin. Using the oxidized PTP-specific (ox-PTP) antibody and liquid chromatography-mass spectrometry, we identified 33 PTPs in adult zebrafish fin clips of the total of 44 PTPs that can theoretically be detected based on sequence conservation. Of these 33 PTPs, 8 were significantly more oxidized 40 min after caudal fin amputation. Surprisingly, Shp2, one of the PTPs that were oxidized in response to caudal fin amputation, was required for caudal fin regeneration. In contrast, Rptpα, which was not oxidized upon amputation, was dispensable for caudal fin regeneration. Our results demonstrate that PTPs are differentially oxidized in response to caudal fin amputation and that there is a differential requirement for PTPs in regeneration.

## Introduction

Epimorphic regeneration is the perfect replacement of lost tissues, organs, or limbs. Zebrafish can fully regenerate multiple organs after injury, including the heart, retina, spinal cord, and caudal fin^1, 2^. Many genes have been implicated and multiple signalling pathways have been validated to be essential for regeneration to proceed, including fibroblast growth factor (FGF), sonic hedgehog, bone morphogenetic protein, Wnt, and Notch^3, 4^. However, it remains unclear how this complex process is initiated. One of the first responses following amputation of the zebrafish caudal fin is a burst of hydrogen peroxide (H_2_O_2_) that emanates from the wound site and extends into the tissue^5^. Inhibition of this burst of H_2_O_2_ impairs caudal fin regeneration^6^, demonstrating it is essential for this process.

H_2_O_2_ is a reactive oxygen species (ROS) that, as well as a chemoattractant, acts as a second messenger molecule to regulate intracellular signalling including mitogen activated protein kinase (MAPK), phosphoinositide-3-kinase (PI3K)/AKT, and NF-κB signal transduction^7, 8^. H_2_O_2_ reversibly oxidizes cysteine residues that are in the thiolate anion form due to a low pKa, which results from their microenvironment. Often, the active-site cysteine of enzymes has a low pKa, which actually confers catalytic activity. Reversible oxidation of these cysteine residues may temporarily activate or inactivate these enzymes^9^.

Protein-tyrosine phosphatases (PTPs) constitute a family of active-site cysteine enzymes that mediate tyrosine dephosphorylation^10–12^. A total of 125 genes encoding enzymes with PTP activity have been identified in the human genome, 116 of which are cysteine-based, and constitute an enzyme superfamily divided into classical PTPs, dual-specificity PTPs, and low molecular weight PTPs^13, 14^. The classical PTPs are further subdivided into receptor and non-receptor PTPs. Classical PTPs are defined by having at least one catalytic domain with a conserved signature motif (I/V)H**C**SAGXXR(S/T)G, containing the catalytic cysteine (in bold), which is essential for catalysing the removal of the phosphate group from phospho-tyrosine residues. The sulphur atom in the catalytic cysteine of active PTPs is in the thiolate anion form (S^−^). H_2_O_2_ inactivates PTPs by reversibly oxidizing this sulphur atom to sulphenic acid (SOH), a labile state that rapidly rearranges to form a sulphenylamide with an adjacent nitrogen^15^ or a disulphide bond with a nearby cysteine^16^. These states help protect the catalytic cysteine from further irreversible hyperoxidation to sulphinic acid (SO_2_H) or sulphonic acid (SO_3_H) states^17^. A monoclonal antibody (ox-PTP Ab) was raised against a hexapeptide encoding the conserved PTP signature motif with a triply oxidized cysteine, VHC_SO3H_SAG, which can be used to detect reversible and irreversible oxidation of PTPs^18, 19^. The susceptibility to oxidation differs from PTP to PTP^10, 20^, suggesting dose-dependent specificity in ROS signalling. Mechanistically, oxidation-mediated inhibition of PTPs results in the selective amplification or attenuation of specific signalling pathways, such as FGF, PI3K/AKT, and MAPK, ultimately regulating fundamental cellular processes, including proliferation, differentiation, and cell-cell adhesion^21^.

Considering the high susceptibility of PTPs to oxidation, we hypothesized that the H_2_O_2_ burst following caudal fin amputation may oxidize PTPs. Using the ox-PTP-specific antibody and mass spectrometry, we detected 37 out of 52 predicted zebrafish PTP motif peptides, 8 of which were oxidized following caudal fin amputation. We next functionally assessed the role of PTPs during regeneration. Shp2, a cytoplasmic PTP with a central role in signalling was one of the 8 oxidized PTPs. Interestingly, zebrafish mutant embryos lacking functional Shp2 displayed impaired regeneration. Zebrafish embryos lacking Rptpα, which was not oxidized upon caudal fin amputation, regenerated their caudal fin normally. These results reveal differential oxidation of PTPs following caudal fin amputation *in vivo*, and suggest a differential requirement for PTPs in zebrafish caudal fin regeneration.

## Results

### Detecting *in vivo* PTP oxidation following zebrafish caudal fin amputation

**Figure 1:**
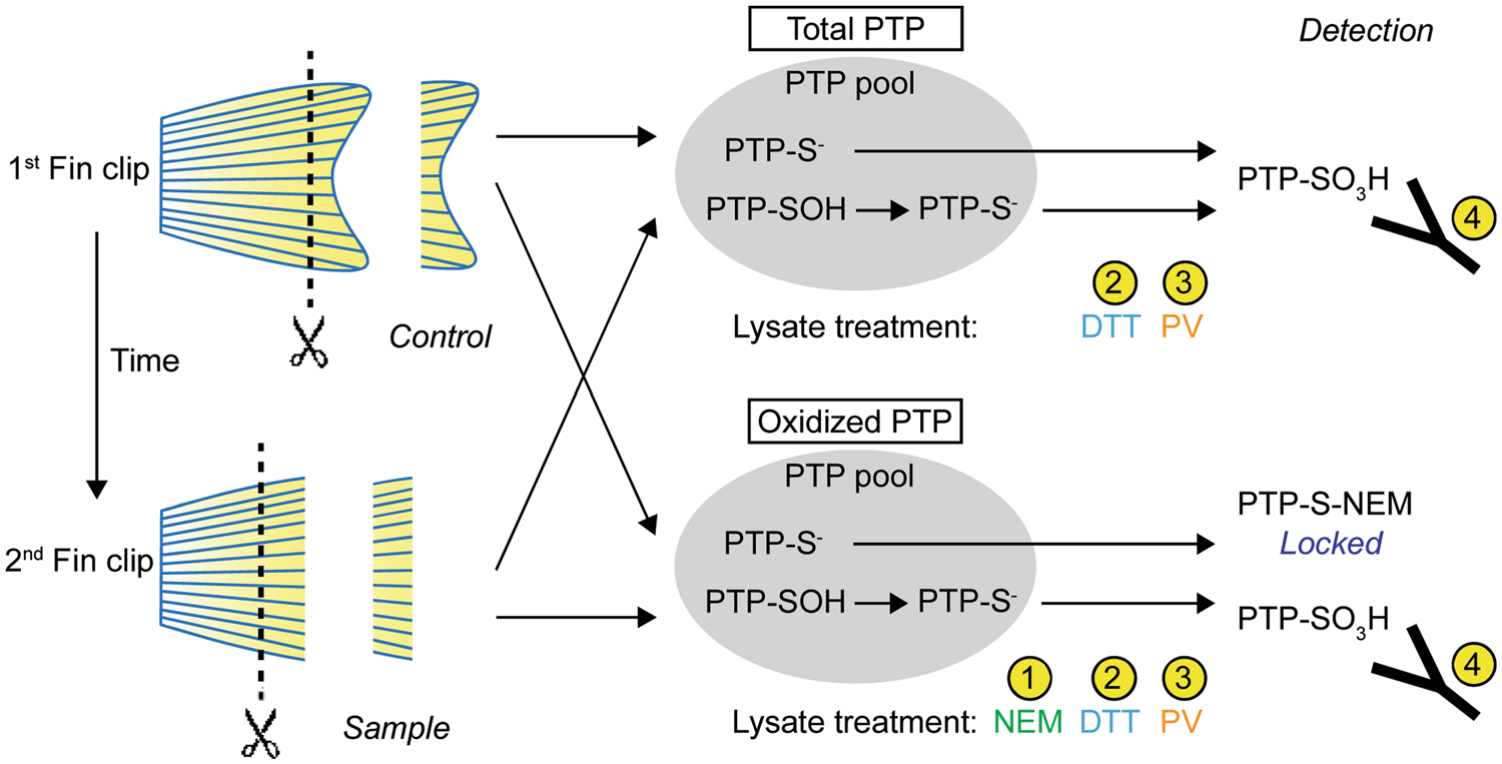
Detection of total and oxidized PTPs in zebrafish caudal fins. Zebrafish caudal fin tissue was obtained by sequential amputation. The first fin clips (control) were snap-frozen immediately. The second fin clips (sample) were taken after 40 min to allow for H_2_O_2_ to form and oxidize proteins, and were snap-frozen immediately as well. The PTP pool in cells consists of reduced (PTP-S^−^) and reversibly oxidized (PTP-SOH) PTPs. Caudal fin clips were either lysed in reducing (DTT-containing) or alkylating (NEM-containing) lysis buffer to detect total and oxidized PTPs, respectively, for both control and sample caudal fin clips. As the ox-PTP antibody only binds hyperoxidized PTPs (PTP-SO_3_H), a sequential reduction and oxidation process is required for detection by immunoprecipitation. 1. NEM in the lysis buffer protects reduced PTPs from hyperoxidation. 2. DTT reduces all oxidized PTPs to allow hyperoxidation. 3. pervanadate (PV) hyperoxidizes all reduced PTPs to an irreversibly oxidized state (PTP-SO_3_H). 4. PTP-SO_3_H PTPs are recognized by the ox-PTP-specific antibody. Immunoprecipitation allows detection by LC-MS/MS. Lysis in the absence of NEM results in detection of total PTPs. Comparison of the NEM treated first and second fin clips allows detection of the change in oxidized PTPs in response to caudal fin amputation.

To investigate PTP oxidation following amputation, we isolated zebrafish caudal fin tissue by performing two sequential amputations with a 40 min interval to allow for the H_2_O_2_ gradient to form, emanating from the wound margin^5^ (Fig. 1). Repeated amputation does not affect the regenerative capacity of zebrafish^22^. The first (control) and second (sample) caudal fin clips were snap-frozen immediately following amputation and lysed in reducing or alkylating lysis buffer to detect all PTPs or oxidized PTPs, respectively (Fig. 1).

Under normal conditions, cells contain reduced PTPs (PTP-S^−^) and reversibly oxidized PTPs (PTP-SOH)^11^. Reduction of all PTPs in the lysate by treatment with dithiotreitol (DTT), followed by hyperoxidation using pervanadate (PV), converts all catalytic cysteines into the sulphonic acid (SO_3_H) state, allowing detection of all PTPs with the ox-PTP-specific antibody. Reversibly oxidized PTPs can be detected by first lysing in the presence of N-ethylmaleimide (NEM), which alkylates all reduced cysteines, followed by reduction of the oxidized catalytic cysteines using DTT, and hyperoxidation using pervanadate (PV). As the reduced catalytic cysteines are protected from detection by NEM, only the catalytic cysteines that were oxidized at the time of lysis will be detected using the ox-PTP-specific antibody. Comparison of oxidized PTPs with all PTPs in a sample facilitates assessment of the extent of oxidation of the PTP family. Here, we employed protocols that we and others developed^10, 18–20^ to detect *in vivo* PTP oxidation following amputation of the zebrafish caudal fin using liquid chromatography mass spectrometry (LC-MS)^23^ (Fig. 1).

### Identification of oxidized PTPs following zebrafish caudal fin amputation

We searched the zebrafish Uniprot + trEMBL database^24^ for entries containing the [I/V]HCS[A/V]GXGR[S/T]G motif, and found 52 distinct PTP motif peptides containing this signature motif, indicating that those may serve as the epitope for the ox-PTP antibody (Fig. S1). Given the tandem PTP domains in some receptor PTPs, the 52 PTP motif peptides represent 44 distinct PTP proteins. It is noteworthy that the zebrafish genome was duplicated early in evolution. Whilst some of the duplicated chromosomes were lost, the duplicated genes that remain have complementary or diverging expression patterns and exhibit redundant or complementary functions^25, 26^. Among the zebrafish classical PTPs, 5 non-receptor PTPs and 9 receptor PTPs are duplicated, the “a” and “b” entries (Fig. S1). It is noteworthy that the annotation of proteins in the Uniprot + trEMBL database is incomplete and the majority of sequences submitted for zebrafish PTPs have not been reviewed or validated. The A0A1L1QZV1 and F1QWY5_CA16B entries (Fig. S1) actually represent Ptprr and Ptprgb, respectively, based on sequence comparison with human PTPs.

**Figure 2:**
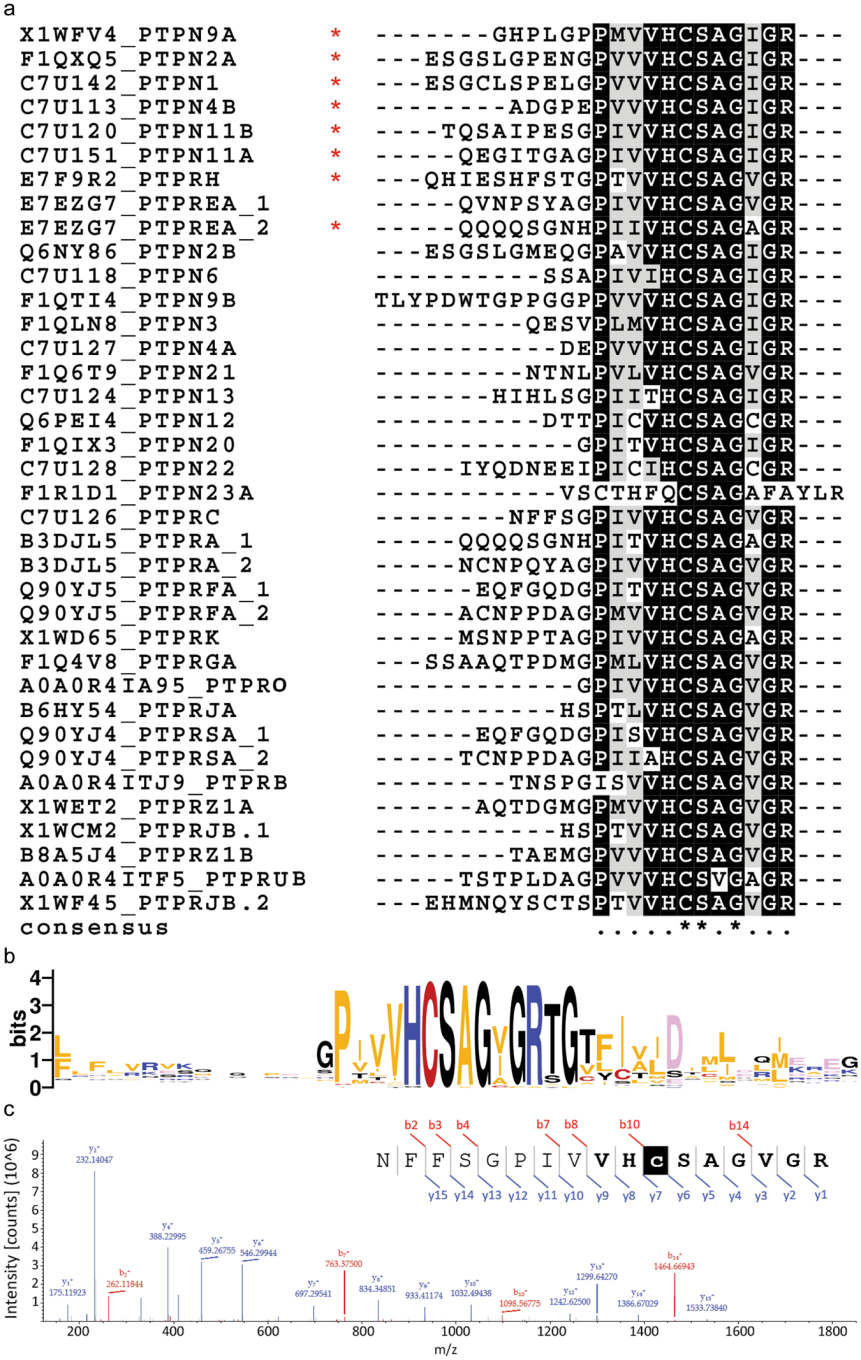
Detection of PTP motif peptides using LC-MS/MS. Caudal fin lysates were isolated and processed as described in Fig. 1 and LC-MS/MS was used to identify PTP motif peptides. **(a)** Clustal Omega alignment of 37 PTP motif peptides that were detected. Intensity of shading represents degree of sequence conservation. Oxidation of 8 out of 37 PTP motif peptides (*) was detected in the second fin clips 40 min after the first fin clips. **(b)** Sequence logo obtained from 37 PTP catalytic sites and 20 flanking residues. Overall height of stack at each position indicates sequence conservation; height of symbols within the stack indicates relative frequency of each amino acid in the position. Low conservation in sequences preceding the catalytic site allows unambiguous identification of PTP species. **(c)** Representative MS/MS spectrum of a NFFSGPIVVHcSAGVGR peptide from PTPRC (CD45). Inset: peptide sequence, annotated with matched y-ions (blue) and b-ions (red). Consensus PTP motif in bold; catalytic cysteine residue in white.

In order to unambiguously identify which PTPs are oxidized, first fin clips (control) and second fin clips (40 min) were isolated and processed as outlined in Fig. 1. Fin clips were isolated from 10 adult zebrafish per condition and the lysates were pooled to average out biological differences between individual fish. The experiment was performed in triplicate. Following PV treatment, fin lysates were digested with trypsin and ox-PTP peptides were immunoprecipitated using the ox-PTP-specific antibody. Immunoprecipitated peptides were subsequently analysed by LC-MS/MS. Since trypsin cleaves after the amino acids lysine and arginine, all PTP motif peptide sequences detected end with an arginine residue within the catalytic domain ([I/V]HCS[A/V]GXG**R**). Unambiguous identification of PTPs was thus achieved using the sequence upstream of the consensus sequence (VHCSA/VG) of every unique PTP motif peptide, where low sequence conservation is observed. Using this approach, we identified 37 PTP motif peptides containing an active-site catalytic cysteine in zebrafish caudal fin lysates (Fig. 2a,b, Table S1). These 37 PTP motif peptides are derived from 33 distinct PTP proteins, indicating that 75% (33 of 44) of all PTPs were expressed and detected in the zebrafish caudal fin. The immunoprecipitation with the ox-PTP antibody resulted in great enrichment of PTP motif peptides. For comparison, we analysed fin lysate by LC-MS/MS and detected 2698 proteins, amongst which only 2 were PTPs. In the ox-PTP immunoprecipitates, we identified peptides derived from 124 proteins in total, 33 of which represent distinct PTPs, demonstrating the enrichment achieved by ox-PTP immunoprecipitation.

A representative spectrum obtained for the NFFSGPIVVHcSAGVGR peptide from Ptprc (CD45) is shown in Fig. 2c, where the peptide was almost fully sequenced with near complete y- and b-ion ladder. Representative spectra for all zebrafish PTP catalytic site peptides detected are presented in Fig. S2. The high quality of our data facilitated the observation of diagnostic ions in the raw MS/MS spectra and allowed us to distinguish between highly homologous PTP peptides, such as Ptpn1 and Ptpn2a (2 residue difference), or Rptpα_2 and Rptpεb_2 (single residue difference) (Fig. S2, p1, 6, 21 and 30). Several receptor-type PTPs contain two catalytic site cysteine motifs, representing the two PTP domains, and both PTP motif peptides were detected in one experiment (Table S1), albeit with different MS signal intensities. This is presumably due to the primary peptide sequences having different precursor ionization and MS fragmentation efficiencies.

**Figure 3:**
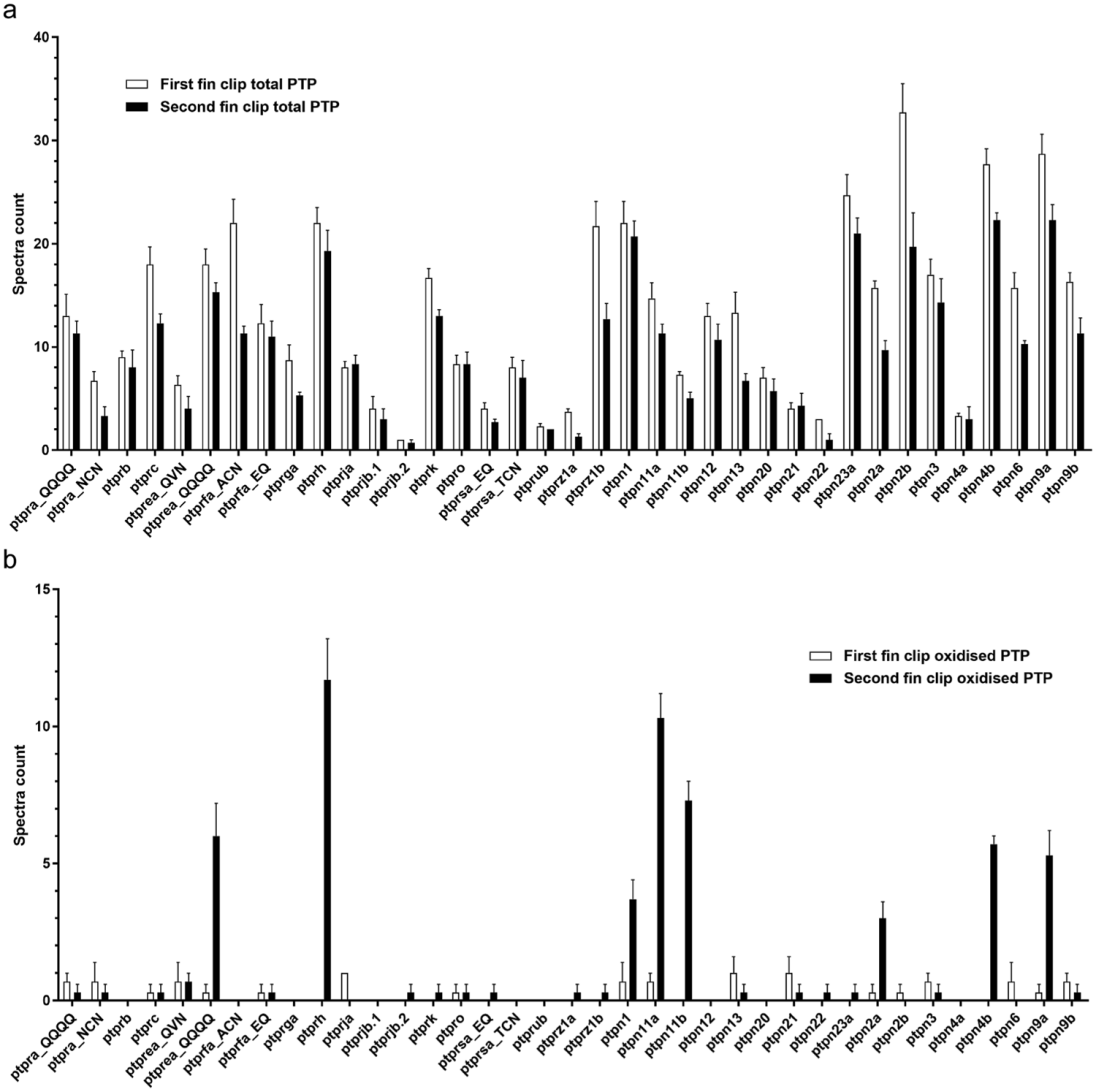
Differential PTP oxidation following caudal fin amputation. (**a**) Total detectable PTP repertoire in zebrafish caudal fin clips. Spectral counts ± SE do not differ significantly between first and second fin clips (paired *t*-test, *p* > 0.05; *n* = 3). **(b)** Oxidized PTPs detected from first and second fin clips. PTPREA_QQQQ, PTPRH, PTPN1, PTPN11a, PTPN11b, PTPN2a, PTPN4b and PTPN9a are significantly more oxidized in the second fin clip than in the first fin clip (paired *t*-test, *p* < 0.05; *n* = 3).

Differences in abundance of the PTPs were assessed by comparing the spectral counts from the samples lysed in a reducing buffer of both the first and second fin clips (Fig. 1, Total PTP) (Table S2). These results show large differences in abundance of distinct PTPs in zebrafish caudal fins (Fig. 3a). However, for the same PTP, only small differences in abundance were observed between the first and second fin clip (Fig. 3a).

The abundance of oxidized PTPs was assessed by analysis of samples lysed in an alkylating buffer of the first and second fin clip (Fig. 1, Oxidized PTP). A difference in spectral counts between the first and second fin clip represents a change in PTP oxidation upon amputation of the caudal fin. Out of 37 PTP motif peptides, 8 were found significantly more oxidized in the second fin clip than in the first fin clip (*p* < 0.05; Fig. 3b). We conclude that *in vivo* oxidation of those 8 PTPs (asterisks in Fig. 2a) was enhanced in response to caudal fin amputation. As an additional control, the first and second fin clip were lysed in reducing buffer to reduce all reversibly oxidized PTPs, then treated with NEM to block all reduced cysteines and subsequently processed to detect oxidized PTPs. This control resulted in detection of background levels of PTP motif peptides in the first and second fin clips (Table S2), indicating that there are no triply oxidized PTPs in these lysates before processing. Moreover, these results indicate that the 8 PTPs that were detected to be oxidized in response to amputation of the caudal fin, were all reversibly oxidized, because DTT-mediated reduction of the lysates of the second fin clip prior to processing for detection of oxidized PTPs reduced the signal to background levels. These results demonstrate that our approach effectively detected individual PTP motif peptides in zebrafish caudal fins as well as changes in their oxidation state following amputation.

### Differential requirement for PTP function in zebrafish caudal fin regeneration

Our results reveal that many PTPs are detected to be expressed in the caudal fin, but only 8 are abundantly oxidized following caudal fin amputation. We next investigated whether PTPs have a functional role in zebrafish caudal fin regeneration. To this end, we performed caudal fin regeneration assays on zebrafish embryos lacking functional Shp2 (*ptpn11a* ^−/−^ *ptpn11b* ^−/−^) or Rptpα (*ptpra* ^−/−^). It is noteworthy that Shp2a and Shp2b were both abundantly oxidized following caudal fin amputation, whereas Rptpα was not oxidized under these conditions (Figs 2a and 3).

**Figure 4:**
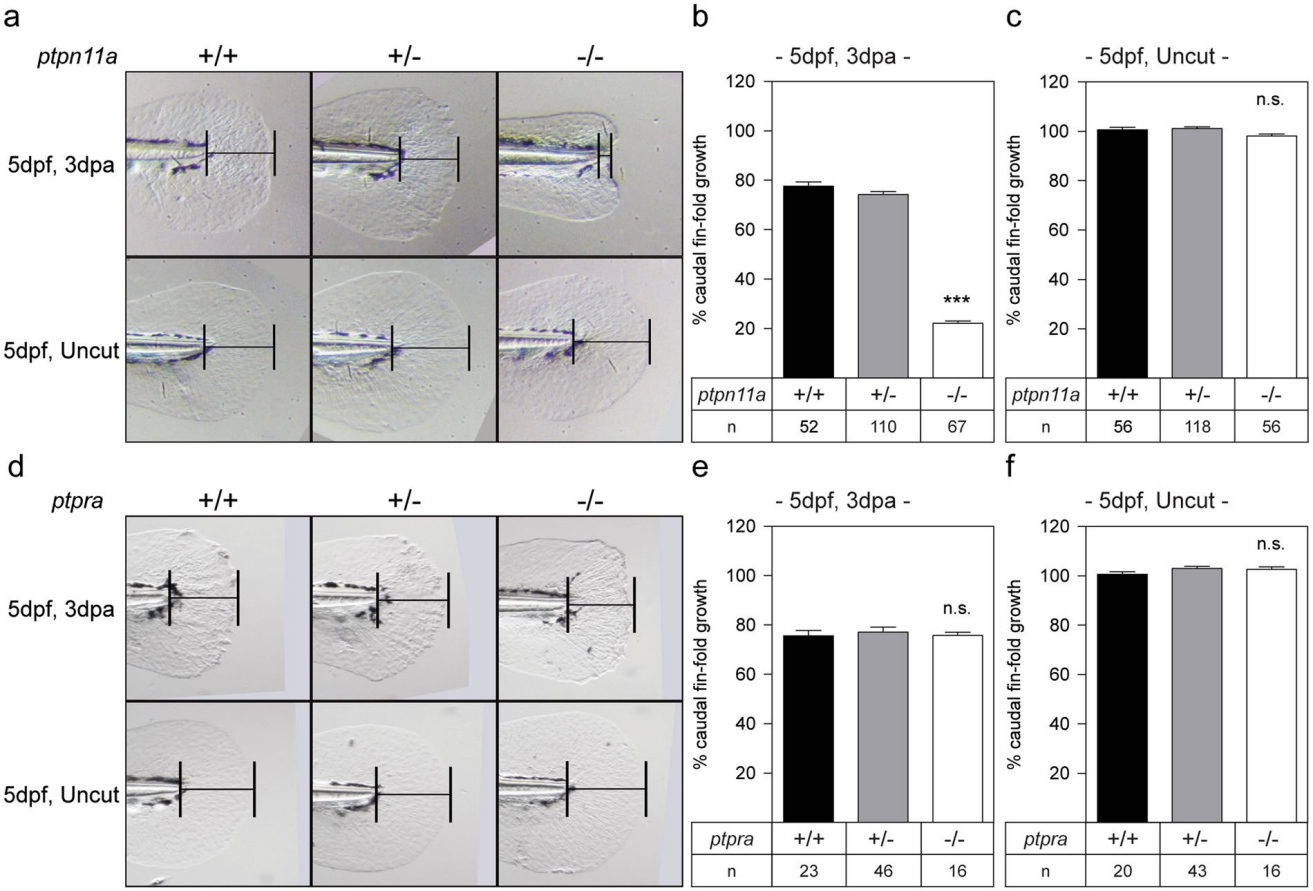
Impaired caudal fin regeneration in Shp2 deficient, but not Rptpα deficient embryos. Zebrafish embryos from a *ptpn11a* ^+/−^ *ptpn11b* ^−/−^ in-cross and a *ptpra* ^+/−^ in-cross were amputated at 2 dpf and caudal fin regeneration was assessed at 3 dpa (i.e. 5dpf, 3 dpa). Equivalent uncut embryos were monitored as controls (i.e. 5dpf, uncut). **(a, d)** Representative images of embryo caudal fins are shown. **(b,c)** Means of caudal fin growth of embryos lacking Shp2 (*ptpn11a* ^−/−^ *ptpn11b* ^−/−^) embryos following amputation and uncut controls are depicted relative to caudal fin growth of uncut *ptpn11a* ^+/+^ *ptpn11b* ^−/−^ embryos. **(e**,**f)** Means of caudal fin growth of embryos lacking Rptpα (*ptpra* ^−/−^) following amputation and uncut controls are depicted relative to caudal fin growth of uncut *ptpra* ^+/+^ embryos. Regeneration was quantified by measuring the distance from the tip of the notochord to the edge of the caudal fin as indicated. Means of amputated *ptpn11* ^−/−^ *ptpn11b* ^−/−^ or *ptpra* ^−/−^ embryos were compared to amputated *ptpn11a* ^+/+^ *ptpn11b* ^−/−^ or *ptpra* ^+/+^ embryos, respectively, using a Mann-Whitney U-test with a confidence level of 99%. Significance: ***p < 0.001; n.s. not significant; error bars indicate standard error of the mean. All embryos were genotyped.

The *ptpn11a* ^−/−^ *ptpn11b* ^−/−^ zebrafish have been previously validated and characterized^27^. Zebrafish heterozygous for Rptpα (*ptpra* ^+/−^) were obtained from the European Zebrafish Resource Centre (EZRC) and the (*ptpra* ^−/−^) offspring did not express Rptpα protein at 4 days post fertilisation (dpf) (Fig. S3), indicating that these are bona fide knock-outs. Both *ptpn11a* ^−/−^ *ptpn11b* ^−/−^ and *ptpra* ^−/−^ embryos are embryonically lethal at 6–8 dpf^27, 28^. Because homozygous adults were not viable, we investigated caudal fin regeneration during embryogenesis between 2 dpf and 5 dpf. It is noteworthy that the mechanism of caudal fin regeneration is highly similar in zebrafish embryos and adults^29^. Normally, amputated caudal fins of 2 dpf embryos almost completely regenerate in the course of 3 days (5dpf, 3 dpa). Surprisingly, *ptpn11a* ^−/−^ *ptpn11b* ^−/−^ zebrafish embryos showed severely impaired regeneration of their caudal fins (Fig. 4a,b). The normal developmental growth rate of the caudal fins was unaffected in *ptpn11a* ^−/−^ *ptpn11b* ^−/−^ embryos (Fig. 4a,c). In contrast, *ptpra* ^−/−^ zebrafish embryos regenerated their caudal fins to the same extent as their siblings and no significant differences were observed in fin growth between *ptpra* ^−/−^ embryos and siblings (Fig. 4d–f). These results show differential requirement for PTPs to facilitate caudal fin regeneration in zebrafish embryos.

## Discussion

Our results demonstrate successful detection and identification of PTPs in zebrafish caudal fin tissue, and reversible oxidation of a subset of PTPs following caudal fin amputation (Fig. 3, Table S2). PTP motif peptides were efficiently immunoprecipitated and identified by LC-MS/MS. According to the Uniprot + trEMBL database, these peptides belong to 33 distinct PTPs, including 17 non-receptor and 16 receptor type PTPs, covering 75% of all theoretically observable PTPs (*cf*. Figs 2a and S1). This is to our knowledge the largest repertoire of zebrafish PTPs identified in a single proteomics experiment, and serves to confirm the existence of numerous PTP species that were previously annotated only at the transcript level. As not all predicted PTP peptides may be expressed in the zebrafish caudal fin, the local coverage may be even greater.

Of the 33 PTPs that were detected in zebrafish caudal fin clips, 8 were oxidized 40 minutes after amputation, including 6 non-receptor and 2 receptor type PTPs (Figs 2a and 3b): Ptpn1, Ptpn2a, Ptpn4b, Ptpn9a, Ptpn11a, Ptpn11b, Rptph, and Rptpεa. Abundance of the 8 oxidized PTP peptides and the remaining 29 PTP peptides that were identified was not significantly different between the first and second fin clips, based on semi-quantitative spectral counting (Fig. 3a and Table S2), indicating that the increase in spectral counts of these 8 peptides was caused by enhanced oxidation. Oxidation of these 8 PTPs was also not related to baseline PTP expression level (*cf*. Fig. 3a,b). In general, this indicates that not all PTPs are equally susceptible to oxidation and that PTPs are oxidized differentially, consistent with our earlier work *in vitro* ^10^. To our knowledge, our results demonstrate for the first time that differential PTP oxidation occurs in response to endogenous ROS production *in vivo*.

Interestingly, only the membrane-distal PTP domain (D2, QQQQSGNHPIIVHcSAGAGR) of Ptprεa becomes oxidized following caudal fin amputation (Figs 2a and 3b). This suggests that D2 is more sensitive to oxidation. We previously reported differential oxidation of the two catalytic domains of a close relative of Ptprεa, RPTPα. In *in vitro* experiments, the D2 domain of RPTPα is more sensitive to oxidation and appears to act as a redox sensor, regulating catalytic activity of RPTPα in an allosteric manner^19, 30^. Our current results in zebrafish caudal fins suggest that Ptprεa-D2 may function in a similar manner as the redox sensor for Ptprεa.

Little is known about the role of PTPs in regeneration. The PTP superfamily member, Phosphatase and Tensin Homologue (PTEN), a lipid phosphatase, is an essential regulator of PI3K/AKT signalling and has been implicated in limiting central nervous system axon regeneration^31^. PTP type IVA 1 belongs to the phosphatase of regenerating liver (PRL)-subfamily of PTPs and was originally identified as PRL-1 because it was upregulated following liver injury and it is required for proper cell cycle progression during liver regeneration^32, 33^. To date, the function of classical PTPs in regeneration had not been addressed. We performed regeneration assays on zebrafish embryos lacking either Shp2 or Rptpα. Surprisingly, embryos lacking Shp2 (*ptpn11a* ^−/−^ *ptpn11b* ^−/−^) did not regenerate (Fig. 4). Yet, embryos lacking Rptpα (*ptpra* ^−/−^) regenerated normally. These results indicate that Shp2, but not Rptpα, has a specific and essential role in zebrafish caudal fin regeneration. The requirement for functional Shp2 in regeneration on the one hand and oxidation-mediated inactivation of Shp2 upon amputation on the other, results in an apparent conundrum: functional Shp2 is required for regeneration and, at the same time, Shp2 is oxidized and thus inactivated in response to caudal fin amputation. Presumably, oxidation of Shp2 is transient. Following a lag-time, Shp2 becomes active again and exerts its essential function. However, we cannot exclude that oxidized, inactive, Shp2 has another, phosphatase-independent function in regeneration.

The function of oxidation of the 8 PTPs that are oxidized in response to zebrafish caudal fin regeneration remains to be determined. In general, whereas it is evident that PTPs become oxidized *in vitro*, analysis of the function of PTP oxidation *in vivo* is only recently emerging. For instance, oxidation of PTP1B and TC-PTP has a role in the progression of type 2 diabetes mellitus^34^. PTP1B is oxidized following platelet derived growth factor stimulation^35, 36^, resulting in an enhanced mitogenic response, and is also transiently oxidized in response to insulin, promoting insulin receptor and leptin signalling^37^. Oxidation of TC-PTP has been reported in response to hepatic oxidative stress, an early event in type 2 diabetes progression, and exacerbates obesity and insulin resistance^38^. Both PTP1B and TC-PTP have various roles in tumour biology, metabolism, inflammation and autoimmunity, reviewed in refs 39–41, and their oxidation is likely important in many other processes. In immune cells, activation of the T-cell receptor results in transient ROS-induced inactivation of SHP2, activating integrin signalling and promoting T cell adhesion^42^. The cytoplasmic isoform of PTPε, cyt-PTPε^43^, has an important role in maintaining osteoclast function^44^, and translocates to the nucleus following oxidative stress^45^. However, the function of this translocation remains to be determined. To date, nothing is known about oxidation of Ptpn4, Ptpn9, or Rptph.

In conclusion, we used a highly sensitive and robust approach to detect and identify PTPs in zebrafish caudal fin tissue. This method facilitated the identification of the oxidation of 8 different zebrafish PTPs following caudal fin amputation. Moreover, we provide evidence that Shp2, one of the oxidized PTPs, is essential for caudal fin regeneration. Considering the importance of ROS signalling in various physiological processes and the susceptibility of PTPs to ROS-mediated inactivation, screening for PTP oxidation in various conditions, including wound healing, cancer, diabetes, and ageing, might identify a general role for the inactivation of PTPs in these physiological processes.

## Methods

### Zebrafish husbandry

All procedures involving experimental animals were approved by the local animal experiments committee (Koninklijke Nederlandse Akademie van Wetenschappen-Dierexperimenten commissie) and performed according to local guidelines and policies in compliance with national and European law. The *ptpra* ^+/−^ zebrafish line was purchased from EZRC (allele *ptptra* ^*sa14038*^, item #15584). The *ptpn11a* ^+/−^ *ptpn11b* ^−/−^ zebrafish line in the Tuebingen Long fin (TL) background was previously created by target-selected gene inactivation (TSGI), and both *ptpn11a* ^*hu3459*^ and *ptpn11b* ^*hu5920*^ alleles result from non-sense mutations that lead to a premature stop codon upstream of the catalytic domain^27^. Adult *ptpra* ^+/−^ or *ptpn11a* ^+/−^ *ptpn11b* ^−/−^ zebrafish were in-crossed to generate *ptpra* ^−/−^ or *ptpn11a* ^−/−^ *ptpn11b* ^−/−^ embryos, respectively, for all experiments. Zebrafish were raised and maintained as described by Westerfield^46^ under a 14 hours light/ 10 hours dark cycle at 28.5 °C.

### Caudal fin amputation

Adult wild-type TL zebrafish of at least 3 months old were anaesthetized in 0.6 mM Tricaine and amputated with a stainless steel surgical blade (Swann-Morton no. 24). Amputated zebrafish were allowed to recover from anaesthesia in separate tanks (one individual per tank) at 28.5 °C. The second amputation was performed, as the first, after 40 mins or 8 h of recovery on the same fin area. Fin clips were placed in individual 1.5 ml tubes and snap-frozen in liquid nitrogen immediately following amputation. Zebrafish embryos were amputated as previously described^47^. Whole zebrafish embryos were lysed for genotyping at 72 hpa (5 dpf).

### Protein extraction, alkylation and hyperoxidation

Snap-frozen fin clips were sonicated in lysis buffer (50 mM HEPES, 150 mM NaCl, 10% glycerol, 1% NP-40, 1x protease inhibitor (Roche), 1x phosphatase inhibitor (Roche)), supplemented with either DTT (Biorad) or NEM (Sigma-Aldrich) to create reducing or alkylating environments respectively. Alkylation and hyperoxidation of PTPs were performed as described previously^20^, with the following modifications: (i) lysis buffers were degassed by bubbling 100% helium gas; (ii) zebrafish fin clips were sonicated in lysis buffer; (iii) buffer exchange before hyperoxidation was performed with 3 kDa centrifugal devices to 50 mM HEPES; (iv) hyperoxidation was performed overnight.

For mass spectrometry measurements, snap-frozen amputated caudal fin clips were divided equally into 3 conditions to measure: (1) total PTPs; (2) oxidized PTPs; or (3) control for efficiency of alkylation and baseline oxidation. In condition (1), fin clips were lysed and reduced in lysis buffer containing 10 mM DTT; in condition (2), fin clips were lysed and alkylated in lysis buffer containing 20 mM NEM for 30 min, then reduced in 20 mM DTT; and in condition (3), fin clips were lysed and reduced in lysis buffer containing 10 mM DTT, alkylated in 20 mM NEM for 30 min, then reduced again in 10 mM DTT. Subsequently, all three conditions were hyperoxidized with 1 mM PV (1 mM orthovanadate and 1 mM H_2_O_2_, mixed and left at room temperature for at least 5 min before use). Second fin clips were divided into the same 3 conditions and processed similarly. 10 fin clips per condition were used, and each of the 6 conditions, representing one biological replicate, performed in triplicate. Protein recovery after buffer exchange was >80%, such that 400 µg of fin lysate input material was obtained for each of 18 immunoprecipitations. For immunoblotting of hyperoxidized PTPs, 30 fin clips were used per condition per time point.

### Immunoprecipitation of hyperoxidized PTP motif peptides and PTP proteins

Mouse monoclonal antibody against oxidized PTP active site (R&D Systems) was cross-linked to protein A/G-coated sepharose beads (Santa Cruz Biotechnology) with 2.5 mM disuccinimidyl suberate (Thermo Fisher Scientific) to prevent antibody co-elution. Upon elution of PTP motif peptides, antibody-crosslinked beads were regenerated for sequential immunoprecipitations from the same sample, by re-using the flowthrough. For PTP motif peptide immunoprecipitations, fin lysates were digested sequentially at 37 °C in Lys C (1:100) and Trypsin (1:50) for 4 and 12 hours respectively, and diluted in 50 mM HEPES, pH 7.4. PTP motif peptides isolated from 5 sequential immunoprecipitations were further purified by strong cation exchange STAGE tips and analysed individually by LC-MS/MS as 5 technical replicates.

### LC-MS/MS analysis and database search

Immuno-precipitated PTP motif peptides were separated on a 40-min reverse-phase gradient on the UHPLC 1290 system (Agilent), and analysed on an Orbitrap Q Exactive HF mass spectrometer (Thermo Scientific). Peptides were first trapped on a 2 cm × 100 μm Reprosil C18 pre-column (3 μm) and then separated on a 50 cm × 75 μm Poroshell EC-C18 analytical column (2.7 μm). Trapping was performed for 5 min in 0.1 M acetic acid (Solvent A) and elution with 80% ACN in 0.1 M acetic acid (Solvent B) in gradients as follows: 13–44% solvent B in 20 min, 44–100% in 3 min and 100% for 2 min, before equilibrating back to Solvent A for another 10 min. Flow was passively split to 300 nl/min. MS data were obtained in data-dependent acquisition mode. Full scans were acquired in the m/z range of 375–1500 at the resolution of 60,000 (m/z 400), with AGC target 3E6 and a maximum injection time of 150ms. Top 7 most intense precursor ions were selected for HCD fragmentation performed at normalized collision energy (NCE) 27%, after accumulation to target value of 1E5, also in a maximum injection time of 150ms. MS/MS acquisition was performed at a resolution of 30,000, and dynamic exclusion was set to 6.0 s.

Raw spectral files were processed using MaxQuant version 1.5.3.30 and searched against the *Danio rerio* Uniprot database (version Feb 2017, including TrEMBL) using Andromeda. Cysteine carbamidomethylation was set to fixed modification, while variable modifications of methionine oxidation, cysteine hyperoxidation, as well as up to 2 missed cleavages were allowed. False discovery rate (FDR) was restricted to 1% in both protein and peptide identification.

### Genotyping

All embryos that were used in these assays were genotyped to establish *ptpra* or *ptpn11a* status. To this end, genomic zebrafish DNA was extracted through lysis of embryos in 100 µg/ml proteinase K (Sigma) diluted in SZL buffer (50 mM KCl, 2.5 mM MgCl, 10 mM Tris pH 8.3, 0.005% NP40, 0.005% Tween-20, and 10% 0.1% Gelatine). Lysis was performed by incubating at 60 °C for 1 hour, followed by 95 °C for 15 minutes in a thermal cycler (BioRad T100). Kompetitive Allele Specific PCR (KASP) using specific primers (Table S2) was carried out according to the manufacturer’s instructions (LGC Group) and a PHERAstar microplate reader (BMG LABTECH) and Klustercaller software were used to identify the mutations.

### Statistics

Semi-quantitative analysis of hyperoxidized PTP motif peptides was performed by spectral counting. Spectra obtained in five technical replicate MS runs were summed, and averaged across three biological replicates. Mean spectral count and student *t*-test statistics for difference between first and second fin clips are reported as exact two-tailed *p*-value (Table S2).

Histograms of whole data sets were examined to determine non-normal distribution of the data. Statistical analysis of unequal variances was obtained through a Kruskall-Wallis test. Differences between different experimental condition were assessed using a Mann-Whitney U test with a confidence level set to 99%. All tests were performed in SPSS (IBM). Differences were considered significant when *p* < 0.05 with a confidence interval greater than 99%.

## Electronic supplementary material

Supplementary information
