# Supplementary material for: Differential oxidation of protein-tyrosine phosphatases during zebrafish caudal fin regeneration
Source: Sci Rep. 2017 Aug 16;7:8460. doi: 10.1038/s41598-017-07109-8 (PMC5559610; doi:10.1038/s41598-017-07109-8)
Supplement: Supplementary file 1 — Supplementary information [file 41598_2017_7109_MOESM1_ESM.pdf]

Supplementary information

Title: Differential oxidation of protein-tyrosine phosphatases during zebrafish caudal fin regeneration

Authors: Wei Wu, Alexander James Hale, Simone Lemeer and Jeroen den Hertog

**Fig. S1. Sequence alignments and logos of all predicted PTP motif peptides in the zebrafish Uniprot+trEMBL database. (a)** Sequence alignment of 52 predicted catalytic sites ±20 flanking residues, representing 44 distinct PTPs, in the zebrafish Uniprot+trEMBL database (release Feb 2017). PTPs containing two catalytic sites are aligned as separate entries. Alignment generated with Boxshade 3.21, shading indicates sequence conservation >80%. **(b)** Sequence logo visualized with WebLogo, overall height of stack at each position indicates sequence conservation; height of symbols within the stack indicates relative frequency of each amino acid in the position.

a

```
                            -20                  0                   +20
A0A1L1QZV1                  LVNDVEEDRKGWTSSGPVVVHCSAGIGRTGCFMATTIGCRQLGLEG
F1QWY5_CA16B                LKFISRSTVTHASSTGPVLVHCSAGVGRTGTYIVIDSMLKQIKDRG
F1QDL0_PTPN1                FLFKVRESGCLSPELGPVVVHCSAGIGRSGTFCLVDTCLLLMSQRK
C7U151_PTPN11A              FLEEVKLKQEGITGAGPIVVHCSAGIGRTGTFIVIDILIDIIREKG
C7U120_PTPN11B              FLEQVNRTQSAIPESGPIVVHCSAGIGRTGTIIVIDILIDIINRQG
Q6PEI4_PTPN12               LDMIELMRKHQQRDTTPICVHCSAGCGRTGAICAIDYTWNLLKAGR
CU124_PTPN13                QLLTFISYMRHIHLSGPIITHCSAGIGRSGTLICIDVVLGLISKDA
C7U149_PTPN18               LGMMELARQKQGNQTDPVVIHCSAGCGRTGVICAVDYVNDLLLTEQ
F1QIX3_PTPN20               QLVRFIRYMRAVHAKGPITVHCSAGIAEPDPHLIT-----------
F1Q6T9_PTPN21               VRRHTNSSSDPKNTNLPVLVHCSAGVGRTGVVILSEIMIACLEHNE
C7U128_PTPN22               LELLQEMRIYQDNEEIPICIHCSAGCGRTGALCAIDYTWNLLKRQI
F1R1D1_PTPN23A              IQEVHGHYLLQRPLHTPVVVHCSSGVGRTGAFCLLYAALQEIEAGN
F1QXQ5_PTPN2A               FLFKVRESGSLGPENGPVVVHCSAGIGRSGSFSLVDTCLVLMDQRN
Q6NY86_PTPN2B               FLFKVRESGSLGMEQGPAVVHCSAGIGRSGTFSLVDTCLVLMDKRK
F1QLN8_PTPN3                FLDFVKSVRSMRQESVPLMVHCSAGICPMGVLITMETAMTPMEKEK
C7U127_PTPN4A               LDFVALVRNKRAGKDEPVVVHCSAGIGRTGVLITMETAMCLMECSQ
C7U113_PTPN4B               LNFVSQVRSKRADGPEPVVVHCSAGIGRTGVLITMRQRC-------
F1RCZ5_PTPN5                VQEVEEARKQAPPNSGPVIVHCSAGIGRTGCFIATSILCQQLSNEG
C7U118_PTPN6                FLDQVNRKQEELRSSAPIVIHCSAGIGRTGTIVVIDMLIDSIDAKG
X1WFV4_PTPN9A               AVQAFGTQWRGHPLGPPMVVHCSAGIGRTGTFCALDICLSQLQDVG
F1QTI4_PTPN9B               SLRTLYPDWTGPPGGPPVVVHCSAGIGRTGTFCTLDICLSRLEDIG
F1QAX7_PTPRA_1              LKFLKKVKNCNPQYAGPIMVHCSAGVGRTGTFIVIDAMLDMMGAER
F1QAX7_PTPRA_2              NIIAAVQKQQQQSGNHPITVHCSAGAGRTGTFCALSTVLERVKAEG
C7U135_PTPRB                FVRTVRDFINRTNSPGISVVHCSAGVGRTGTFIVLDRALQQLDRNC
C7U126_PTPRC                LKLRRRVNSFKNFFSGPIVVHCSAGVGRTGTYIGIDAMIESLEAEN
H9CWQ4_PTPRDB_1             LAFLRRVKACNPPDAGPIVVHCSAGVGRTGCFIVIDAMLERVKQEK
H9CWQ4_PTPRDB_2             FIGQVHKTKEQFGQDGPITVHCSAGVGRTGVFITLSIVLERMRYEG
E7EZG7_PTPREA_1             LKFLKKVKQVNPSYAGPIVVHCSAGVGRTGTFIVIDAMIDMMHTEQ
E7EZG7_PTPREA_2             DIIAAVQKQQQQSGNHPIIVHCSAGAGRTGTFIALSNILERVKAEG
F1RB37_PTPREB_1             LKFLKKVKSVNPAHAGPIIVHCSAGVGRTGTFIVIDAMMDMMHAEG
F1RB37_PTPREB_2             DIIAAVQKQQQQSGNNPIVVHCSAGAGRTGTFIALSNILERVKAEG
A4IFW2_PTPRF_1              LAFLRRVKACNPPDAGPMVVHCSAGVGRTGCFIVIDAMLERMKHEK
A4IFW2_PTPRF_2              FIGQVHKTKEQFGQDGPITVHCSAGVGRTGVFITLSIVLERMRYEG
Q90YJ5_PTPRFA_1             LAFLRRVKACNPPDAGPMVVHCSAGVGRTGCFIVIDAMLERMKHEK
Q90YJ5_PTPRFA_2             FIGQVHKTKEQFGQDGPITVHCSAGVGRTGVFITLSIVLERMRYEG
A0A0R4IGS0_PTPRFB_1         LAFLRRVKACNPPDAGPMVVHCSAGVGRTGCFIVIDAMLERMKHEK
A0A0R4IGS0_PTPRFB_2         FIGQVHKTKEQFGQDGPITVHCSAGVGRTGVFITLSIVLERMRYEG
A0A1B1LZI1_PTPRGA           LTFVRKSSAAQTPDMGPMLVHCSAGVGRTGTYIVIDSMLQQLKDKG
E7F9R2_PTPRH                FRDLIRQHIESHFSTGPTVVHCSAGVGRTGTLIALDVLLQQLNREK
B6HY54_PTPRJA               FRHLVREHMDEYSRHSPTLVHCSAGVGRTGTFIAIDRLIFQIERDG
X1WCM2_PTPRJB.1             FRHLVREHMDQYSRHSPTVVHCSAGVGRTGTFIAIDHLSFQIERDS
X1WF45_PTPRJB.2             FRHLVREHMNQYSCTSPTVVHCSAGVGRTGTFIAIDHLISQIERDS
X1WD65_PTPRK                LSFIRRVKMSNPPTAGPIVVHCSAGAGRTGCYIVIDIMLDMAEREG
A0A0R4IJG0_PTPRO            LQFVQIVRQQVNRSKGPIVVHCSAGVGRTGTFISLDRLMQHIQEHE
A8DZA4_PTPRQ                VQFVKAVRSNRGHENTTIVVHCSAGVGRTGVFIALDHLIQHLRDHE
F1QJW2_PTPRSA_1             LAFLRRVKTCNPPDAGPIIAHCSAGVGRTGCFIVIDAMLERIKHEK
F1QJW2_PTPRSA_2             FIGQVHKTKEQFGQDGPISVHCSAGVGRTGVFITLSIVLERMRYEG
F1QBL2_PTPRT                LGFIRQVKFLNPPDAGPIVVHCSAGAGRTGCFIAVDIMLDMAESEG
B3DK56_PTPRU                LAFIRRVKTSTPLDAGPVVVHCSVGAGRTGCYIVLDVMLDMAECEG
C7U125_PTPRUB               LAFIRRVKTSTPLDAGPVVVHCSVGAGRTGCYIVLDVMLDMAECEG
F1QZX7_PTPRZ1A              LSFVYKSSKAQTDGMGPMVVHCSAGVGRTGTYIVLDSMLKQIKEKG
B8A5J4_PTPRZ1B              LSFIRTSSQARTAEMGPVVVHCSAGVGRTGTYIVIDSMLKQIKEEN
consensus                                   ....***.*.....  . .   .  .
```

b

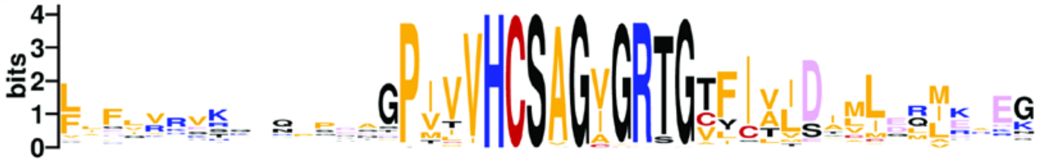

**Fig. S2. Representative raw spectrum for each zebrafish PTP catalytic site detected.**

| Uniprot Accession | Description | Gene name | PTP type | Sequence | Hyperoxidised cysteine | Total MS/MS count |
|---|---|---|---|---|---|---|
| B3DJL5_site1 (C7U129) | Ptpra protein | ptpra | Receptor | QQQQSGNHPITVHcSAGAGR | C14(hyper) | 76 |

Peptide Summary

Sequence: QQQQSGNHPITVHCSAGAGR, C14-hyperoxidation (47.98474 Da)
Charge: +2, Monoisotopic m/z: 1062.48486 Da (-0.58 mmu/-0.55 ppm), MH+: 2123.96245 Da, RT: 12.88 min,
Identified with: Mascot (v1.30); IonScore:113, Exp Value:6.1E-010, Ions matched by search engine: 14/210
Fragment match tolerance used for search: 0.05 Da

Fragment Matches

Value Type: Theo. Mass [Da]

Ion Series | Neutral Losses | Precursor Ions

| #1 | $b^+$ | $b^{2+}$ | Seq. | $y^+$ | $y^{2+}$ | #2 |
|---|---|---|---|---|---|---|
| 1 | 129.06586 | 65.03657 | Q | | | 20 |
| 2 | 257.12444 | 129.06586 | Q | 1995.90504 | 998.45616 | 19 |
| 3 | 385.18302 | 193.09515 | Q | 1867.84646 | 934.42687 | 18 |
| 4 | 513.24160 | 257.12444 | Q | 1739.78788 | 870.39758 | 17 |
| 5 | 600.27363 | 300.64045 | S | 1611.72930 | 806.36829 | 16 |
| 6 | 657.29510 | 329.15119 | G | 1524.69727 | 762.85227 | 15 |
| 7 | 771.33803 | 386.17265 | N | 1467.67580 | 734.34154 | 14 |
| 8 | 908.39694 | 454.70211 | H | 1353.63287 | 677.32007 | 13 |
| 9 | 1005.44971 | 503.22849 | P | 1216.57396 | 608.79062 | 12 |
| 10 | 1118.53378 | 559.77053 | I | 1119.52119 | 560.26423 | 11 |
| 11 | 1219.58146 | 610.29437 | T | 1006.43712 | 503.72220 | 10 |
| 12 | 1318.64988 | 659.82858 | V | 905.38944 | 453.19836 | 9 |
| 13 | 1455.70879 | 728.35803 | H | 806.32102 | 403.66415 | 8 |
| 14 | 1606.70272 | 803.85500 | C-hyper | 669.26211 | 335.13469 | 7 |
| 15 | 1693.73475 | 847.37101 | S | 518.26817 | 259.63772 | 6 |
| 16 | 1764.77187 | 882.88957 | A | 431.23614 | 216.12171 | 5 |
| 17 | 1821.79334 | 911.40031 | G | 360.19902 | 180.60315 | 4 |
| 18 | 1892.83046 | 946.91887 | A | 303.17755 | 152.09241 | 3 |
| 19 | 1949.85193 | 975.42960 | G | 232.14043 | 116.57385 | 2 |
| 20 | | | R | 175.11896 | 88.06312 | 1 |

Fragment Spectrum

Extracted from: D:\wu000024\20161117_Fin05_3reps\Set2\20161118_OR12_A6_WW_Fin05_10_1.raw #3556 RT: 12.88
FTMS, HCD@27.00, z=+2, Mono m/z=1062.48486 Da, MH+=2123.96245 Da, Match Tol.=0.05 Da

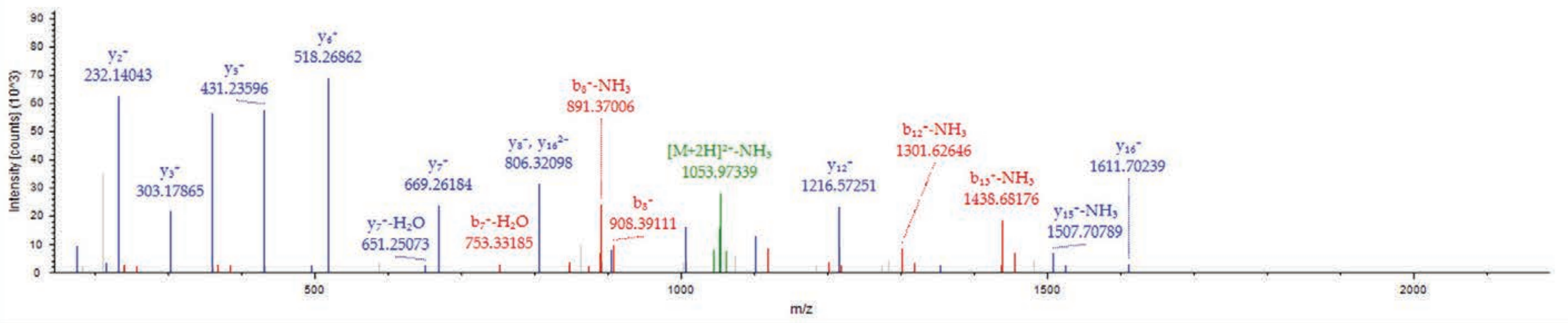

| Uniprot Accession | Description | Gene name | PTP type | Sequence | Hyperoxidised cysteine | Total MS/MS count |
|---|---|---|---|---|---|---|
| B3DJL5_site2 (C7U129) | Ptpra protein | ptpra | Receptor | NcNPQYAGPIV**VHcSAGVGR** | C14(hyper) | 33 |

Peptide Summary

Sequence: NCNPQYAGPIVVHCSAGVGR, C2-hyperoxidation (47.98474 Da), C14-hyperoxidation (47.98474 Da)
Charge: +2, Monoisotopic m/z: 1069.47363 Da (+0.47 mmu/+0.44 ppm), MH+: 2137.93999 Da, RT: 19.30 min,
Identified with: Mascot (v1.30); IonScore:108, Exp Value:1.3E-009, Ions matched by search engine: 11/190
Fragment match tolerance used for search: 0.05 Da

Fragment Matches

Value Type: Theo. Mass [Da]

Ion Series | Neutral Losses | Precursor Ions

| #1 | b+ | b2+ | Seq. | y+ | y2+ | #2 |
|---|---|---|---|---|---|---|
| 1 | 115.05021 | 58.02874 | N | | | 20 |
| 2 | 266.04414 | 133.52571 | C-hyper | 2023.89612 | 1012.45170 | 19 |
| 3 | 380.08707 | 190.54717 | N | 1872.90219 | 936.95473 | 18 |
| 4 | 477.13984 | 239.07356 | P | 1758.85926 | 879.93327 | 17 |
| 5 | 605.19842 | 303.10285 | Q | 1661.80649 | 831.40688 | 16 |
| 6 | 768.26174 | 384.63451 | Y | 1533.74791 | 767.37759 | 15 |
| 7 | 839.29886 | 420.15307 | A | 1370.68459 | 685.84593 | 14 |
| 8 | 896.32033 | 448.66380 | G | 1299.64747 | 650.32737 | 13 |
| 9 | 993.37310 | 497.19019 | P | 1242.62600 | 621.81664 | 12 |
| 10 | 1106.45717 | 553.73222 | I | 1145.57323 | 573.29025 | 11 |
| 11 | 1205.52559 | 603.26643 | V | 1032.48916 | 516.74822 | 10 |
| 12 | 1304.59401 | 652.80064 | V | 933.42074 | 467.21401 | 9 |
| 13 | 1441.65292 | 721.33010 | H | 834.35232 | 417.67980 | 8 |
| 14 | 1592.64685 | 796.82707 | C-hyper | 697.29341 | 349.15034 | 7 |
| 15 | 1679.67888 | 840.34308 | S | 546.29947 | 273.65337 | 6 |
| 16 | 1750.71600 | 875.86164 | A | 459.26744 | 230.13736 | 5 |
| 17 | 1807.73747 | 904.37238 | G | 388.23032 | 194.61880 | 4 |
| 18 | 1906.80589 | 953.90659 | V | 331.20885 | 166.10806 | 3 |
| 19 | 1963.82736 | 982.41732 | G | 232.14043 | 116.57385 | 2 |
| 20 | | | R | 175.11896 | 88.06312 | 1 |

Fragment Spectrum

Extracted from: D:\wu000024\20161117_Fin05_3reps\Set3\20161120_OR12_A6_WW_Fin05_13_4.raw #6125 RT: 19.30
FTMS, HCD@27.00, z=+2, Mono m/z=1069.47363 Da, MH+=2137.93999 Da, Match Tol.=0.05 Da

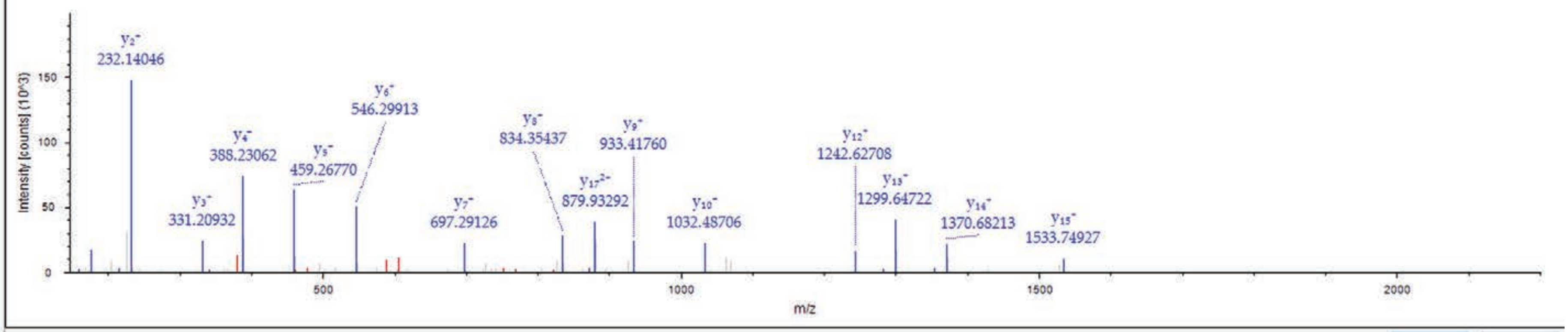

| Uniprot Accession | Description | Gene name | PTP type | Sequence | Hyperoxidised cysteine | Total MS/MS count |
|---|---|---|---|---|---|---|
| A0A0R4ITJ9 (C7U135) | Uncharacterized protein | ptprb | Receptor | TNSPGISVVHcSAGVGR | C11(hyper) | 51 |

Peptide Summary

Sequence: TNSPGISVVHCSAGVGR, C11-hyperoxidation (47.98474 Da)
Charge: +2, Monoisotopic m/z: 844.90533 Da (+0.63 mmu/+0.74 ppm), MH+: 1688.80339 Da, RT: 17.09 min,
Identified with: Mascot (v1.30); IonScore:108, Exp Value:2.2E-009, Ions matched by search engine: 14/180
Fragment match tolerance used for search: 0.05 Da

Fragment Matches

Value Type: Theo. Mass [Da]

Ion Series | Neutral Losses | Precursor Ions

| #1 | $b^{+}$ | $b^{2+}$ | Seq. | $y^{+}$ | $y^{2+}$ | #2 |
|---|---|---|---|---|---|---|
| 1 | 102.05496 | 51.53112 | T | | | 17 |
| 2 | 216.09789 | 108.55258 | N | 1587.75446 | 794.38087 | 16 |
| 3 | 303.12992 | 152.06860 | S | 1473.71153 | 737.35940 | 15 |
| 4 | 400.18269 | 200.59498 | P | 1386.67950 | 693.84339 | 14 |
| 5 | 457.20416 | 229.10572 | G | 1289.62673 | 645.31700 | 13 |
| 6 | 570.28823 | 285.64775 | I | 1232.60526 | 616.80627 | 12 |
| 7 | 657.32026 | 329.16377 | S | 1119.52119 | 560.26423 | 11 |
| 8 | 756.38868 | 378.69798 | V | 1032.48916 | 516.74822 | 10 |
| 9 | 855.45710 | 428.23219 | V | 933.42074 | 467.21401 | 9 |
| 10 | 992.51601 | 496.76164 | H | 834.35232 | 417.67980 | 8 |
| 11 | 1143.50994 | 572.25861 | C-hyper | 697.29341 | 349.15034 | 7 |
| 12 | 1230.54197 | 615.77462 | S | 546.29947 | 273.65337 | 6 |
| 13 | 1301.57909 | 651.29318 | A | 459.26744 | 230.13736 | 5 |
| 14 | 1358.60056 | 679.80392 | G | 388.23032 | 194.61880 | 4 |
| 15 | 1457.66898 | 729.33813 | V | 331.20885 | 166.10806 | 3 |
| 16 | 1514.69045 | 757.84886 | G | 232.14043 | 116.57385 | 2 |
| 17 | | | R | 175.11896 | 88.06312 | 1 |

Fragment Spectrum

Extracted from: D:\wu000024\20161117_Fin05_3reps\Set3\20161120_OR12_A6_WW_Fin05_13_1.raw #5281 RT: 17.09
FTMS, HCD@27.00, z=+2, Mono m/z=844.90533 Da, MH+=1688.80339 Da, Match Tol.=0.05 Da

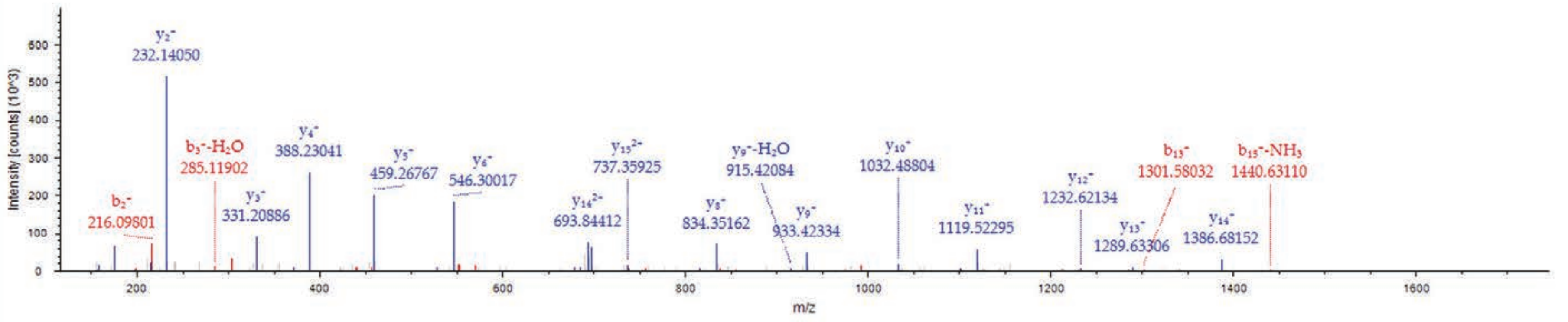

| Uniprot Accession | Description | Gene name | PTP type | Sequence | Hyperoxidised cysteine | Total MS/MS count |
|---|---|---|---|---|---|---|
| C7U126 | CD45 antigen (Fragment) | ptprc | Receptor | NFFSGPIVVHcSAGVGR | C11(hyper) | 93 |

**Peptide Summary**

Sequence: NFFSGPIVVHCSAGVGR, C11-hyperoxidation (47.98474 Da)
Charge: +2, Monoisotopic m/z: 897.93384 Da (+0.57 mmu/+0.63 ppm), MH+: 1794.86040 Da, RT: 24.10 min,
Identified with: Mascot (v1.30); IonScore:129, Exp Value:1.8E-011, Ions matched by search engine: 15/176
Fragment match tolerance used for search: 0.05 Da

**Fragment Matches**

Value Type: Theo. Mass [Da]

Ion Series | Neutral Losses | Precursor Ions

| #1 | $b^+$ | $b^{2+}$ | Seq. | $y^+$ | $y^{2+}$ | #2 |
|---|---|---|---|---|---|---|
| 1 | 115.05021 | 58.02874 | N | | | 17 |
| 2 | 262.11863 | 131.56295 | F | 1680.81634 | 840.91181 | 16 |
| 3 | 409.18705 | 205.09716 | F | 1533.74792 | 767.37760 | 15 |
| 4 | 496.21908 | 248.61318 | S | 1386.67950 | 693.84339 | 14 |
| 5 | 553.24055 | 277.12391 | G | 1299.64747 | 650.32737 | 13 |
| 6 | 650.29332 | 325.65030 | P | 1242.62600 | 621.81664 | 12 |
| 7 | 763.37739 | 382.19233 | I | 1145.57323 | 573.29025 | 11 |
| 8 | 862.44581 | 431.72654 | V | 1032.48916 | 516.74822 | 10 |
| 9 | 961.51423 | 481.26075 | V | 933.42074 | 467.21401 | 9 |
| 10 | 1098.57314 | 549.79021 | H | 834.35232 | 417.67980 | 8 |
| 11 | 1249.56707 | 625.28717 | C-hyper | 697.29341 | 349.15034 | 7 |
| 12 | 1336.59910 | 668.80319 | S | 546.29947 | 273.65337 | 6 |
| 13 | 1407.63622 | 704.32175 | A | 459.26744 | 230.13736 | 5 |
| 14 | 1464.65769 | 732.83248 | G | 388.23032 | 194.61880 | 4 |
| 15 | 1563.72611 | 782.36669 | V | 331.20885 | 166.10806 | 3 |
| 16 | 1620.74758 | 810.87743 | G | 232.14043 | 116.57385 | 2 |
| 17 | | | R | 175.11896 | 88.06312 | 1 |

**Fragment Spectrum**

Extracted from: D:\wu000024\20161117_Fin05_3reps\Set3\20161120_OR12_A6_WW_Fin05_13_1.raw #7879 RT: 24.10
FTMS, HCD@27.00, z=+2, Mono m/z=897.93384 Da, MH+=1794.86040 Da, Match Tol.=0.05 Da

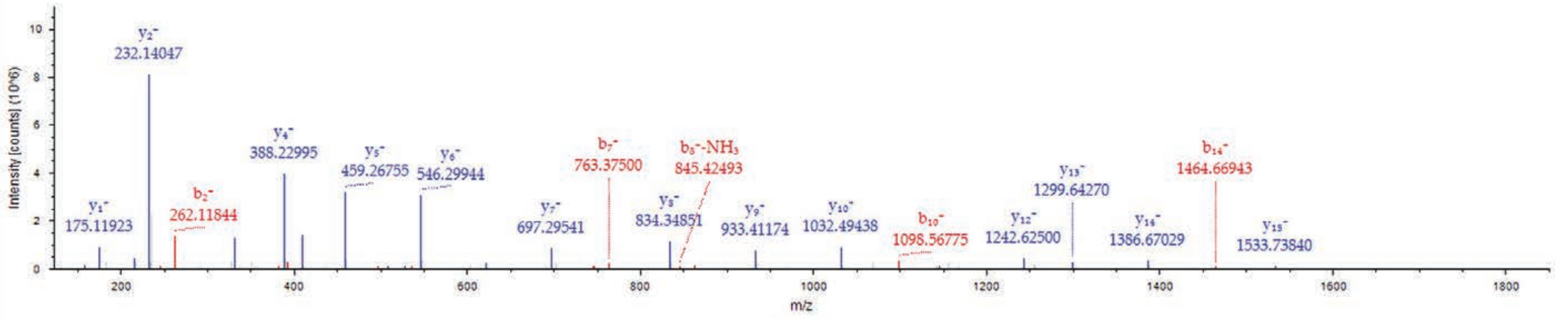

| Uniprot Accession | Description | Gene name | PTP type | Sequence | Hyperoxidised cysteine | Total MS/MS count |
|---|---|---|---|---|---|---|
| E7EZG7_site1 | Receptor-type tyrosine-protein phosphatase | ptprea | Receptor | QVNPSYAGPIV**VHcSAGVGR** | C14(hyper) | 35 |

**Peptide Summary**

Sequence: QVNPSYAGPIVVHCSAGVGR, C14-hyperoxidation (47.98474 Da)
Charge: +2, Monoisotopic m/z: 1030.00635 Da (+1.39 mmu/+1.35 ppm), MH+: 2059.00542 Da, RT: 19.28 min,
Identified with: Mascot (v1.30); IonScore:143, Exp Value:7.5E-013, Ions matched by search engine: 14/210
Fragment match tolerance used for search: 0.05 Da

**Fragment Matches**

Value Type: Theo. Mass [Da]

Ion Series | Neutral Losses | Precursor Ions

| #1 | $b^+$ | $b^{2+}$ | Seq. | $y^+$ | $y^{2+}$ | #2 |
|---|---|---|---|---|---|---|
| 1 | 129.06586 | 65.03657 | Q | | | 20 |
| 2 | 228.13428 | 114.57078 | V | 1930.94406 | 965.97567 | 19 |
| 3 | 342.17721 | 171.59224 | N | 1831.87564 | 916.44146 | 18 |
| 4 | 439.22998 | 220.11863 | P | 1717.83271 | 859.41999 | 17 |
| 5 | 526.26201 | 263.63464 | S | 1620.77994 | 810.89361 | 16 |
| 6 | 689.32533 | 345.16630 | Y | 1533.74791 | 767.37759 | 15 |
| 7 | 760.36245 | 380.68486 | A | 1370.68459 | 685.84593 | 14 |
| 8 | 817.38392 | 409.19560 | G | 1299.64747 | 650.32737 | 13 |
| 9 | 914.43669 | 457.72198 | P | 1242.62600 | 621.81664 | 12 |
| 10 | 1027.52076 | 514.26402 | I | 1145.57323 | 573.29025 | 11 |
| 11 | 1126.58918 | 563.79823 | V | 1032.48916 | 516.74822 | 10 |
| 12 | 1225.65760 | 613.33244 | V | 933.42074 | 467.21401 | 9 |
| 13 | 1362.71651 | 681.86189 | H | 834.35232 | 417.67980 | 8 |
| 14 | 1513.71044 | 757.35886 | C-hyper | 697.29341 | 349.15034 | 7 |
| 15 | 1600.74247 | 800.87487 | S | 546.29947 | 273.65337 | 6 |
| 16 | 1671.77959 | 836.39343 | A | 459.26744 | 230.13736 | 5 |
| 17 | 1728.80106 | 864.90417 | G | 388.23032 | 194.61880 | 4 |
| 18 | 1827.86948 | 914.43838 | V | 331.20885 | 166.10806 | 3 |
| 19 | 1884.89095 | 942.94911 | G | 232.14043 | 116.57385 | 2 |
| 20 | | | R | 175.11896 | 88.06312 | 1 |

**Fragment Spectrum**

Extracted from: D:\wu000024\20161117_Fin05_3reps\Set2\20161118_OR12_A6_WW_Fin05_10_5.raw #6044 RT: 19.28
FTMS, HCD@27.00, z=+2, Mono m/z=1030.00635 Da, MH+=2059.00542 Da, Match Tol.=0.05 Da

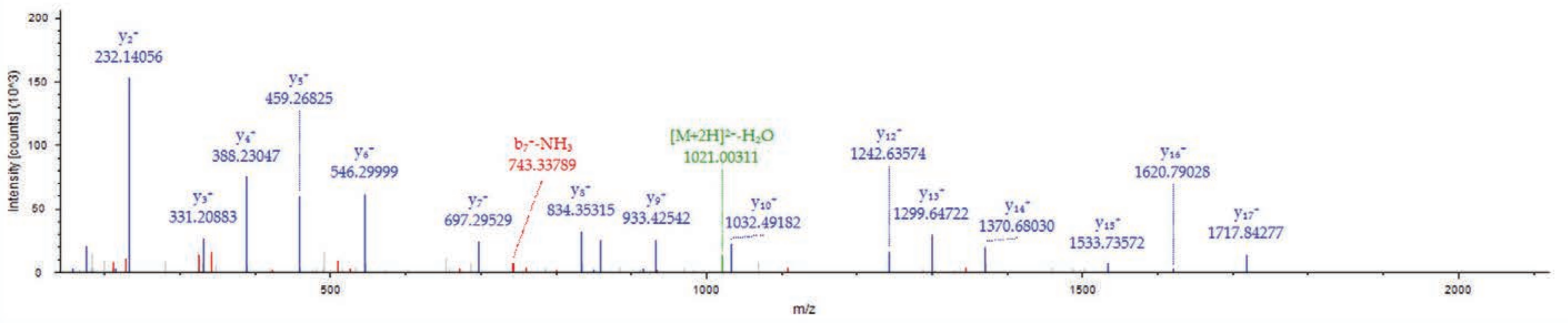

| Uniprot Accession | Description | Gene name | PTP type | Sequence | Hyperoxidised cysteine | Total MS/MS count |
|---|---|---|---|---|---|---|
| E7EZG7_site2 | Receptor-type tyrosine-protein phosphatase | ptprea | Receptor | QQQQSGNHPIIVHcSAGAGR | C14(hyper) | 119 |

Peptide Summary

Sequence: QQQQSGNHPIIVHCSAGAGR, C14-hyperoxidation (47.98474 Da)
Charge: +2, Monoisotopic m/z: 1068.50427 Da (+0.63 mmu/+0.59 ppm), MH+: 2136.00127 Da, RT: 13.63 min,
Identified with: Mascot (v1.30); IonScore:103, Exp Value:7.1E-009, Ions matched by search engine: 13/210
Fragment match tolerance used for search: 0.05 Da

Fragment Matches

Value Type: Theo. Mass [Da]

Ion Series | Neutral Losses | Precursor Ions

| #1 | $b^+$ | $b^{2+}$ | Seq. | $y^+$ | $y^{2+}$ | #2 |
|---|---|---|---|---|---|---|
| 1 | 129.06586 | 65.03657 | Q | | | 20 |
| 2 | 257.12444 | 129.06586 | Q | 2007.94143 | 1004.47435 | 19 |
| 3 | 385.18302 | 193.09515 | Q | 1879.88285 | 940.44506 | 18 |
| 4 | 513.24160 | 257.12444 | Q | 1751.82427 | 876.41577 | 17 |
| 5 | 600.27363 | 300.64045 | S | 1623.76569 | 812.38648 | 16 |
| 6 | 657.29510 | 329.15119 | G | 1536.73366 | 768.87047 | 15 |
| 7 | 771.33803 | 386.17265 | N | 1479.71219 | 740.35973 | 14 |
| 8 | 908.39694 | 454.70211 | H | 1365.66926 | 683.33827 | 13 |
| 9 | 1005.44971 | 503.22849 | P | 1228.61035 | 614.80881 | 12 |
| 10 | 1118.53378 | 559.77053 | I | 1131.55758 | 566.28243 | 11 |
| 11 | 1231.61785 | 616.31256 | I | 1018.47351 | 509.74039 | 10 |
| 12 | 1330.68627 | 665.84677 | V | 905.38944 | 453.19836 | 9 |
| 13 | 1467.74518 | 734.37623 | H | 806.32102 | 403.66415 | 8 |
| 14 | 1618.73911 | 809.87319 | C-hyper | 669.26211 | 335.13469 | 7 |
| 15 | 1705.77114 | 853.38921 | S | 518.26817 | 259.63772 | 6 |
| 16 | 1776.80826 | 888.90777 | A | 431.23614 | 216.12171 | 5 |
| 17 | 1833.82973 | 917.41850 | G | 360.19902 | 180.60315 | 4 |
| 18 | 1904.86685 | 952.93706 | A | 303.17755 | 152.09241 | 3 |
| 19 | 1961.88832 | 981.44780 | G | 232.14043 | 116.57385 | 2 |
| 20 | | | R | 175.11896 | 88.06312 | 1 |

Fragment Spectrum

Extracted from: D:\wu000024\20161117_Fin05_3reps\Set3\20161120_OR12_A6_WW_Fin05_13_1.raw #4030 RT: 13.63
FTMS, HCD@27.00, z=+2, Mono m/z=1068.50427 Da, MH+=2136.00127 Da, Match Tol.=0.05 Da

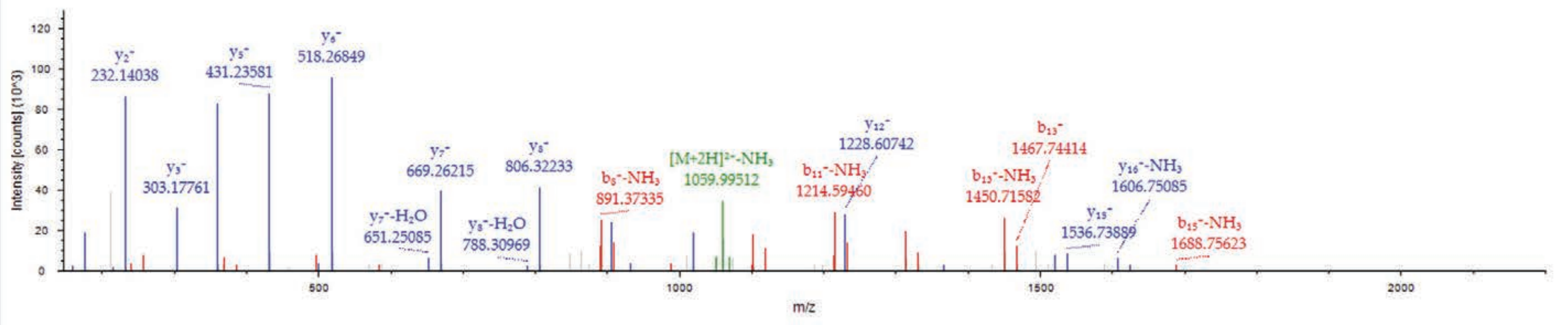

| Uniprot Accession | Description | Gene name | PTP type | Sequence | Hyperoxidised cysteine | Total MS/MS count |
|---|---|---|---|---|---|---|
| Q90YJ5_site1 | Receptor protein-tyrosine phosphatase LAR (Fragment) | ptprfa | Receptor | AcNPPDAGPMVVHcSAGVGR | C14(hyper) | 100 |

**Peptide Summary**

Sequence: ACNPPDAGPMVVHCSAGVGR, C2-hyperoxidation (47.98474 Da), C14-hyperoxidation (47.98474 Da)
Charge: +2, Monoisotopic m/z: 1017.42896 Da (+1.57 mmu/+1.55 ppm), MH+: 2033.85063 Da, RT: 17.82 min,
Identified with: Mascot (v1.30); IonScore:88, Exp Value:9.7E-008, Ions matched by search engine: 14/204
Fragment match tolerance used for search: 0.05 Da

**Fragment Matches**

Value Type: Theo. Mass [Da]

Ion Series | Neutral Losses | Precursor Ions

| #1 | $b^+$ | $b^{2+}$ | Seq. | $y^+$ | $y^{2+}$ | #2 |
|---|---|---|---|---|---|---|
| 1 | 72.04440 | 36.52584 | A | | | 20 |
| 2 | 223.03833 | 112.02280 | C-hyper | 1962.81037 | 981.90882 | 19 |
| 3 | 337.08126 | 169.04427 | N | 1811.81644 | 906.41186 | 18 |
| 4 | 434.13403 | 217.57065 | P | 1697.77351 | 849.39039 | 17 |
| 5 | 531.18680 | 266.09704 | P | 1600.72074 | 800.86401 | 16 |
| 6 | 646.21375 | 323.61051 | D | 1503.66797 | 752.33762 | 15 |
| 7 | 717.25087 | 359.12907 | A | 1388.64102 | 694.82415 | 14 |
| 8 | 774.27234 | 387.63981 | G | 1317.60390 | 659.30559 | 13 |
| 9 | 871.32511 | 436.16619 | P | 1260.58243 | 630.79485 | 12 |
| 10 | 1002.36561 | 501.68644 | M | 1163.52966 | 582.26847 | 11 |
| 11 | 1101.43403 | 551.22065 | V | 1032.48916 | 516.74822 | 10 |
| 12 | 1200.50245 | 600.75486 | V | 933.42074 | 467.21401 | 9 |
| 13 | 1337.56136 | 669.28432 | H | 834.35232 | 417.67980 | 8 |
| 14 | 1488.55529 | 744.78129 | C-hyper | 697.29341 | 349.15034 | 7 |
| 15 | 1575.58732 | 788.29730 | S | 546.29947 | 273.65337 | 6 |
| 16 | 1646.62444 | 823.81586 | A | 459.26744 | 230.13736 | 5 |
| 17 | 1703.64591 | 852.32660 | G | 388.23032 | 194.61880 | 4 |
| 18 | 1802.71433 | 901.86081 | V | 331.20885 | 166.10806 | 3 |
| 19 | 1859.73580 | 930.37154 | G | 232.14043 | 116.57385 | 2 |
| 20 | | | R | 175.11896 | 88.06312 | 1 |

**Fragment Spectrum**

Extracted from: D:\wu000024\20161117_Fin05_3reps\Set2\20161118_OR12_A6_WW_Fin05_10_1.raw #5339 RT: 17.82
FTMS, HCD@27.00, z=+2, Mono m/z=1017.42896 Da, MH+=2033.85063 Da, Match Tol.=0.05 Da

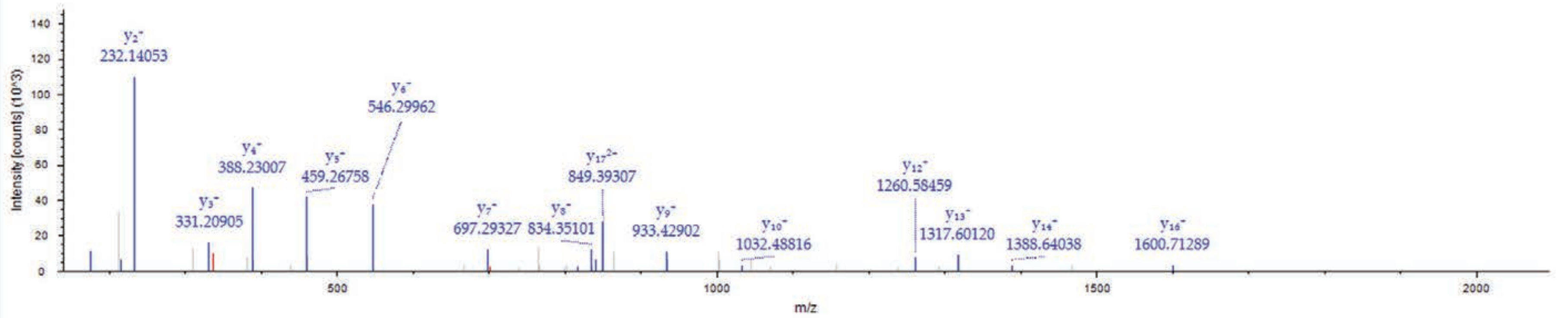

| Uniprot Accession | Description | Gene name | PTP type | Sequence | Hyperoxidised cysteine | Total MS/MS count |
|---|---|---|---|---|---|---|
| Q90YJ5_site2 | Receptor protein-tyrosine phosphatase LAR (Fragment) | ptprfa | Receptor | EQFGQDGPIT**VHcSAGVGR** | C13(hyper) | 72 |

Peptide Summary

Sequence: EQFGQDGPITVHCSAGVGR, C13-hyperoxidation (47.98474 Da)
Charge: +2, Monoisotopic m/z: 1003.45660 Da (+1.3 mmu/+1.3 ppm), MH+: 2005.90593 Da, RT: 18.24 min,
Identified with: Mascot (v1.30); IonScore:124, Exp Value:4.3E-011, Ions matched by search engine: 15/204
Fragment match tolerance used for search: 0.05 Da

Fragment Matches

Value Type: Theo. Mass [Da]

Ion Series | Neutral Losses | Precursor Ions

| #1 | $b^+$ | $b^{2+}$ | Seq. | $y^+$ | $y^{2+}$ | #2 |
|---|---|---|---|---|---|---|
| 1 | 130.04988 | 65.52858 | E | | | 19 |
| 2 | 258.10846 | 129.55787 | Q | 1876.86073 | 938.93400 | 18 |
| 3 | 405.17688 | 203.09208 | F | 1748.80215 | 874.90471 | 17 |
| 4 | 462.19835 | 231.60281 | G | 1601.73373 | 801.37050 | 16 |
| 5 | 590.25693 | 295.63210 | Q | 1544.71226 | 772.85977 | 15 |
| 6 | 705.28388 | 353.14558 | D | 1416.65368 | 708.83048 | 14 |
| 7 | 762.30535 | 381.65631 | G | 1301.62673 | 651.31700 | 13 |
| 8 | 859.35812 | 430.18270 | P | 1244.60526 | 622.80627 | 12 |
| 9 | 972.44219 | 486.72473 | I | 1147.55249 | 574.27988 | 11 |
| 10 | 1073.48987 | 537.24857 | T | 1034.46842 | 517.73785 | 10 |
| 11 | 1172.55829 | 586.78278 | V | 933.42074 | 467.21401 | 9 |
| 12 | 1309.61720 | 655.31224 | H | 834.35232 | 417.67980 | 8 |
| 13 | 1460.61113 | 730.80920 | C-hyper | 697.29341 | 349.15034 | 7 |
| 14 | 1547.64316 | 774.32522 | S | 546.29947 | 273.65337 | 6 |
| 15 | 1618.68028 | 809.84378 | A | 459.26744 | 230.13736 | 5 |
| 16 | 1675.70175 | 838.35451 | G | 388.23032 | 194.61880 | 4 |
| 17 | 1774.77017 | 887.88872 | V | 331.20885 | 166.10806 | 3 |
| 18 | 1831.79164 | 916.39946 | G | 232.14043 | 116.57385 | 2 |
| 19 | | | R | 175.11896 | 88.06312 | 1 |

Fragment Spectrum

Extracted from: D:\wu000024\20161117_Fin05_3reps\Set2\20161118_OR12_A6_WW_Fin05_7_1.raw #5734 RT: 18.24
FTMS, HCD@27.00, z=+2, Mono m/z=1003.45660 Da, MH+=2005.90593 Da, Match Tol.=0.05 Da

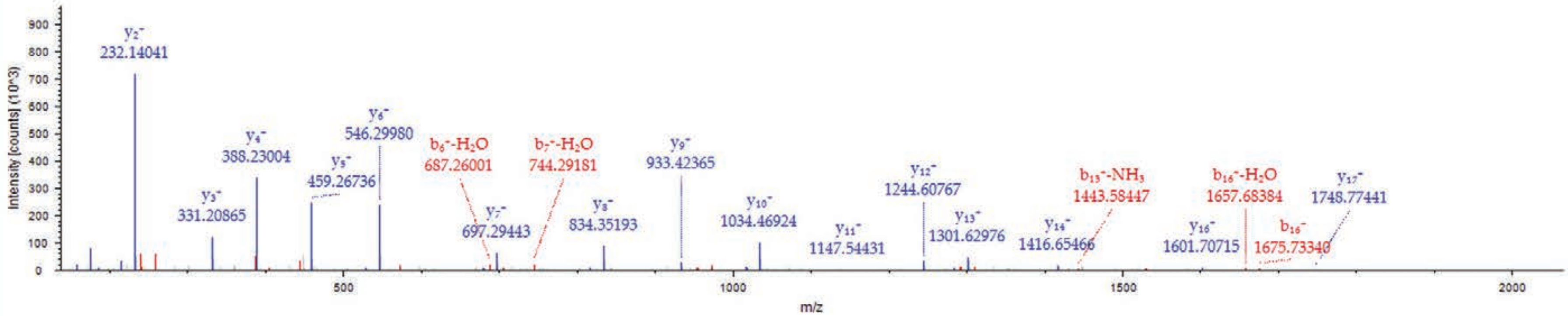

| Uniprot Accession | Description | Gene name | PTP type | Sequence | Hyperoxidised cysteine | Total MS/MS count |
|---|---|---|---|---|---|---|
| F1Q4V8 | Uncharacterized protein (Fragment) | ptprga | Receptor | SSAAQTPDMGPMLVHcSAGVGR | C16(hyper) | 42 |

Peptide Summary

Sequence: SSAAQTPDMGPMLVHCSAGVGR, C16-hyperoxidation (47.98474 Da)
Charge: +2, Monoisotopic m/z: 1110.49768 Da (+1.88 mmu/+1.69 ppm), MH+: 2219.98808 Da, RT: 20.67 min,
Identified with: Mascot (v1.30); IonScore:125, Exp Value:3.7E-011, Ions matched by search engine: 16/234
Fragment match tolerance used for search: 0.05 Da

Fragment Matches

Value Type: Theo. Mass [Da]

Ion Series | Neutral Losses | Precursor Ions

| #1 | $b^+$ | $b^{2+}$ | Seq. | $y^+$ | $y^{2+}$ | #2 |
|---|---|---|---|---|---|---|
| 1 | 88.03931 | 44.52329 | S | | | 22 |
| 2 | 175.07134 | 88.03931 | S | 2132.95230 | 1066.97979 | 21 |
| 3 | 246.10846 | 123.55787 | A | 2045.92027 | 1023.46377 | 20 |
| 4 | 317.14558 | 159.07643 | A | 1974.88315 | 987.94521 | 19 |
| 5 | 445.20416 | 223.10572 | Q | 1903.84603 | 952.42665 | 18 |
| 6 | 546.25184 | 273.62956 | T | 1775.78745 | 888.39736 | 17 |
| 7 | 643.30461 | 322.15594 | P | 1674.73977 | 837.87352 | 16 |
| 8 | 758.33156 | 379.66942 | D | 1577.68700 | 789.34714 | 15 |
| 9 | 889.37206 | 445.18967 | M | 1462.66005 | 731.83366 | 14 |
| 10 | 946.39353 | 473.70040 | G | 1331.61955 | 666.31341 | 13 |
| 11 | 1043.44630 | 522.22679 | P | 1274.59808 | 637.80268 | 12 |
| 12 | 1174.48680 | 587.74704 | M | 1177.54531 | 589.27629 | 11 |
| 13 | 1287.57087 | 644.28907 | L | 1046.50481 | 523.75604 | 10 |
| 14 | 1386.63929 | 693.82328 | V | 933.42074 | 467.21401 | 9 |
| 15 | 1523.69820 | 762.35274 | H | 834.35232 | 417.67980 | 8 |
| 16 | 1674.69213 | 837.84970 | C-hyper | 697.29341 | 349.15034 | 7 |
| 17 | 1761.72416 | 881.36572 | S | 546.29947 | 273.65337 | 6 |
| 18 | 1832.76128 | 916.88428 | A | 459.26744 | 230.13736 | 5 |
| 19 | 1889.78275 | 945.39501 | G | 388.23032 | 194.61880 | 4 |
| 20 | 1988.85117 | 994.92922 | V | 331.20885 | 166.10806 | 3 |
| 21 | 2045.87264 | 1023.43996 | G | 232.14043 | 116.57385 | 2 |
| 22 | | | R | 175.11896 | 88.06312 | 1 |

Fragment Spectrum

Extracted from: D:\wu000024\20161117_Fin05_3reps\Set2\20161118_OR12_A6_WW_Fin05_10_1.raw #6417 RT: 20.67
FTMS, HCD@27.00, z=+2, Mono m/z=1110.49768 Da, MH+=2219.98808 Da, Match Tol.=0.05 Da

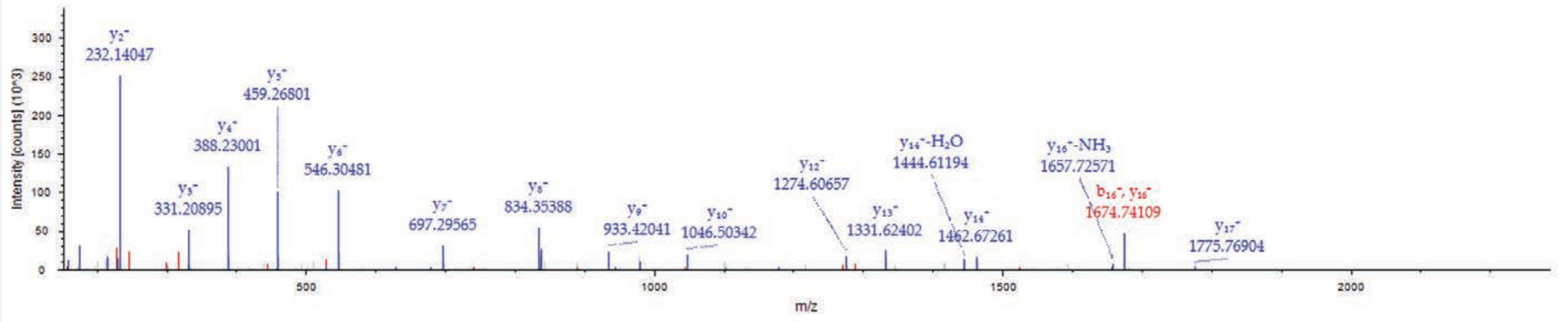

| Uniprot Accession | Description | Gene name | PTP type | Sequence | Hyperoxidised cysteine | Total MS/MS count |
|---|---|---|---|---|---|---|
| E7F9R2 | Uncharacterized protein | ptprh | Receptor | QHIESHFSTGPTVVHcSAGVGR | C16(hyper) | 159 |

Peptide Summary

Sequence: QHIESHFSTGPTVVHCSAGVGR, C16-hyperoxidation (47.98474 Da)
Charge: +3, Monoisotopic m/z: 785.36920 Da (-0.42 mmu/-0.53 ppm), MH+: 2354.09305 Da, RT: 16.03 min,
Identified with: Mascot (v1.30); IonScore:76, Exp Value:3.4E-006, Ions matched by search engine: 11/236
Fragment match tolerance used for search: 0.05 Da

Fragment Matches

Value Type: Theo. Mass [Da]

Ion Series | Neutral Losses | Precursor Ions

| #1 | $b^+$ | $b^{2+}$ | $b^{3+}$ | Seq. | $y^+$ | $y^{2+}$ | $y^{3+}$ | #2 |
|---|---|---|---|---|---|---|---|---|
| 1 | 129.06586 | 65.03657 | 43.69347 | Q | | | | 22 |
| 2 | 266.12477 | 133.56602 | 89.37977 | H | 2226.03573 | 1113.52150 | 742.68343 | 21 |
| 3 | 379.20884 | 190.10806 | 127.07446 | I | 2088.97682 | 1044.99205 | 696.99712 | 20 |
| 4 | 508.25144 | 254.62936 | 170.08866 | E | 1975.89275 | 988.45001 | 659.30243 | 19 |
| 5 | 595.28347 | 298.14537 | 199.09934 | S | 1846.85015 | 923.92871 | 616.28823 | 18 |
| 6 | 732.34238 | 366.67483 | 244.78564 | H | 1759.81812 | 880.41270 | 587.27756 | 17 |
| 7 | 879.41080 | 440.20904 | 293.80845 | F | 1622.75921 | 811.88324 | 541.59125 | 16 |
| 8 | 966.44283 | 483.72505 | 322.81913 | S | 1475.69079 | 738.34903 | 492.56845 | 15 |
| 9 | 1067.49051 | 534.24889 | 356.50169 | T | 1388.65876 | 694.83302 | 463.55777 | 14 |
| 10 | 1124.51198 | 562.75963 | 375.50884 | G | 1287.61108 | 644.30918 | 429.87521 | 13 |
| 11 | 1221.56475 | 611.28601 | 407.85977 | P | 1230.58961 | 615.79844 | 410.86805 | 12 |
| 12 | 1322.61243 | 661.80985 | 441.54233 | T | 1133.53684 | 567.27206 | 378.51713 | 11 |
| 13 | 1421.68085 | 711.34406 | 474.56513 | V | 1032.48916 | 516.74822 | 344.83457 | 10 |
| 14 | 1520.74927 | 760.87827 | 507.58794 | V | 933.42074 | 467.21401 | 311.81176 | 9 |
| 15 | 1657.80818 | 829.40773 | 553.27424 | H | 834.35232 | 417.67980 | 278.78896 | 8 |
| 16 | 1808.80211 | 904.90469 | 603.60555 | C-hyper | 697.29341 | 349.15034 | 233.10265 | 7 |
| 17 | 1895.83414 | 948.42071 | 632.61623 | S | 546.29947 | 273.65337 | 182.77134 | 6 |
| 18 | 1966.87126 | 983.93927 | 656.29527 | A | 459.26744 | 230.13736 | 153.76066 | 5 |
| 19 | 2023.89273 | 1012.45000 | 675.30243 | G | 388.23032 | 194.61880 | 130.08162 | 4 |
| 20 | 2122.96115 | 1061.98421 | 708.32523 | V | 331.20885 | 166.10806 | 111.07447 | 3 |
| 21 | 2179.98262 | 1090.49495 | 727.33239 | G | 232.14043 | 116.57385 | 78.05166 | 2 |
| 22 | | | | R | 175.11896 | 88.06312 | 59.04450 | 1 |

Fragment Spectrum

Extracted from: D:\wu000024\20161117_Fin05_3reps\Set2\20161118_OR12_A6_WW_Fin05_10_1.raw #4674 RT: 16.03
FTMS, HCD@27.00, z=+3, Mono m/z=785.36920 Da, MH+=2354.09305 Da, Match Tol.=0.05 Da

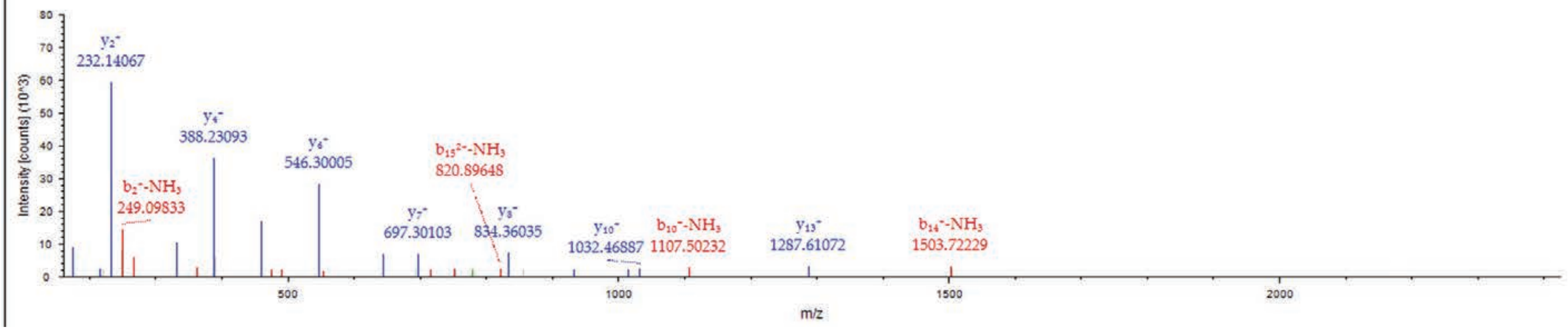

| Uniprot Accession | Description | Gene name | PTP type | Sequence | Hyperoxidised cysteine | Total MS/MS count |
|---|---|---|---|---|---|---|
| B6HY54 | Protein-tyrosine phosphatase | ptprja | Receptor | HSPTLVHcSAGVGR | C8(hyper) | 52 |

Peptide Summary

Sequence: HSPTLVHCSAGVGR, C8-hyperoxidation (47.98474 Da)
Charge: +2, Monoisotopic m/z: 734.85175 Da (+0.01 mmu/+0.01 ppm), MH+: 1468.69621 Da, RT: 14.24 min,
Identified with: Mascot (v1.30); IonScore:109, Exp Value:1.6E-009, Ions matched by search engine: 11/118
Fragment match tolerance used for search: 0.05 Da

Fragment Matches

Value Type: Theo. Mass [Da]

Ion Series | Neutral Losses | Precursor Ions

| #1 | b+ | b2+ | Seq. | y+ | y2+ | #2 |
|---|---|---|---|---|---|---|
| 1 | 138.06619 | 69.53673 | H | | | 14 |
| 2 | 225.09822 | 113.05275 | S | 1331.63729 | 666.32228 | 13 |
| 3 | 322.15099 | 161.57913 | P | 1244.60526 | 622.80627 | 12 |
| 4 | 423.19867 | 212.10297 | T | 1147.55249 | 574.27988 | 11 |
| 5 | 536.28274 | 268.64501 | L | 1046.50481 | 523.75604 | 10 |
| 6 | 635.35116 | 318.17922 | V | 933.42074 | 467.21401 | 9 |
| 7 | 772.41007 | 386.70867 | H | 834.35232 | 417.67980 | 8 |
| 8 | 923.40400 | 462.20564 | C-hyper | 697.29341 | 349.15034 | 7 |
| 9 | 1010.43603 | 505.72165 | S | 546.29947 | 273.65337 | 6 |
| 10 | 1081.47315 | 541.24021 | A | 459.26744 | 230.13736 | 5 |
| 11 | 1138.49462 | 569.75095 | G | 388.23032 | 194.61880 | 4 |
| 12 | 1237.56304 | 619.28516 | V | 331.20885 | 166.10806 | 3 |
| 13 | 1294.58451 | 647.79589 | G | 232.14043 | 116.57385 | 2 |
| 14 | | | R | 175.11896 | 88.06312 | 1 |

Fragment Spectrum

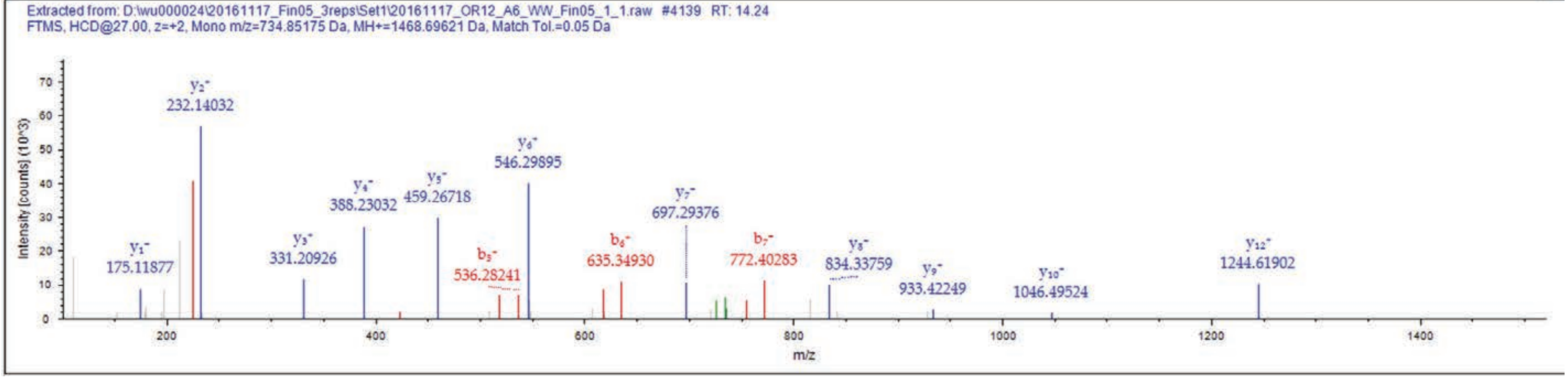

| Uniprot Accession | Description | Gene name | PTP type | Sequence | Hyperoxidised cysteine | Total MS/MS count |
|---|---|---|---|---|---|---|
| X1WCM2 | Uncharacterized protein (Fragment) | ptprjb.1 | Receptor | HSPTV**VHcSAGVGR** | C8(hyper) | 21 |

Peptide Summary

Sequence: HSPTVVHCSAGVGR, C8-hyperoxidation (47.98474 Da)
Charge: +2, Monoisotopic m/z: 727.84564 Da (+1.73 mmu/+2.38 ppm), MH+: 1454.68401 Da, RT: 12.80 min,
Identified with: Mascot (v1.30); IonScore:116, Exp Value:2.6E-010, Ions matched by search engine: 12/118
Fragment match tolerance used for search: 0.05 Da

Fragment Matches

Value Type: Theo. Mass [Da]

Ion Series | Neutral Losses | Precursor Ions

| #1 | $b^+$ | $b^{2+}$ | Seq. | $y^+$ | $y^{2+}$ | #2 |
|---|---|---|---|---|---|---|
| 1 | 138.06619 | 69.53673 | H | | | 14 |
| 2 | 225.09822 | 113.05275 | S | 1317.62164 | 659.31446 | 13 |
| 3 | 322.15099 | 161.57913 | P | 1230.58961 | 615.79844 | 12 |
| 4 | 423.19867 | 212.10297 | T | 1133.53684 | 567.27206 | 11 |
| 5 | 522.26709 | 261.63718 | V | 1032.48916 | 516.74822 | 10 |
| 6 | 621.33551 | 311.17139 | V | 933.42074 | 467.21401 | 9 |
| 7 | 758.39442 | 379.70085 | H | 834.35232 | 417.67980 | 8 |
| 8 | 909.38835 | 455.19781 | C-hyper | 697.29341 | 349.15034 | 7 |
| 9 | 996.42038 | 498.71383 | S | 546.29947 | 273.65337 | 6 |
| 10 | 1067.45750 | 534.23239 | A | 459.26744 | 230.13736 | 5 |
| 11 | 1124.47897 | 562.74312 | G | 388.23032 | 194.61880 | 4 |
| 12 | 1223.54739 | 612.27733 | V | 331.20885 | 166.10806 | 3 |
| 13 | 1280.56886 | 640.78807 | G | 232.14043 | 116.57385 | 2 |
| 14 | | | R | 175.11896 | 88.06312 | 1 |

Fragment Spectrum

Extracted from: D:\wu000024\20161117_Fin05_3reps\Set3\20161120_OR12_A6_WW_Fin05_16_1.raw #3516 RT: 12.80
FTMS, HCD@27.00, z=+2, Mono m/z=727.84564 Da, MH+=1454.68401 Da, Match Tol.=0.05 Da

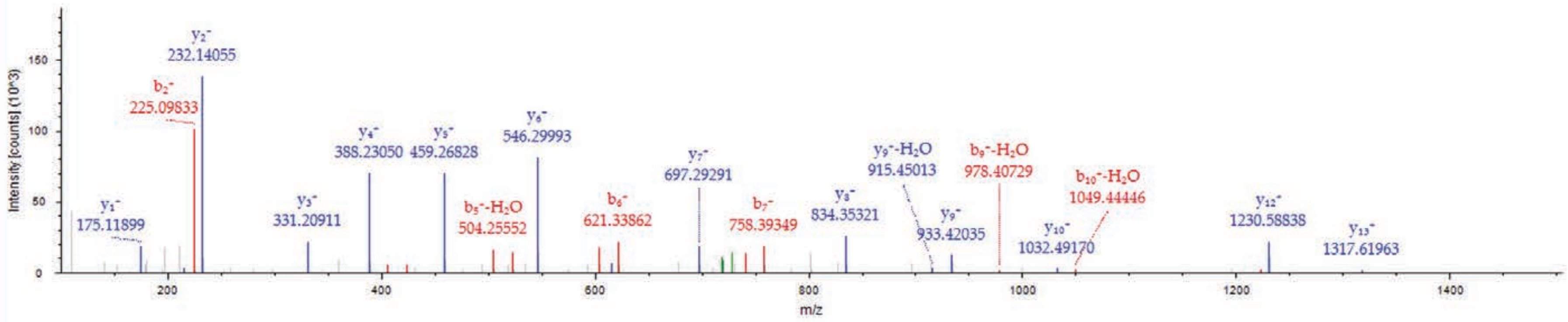

| Uniprot Accession | Description | Gene name | PTP type | Sequence | Hyperoxidised cysteine | Total MS/MS count |
|---|---|---|---|---|---|---|
| X1WF45 | Uncharacterized protein (Fragment) | ptprjb.2 | Receptor | EHMNQYScTSPTV**VHcSAGVGR** | C16(hyper) | 6 |

Peptide Summary

Sequence: EHMNQYSCTSPTVVHCSAGVGR, C8-Carbamidomethyl (57.02146 Da), C16-hyperoxidation (47.98474 Da)
Charge: +3, Monoisotopic m/z: 823.35107 Da (-0.06 mmu/-0.07 ppm), MH+: 2468.03867 Da, RT: 15.27 min,
Identified with: Mascot (v1.30); IonScore:83, Exp Value:3.0E-007, Ions matched by search engine: 16/236
Fragment match tolerance used for search: 0.05 Da

Fragment Matches

Value Type: Theo. Mass [Da]

Ion Series | Neutral Losses | Precursor Ions

| #1 | $b^+$ | $b^{2+}$ | $b^{3+}$ | Seq. | $y^+$ | $y^{2+}$ | $y^{3+}$ | #2 |
|---|---|---|---|---|---|---|---|---|
| 1 | 130.04988 | 65.52858 | 44.02148 | E | | | | 22 |
| 2 | 267.10879 | 134.05803 | 89.70778 | H | 2338.99624 | 1170.00176 | 780.33693 | 21 |
| 3 | 398.14929 | 199.57828 | 133.38795 | M | 2201.93733 | 1101.47230 | 734.65063 | 20 |
| 4 | 512.19222 | 256.59975 | 171.40226 | N | 2070.89683 | 1035.95205 | 690.97046 | 19 |
| 5 | 640.25080 | 320.62904 | 214.08845 | Q | 1956.85390 | 978.93059 | 652.95615 | 18 |
| 6 | 803.31412 | 402.16070 | 268.44289 | Y | 1828.79532 | 914.90130 | 610.26996 | 17 |
| 7 | 890.34615 | 445.67671 | 297.45357 | S | 1665.73200 | 833.36964 | 555.91552 | 16 |
| 8 | 1050.37680 | 525.69204 | 350.79712 | C-Carbami... | 1578.69997 | 789.85362 | 526.90484 | 15 |
| 9 | 1151.42448 | 576.21588 | 384.47968 | T | 1418.66932 | 709.83830 | 473.56129 | 14 |
| 10 | 1238.45651 | 619.73189 | 413.49035 | S | 1317.62164 | 659.31446 | 439.87873 | 13 |
| 11 | 1335.50928 | 668.25828 | 445.84128 | P | 1230.58961 | 615.79844 | 410.86805 | 12 |
| 12 | 1436.55696 | 718.78212 | 479.52384 | T | 1133.53684 | 567.27206 | 378.51713 | 11 |
| 13 | 1535.62538 | 768.31633 | 512.54664 | V | 1032.48916 | 516.74822 | 344.83457 | 10 |
| 14 | 1634.69380 | 817.85054 | 545.56945 | V | 933.42074 | 467.21401 | 311.81176 | 9 |
| 15 | 1771.75271 | 886.37999 | 591.25575 | H | 834.35232 | 417.67980 | 278.78896 | 8 |
| 16 | 1922.74664 | 961.87696 | 641.58707 | C-hyper | 697.29341 | 349.15034 | 233.10265 | 7 |
| 17 | 2009.77867 | 1005.39298 | 670.59774 | S | 546.29947 | 273.65337 | 182.77134 | 6 |
| 18 | 2080.81579 | 1040.91154 | 694.27678 | A | 459.26744 | 230.13736 | 153.76066 | 5 |
| 19 | 2137.83726 | 1069.42227 | 713.28394 | G | 388.23032 | 194.61880 | 130.08162 | 4 |
| 20 | 2236.90568 | 1118.95648 | 746.30675 | V | 331.20885 | 166.10806 | 111.07447 | 3 |
| 21 | 2293.92715 | 1147.46722 | 765.31390 | G | 232.14043 | 116.57385 | 78.05166 | 2 |
| 22 | | | | R | 175.11896 | 88.06312 | 59.04450 | 1 |

Fragment Spectrum

Extracted from: D:\wu000024\20161117_Fin05_3reps\Set2\20161118_OR12_A6_WW_Fin05_7_1.raw #4591 RT: 15.27
FTMS, HCD@27.00, z=+3, Mono m/z=823.35107 Da, MH+=2468.03867 Da, Match Tol.=0.05 Da

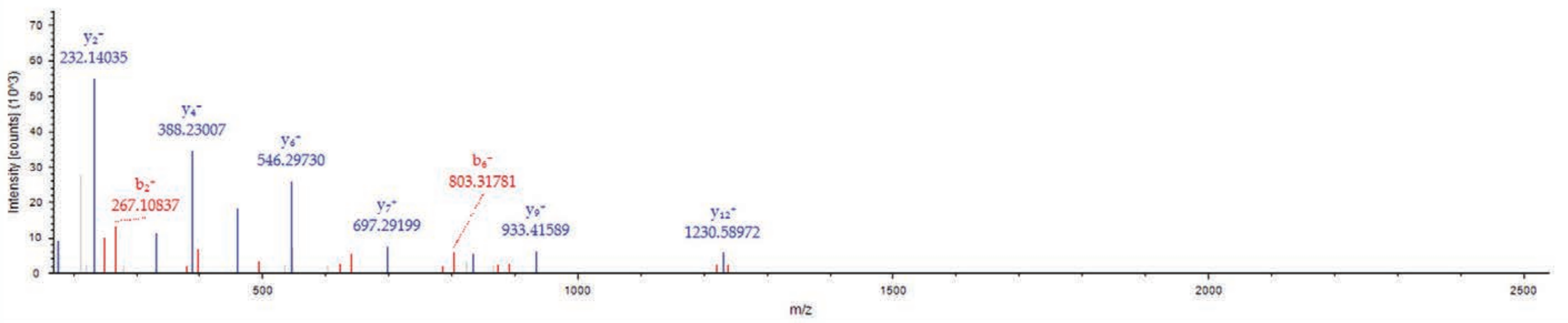

| Uniprot Accession | Description | Gene name | PTP type | Sequence | Hyperoxidised cysteine | Total MS/MS count |
|---|---|---|---|---|---|---|
| X1WD65 | Uncharacterized protein | ptprk | Receptor | MSNPPTAGPIV**VHcSAGAGR** | C14(hyper) | 90 |

**Peptide Summary**

Sequence: MSNPPTAGPIVVHCSAGAGR, C14-hyperoxidation (47.98474 Da)
Charge: +2, Monoisotopic m/z: 985.46545 Da (+0.83 mmu/+0.85 ppm), MH+: 1969.92363 Da, RT: 17.46 min,
Identified with: Mascot (v1.30); IonScore:124, Exp Value:5.1E-011, Ions matched by search engine: 16/212
Fragment match tolerance used for search: 0.05 Da

**Fragment Matches**

Value Type: Theo. Mass [Da]

Ion Series | Neutral Losses | Precursor Ions

| #1 | $b^+$ | $b^{2+}$ | Seq. | $y^+$ | $y^{2+}$ | #2 |
|---|---|---|---|---|---|---|
| 1 | 132.04778 | 66.52753 | M | | | 20 |
| 2 | 219.07981 | 110.04354 | S | 1838.88147 | 919.94437 | 19 |
| 3 | 333.12274 | 167.06501 | N | 1751.84944 | 876.42836 | 18 |
| 4 | 430.17551 | 215.59139 | P | 1637.80651 | 819.40689 | 17 |
| 5 | 527.22828 | 264.11778 | P | 1540.75374 | 770.88051 | 16 |
| 6 | 628.27596 | 314.64162 | T | 1443.70097 | 722.35412 | 15 |
| 7 | 699.31308 | 350.16018 | A | 1342.65329 | 671.83028 | 14 |
| 8 | 756.33455 | 378.67091 | G | 1271.61617 | 636.31172 | 13 |
| 9 | 853.38732 | 427.19730 | P | 1214.59470 | 607.80099 | 12 |
| 10 | 966.47139 | 483.73933 | I | 1117.54193 | 559.27460 | 11 |
| 11 | 1065.53981 | 533.27354 | V | 1004.45786 | 502.73257 | 10 |
| 12 | 1164.60823 | 582.80775 | V | 905.38944 | 453.19836 | 9 |
| 13 | 1301.66714 | 651.33721 | H | 806.32102 | 403.66415 | 8 |
| 14 | 1452.66107 | 726.83417 | C-hyper | 669.26211 | 335.13469 | 7 |
| 15 | 1539.69310 | 770.35019 | S | 518.26817 | 259.63772 | 6 |
| 16 | 1610.73022 | 805.86875 | A | 431.23614 | 216.12171 | 5 |
| 17 | 1667.75169 | 834.37948 | G | 360.19902 | 180.60315 | 4 |
| 18 | 1738.78881 | 869.89804 | A | 303.17755 | 152.09241 | 3 |
| 19 | 1795.81028 | 898.40878 | G | 232.14043 | 116.57385 | 2 |
| 20 | | | R | 175.11896 | 88.06312 | 1 |

**Fragment Spectrum**

Extracted from: D:\wu000024\20161117_Fin05_3reps\Set1\20161117_OR12_A6_WW_Fin05_1_5.raw #5230 RT: 17.46
FTMS, HCD@27.00, z=+2, Mono m/z=985.46545 Da, MH+=1969.92363 Da, Match Tol.=0.05 Da

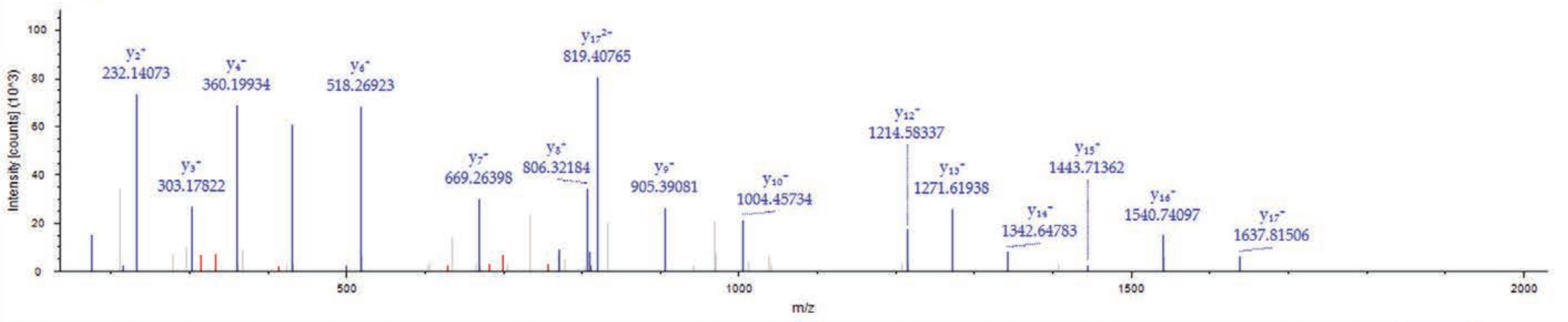

| Uniprot Accession | Description | Gene name | PTP type | Sequence | Hyperoxidised cysteine | Total MS/MS count |
|---|---|---|---|---|---|---|
| A0A0R4IA95 | Uncharacterized protein (Fragment) | ptpro | Receptor | GPIV**VHcSAGVGR** | C7(hyper) | 52 |

Peptide Summary

Sequence: GPIVVHCSAGVGR, C7-hyperoxidation (47.98474 Da)
Charge: +2, Monoisotopic m/z: 650.32776 Da (+0.39 mmu/+0.6 ppm), MH+: 1299.64824 Da, RT: 15.37 min,
Identified with: Mascot (v1.30); IonScore:116, Exp Value:3.1E-010, Ions matched by search engine: 11/96
Fragment match tolerance used for search: 0.05 Da

Fragment Matches

Value Type: Theo. Mass [Da]

Ion Series | Neutral Losses | Precursor Ions

| #1 | b+ | b2+ | Seq. | y+ | y2+ | #2 |
|---|---|---|---|---|---|---|
| 1 | 58.02875 | 29.51801 | G | | | 13 |
| 2 | 155.08152 | 78.04440 | P | 1242.62600 | 621.81664 | 12 |
| 3 | 268.16559 | 134.58643 | I | 1145.57323 | 573.29025 | 11 |
| 4 | 367.23401 | 184.12064 | V | 1032.48916 | 516.74822 | 10 |
| 5 | 466.30243 | 233.65485 | V | 933.42074 | 467.21401 | 9 |
| 6 | 603.36134 | 302.18431 | H | 834.35232 | 417.67980 | 8 |
| 7 | 754.35527 | 377.68127 | C-hyper | 697.29341 | 349.15034 | 7 |
| 8 | 841.38730 | 421.19729 | S | 546.29947 | 273.65337 | 6 |
| 9 | 912.42442 | 456.71585 | A | 459.26744 | 230.13736 | 5 |
| 10 | 969.44589 | 485.22658 | G | 388.23032 | 194.61880 | 4 |
| 11 | 1068.51431 | 534.76079 | V | 331.20885 | 166.10806 | 3 |
| 12 | 1125.53578 | 563.27153 | G | 232.14043 | 116.57385 | 2 |
| 13 | | | R | 175.11896 | 88.06312 | 1 |

Fragment Spectrum

Extracted from: D:\wu000024\20161117_Fin05_3reps\Set3\20161120_OR12_A6_WW_Fin05_13_3.raw #4911 RT: 15.37
FTMS, HCD@27.00, z=+2, Mono m/z=650.32776 Da, MH+=1299.64824 Da, Match Tol.=0.05 Da

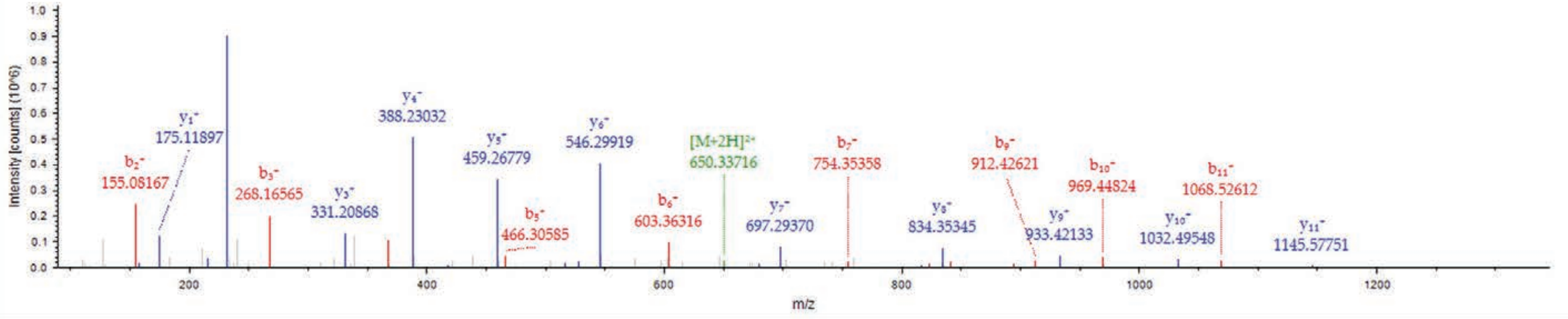

| Uniprot Accession | Description | Gene name | PTP type | Sequence | Hyperoxidised cysteine | Total MS/MS count |
|---|---|---|---|---|---|---|
| Q90YJ4_site1 | Receptor protein-tyrosine phosphatase sigma (Fragment) | ptprsa | Receptor | EQFGQDGPISVHcSAGVGR | C13(hyper) | 21 |

Peptide Summary

Sequence: EQFGQDGPISVHCSAGVGR, C13-hyperoxidation (47.98474 Da)
Charge: +2, Monoisotopic m/z: 996.44788 Da (+0.4 mmu/+0.4 ppm), MH+: 1991.88848 Da, RT: 18.74 min,
Identified with: Mascot (v1.30); IonScore:134, Exp Value:3.7E-012, Ions matched by search engine: 15/204
Fragment match tolerance used for search: 0.05 Da

Fragment Matches

Value Type: Theo. Mass [Da]

Ion Series | Neutral Losses | Precursor Ions

| #1 | b+ | b2+ | Seq. | y+ | y2+ | #2 |
|---|---|---|---|---|---|---|
| 1 | 130.04988 | 65.52858 | E | | | 19 |
| 2 | 258.10846 | 129.55787 | Q | 1862.84508 | 931.92618 | 18 |
| 3 | 405.17688 | 203.09208 | F | 1734.78650 | 867.89689 | 17 |
| 4 | 462.19835 | 231.60281 | G | 1587.71808 | 794.36268 | 16 |
| 5 | 590.25693 | 295.63210 | Q | 1530.69661 | 765.85194 | 15 |
| 6 | 705.28388 | 353.14558 | D | 1402.63803 | 701.82265 | 14 |
| 7 | 762.30535 | 381.65631 | G | 1287.61108 | 644.30918 | 13 |
| 8 | 859.35812 | 430.18270 | P | 1230.58961 | 615.79844 | 12 |
| 9 | 972.44219 | 486.72473 | I | 1133.53684 | 567.27206 | 11 |
| 10 | 1059.47422 | 530.24075 | S | 1020.45277 | 510.73002 | 10 |
| 11 | 1158.54264 | 579.77496 | V | 933.42074 | 467.21401 | 9 |
| 12 | 1295.60155 | 648.30441 | H | 834.35232 | 417.67980 | 8 |
| 13 | 1446.59548 | 723.80138 | C-hyper | 697.29341 | 349.15034 | 7 |
| 14 | 1533.62751 | 767.31739 | S | 546.29947 | 273.65337 | 6 |
| 15 | 1604.66463 | 802.83595 | A | 459.26744 | 230.13736 | 5 |
| 16 | 1661.68610 | 831.34669 | G | 388.23032 | 194.61880 | 4 |
| 17 | 1760.75452 | 880.88090 | V | 331.20885 | 166.10806 | 3 |
| 18 | 1817.77599 | 909.39163 | G | 232.14043 | 116.57385 | 2 |
| 19 | | | R | 175.11896 | 88.06312 | 1 |

Fragment Spectrum

Extracted from: D:\wu000024\20161117_Fin05_3reps\Set3\20161120_OR12_A6_WW_Fin05_13_1.raw #5908 RT: 18.74
FTMS, HCD@27.00, z=+2, Mono m/z=996.44788 Da, MH+=1991.88848 Da, Match Tol.=0.05 Da

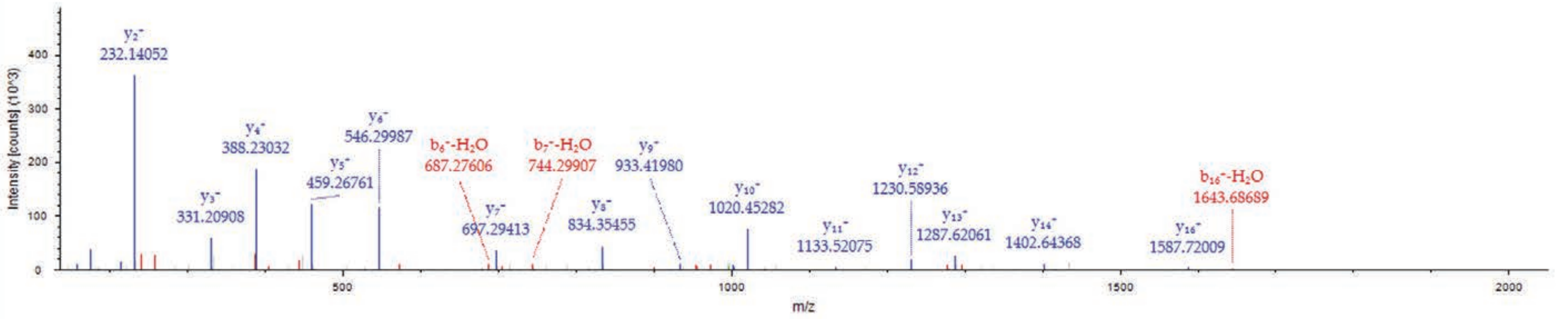

| Uniprot Accession | Description | Gene name | PTP type | Sequence | Hyperoxidised cysteine | Total MS/MS count |
|---|---|---|---|---|---|---|
| Q90YJ4_site2 | Receptor protein-tyrosine phosphatase sigma (Fragment) | ptprsa | Receptor | TcNPPDAGPIIAHcSAGVGR | C14(hyper) | 45 |

Peptide Summary

Sequence: TCNPPDAGPIIAHCSAGVGR, C2-Carbamidomethyl (57.02146 Da), C14-hyperoxidation (47.98474 Da)
Charge: +2, Monoisotopic m/z: 1020.96613 Da (+1.14 mmu/+1.12 ppm), MH+: 2040.92497 Da, RT: 17.33 min,
Identified with: Mascot (v1.30); IonScore:123, Exp Value:6.1E-011, Ions matched by search engine: 15/214
Fragment match tolerance used for search: 0.05 Da

Fragment Matches

Value Type: Theo. Mass [Da]

Ion Series | Neutral Losses | Precursor Ions

| #1 | b+ | b2+ | Seq. | y+ | y2+ | #2 |
|---|---|---|---|---|---|---|
| 1 | 102.05496 | 51.53112 | T | | | 20 |
| 2 | 262.08561 | 131.54644 | C-Carbami... | 1939.87501 | 970.44114 | 19 |
| 3 | 376.12854 | 188.56791 | N | 1779.84436 | 890.42582 | 18 |
| 4 | 473.18131 | 237.09429 | P | 1665.80143 | 833.40435 | 17 |
| 5 | 570.23408 | 285.62068 | P | 1568.74866 | 784.87797 | 16 |
| 6 | 685.26103 | 343.13415 | D | 1471.69589 | 736.35158 | 15 |
| 7 | 756.29815 | 378.65271 | A | 1356.66894 | 678.83811 | 14 |
| 8 | 813.31962 | 407.16345 | G | 1285.63182 | 643.31955 | 13 |
| 9 | 910.37239 | 455.68983 | P | 1228.61035 | 614.80881 | 12 |
| 10 | 1023.45646 | 512.23187 | I | 1131.55758 | 566.28243 | 11 |
| 11 | 1136.54053 | 568.77390 | I | 1018.47351 | 509.74039 | 10 |
| 12 | 1207.57765 | 604.29246 | A | 905.38944 | 453.19836 | 9 |
| 13 | 1344.63656 | 672.82192 | H | 834.35232 | 417.67980 | 8 |
| 14 | 1495.63049 | 748.31889 | C-hyper | 697.29341 | 349.15034 | 7 |
| 15 | 1582.66252 | 791.83490 | S | 546.29947 | 273.65337 | 6 |
| 16 | 1653.69964 | 827.35346 | A | 459.26744 | 230.13736 | 5 |
| 17 | 1710.72111 | 855.86420 | G | 388.23032 | 194.61880 | 4 |
| 18 | 1809.78953 | 905.39841 | V | 331.20885 | 166.10806 | 3 |
| 19 | 1866.81100 | 933.90914 | G | 232.14043 | 116.57385 | 2 |
| 20 | | | R | 175.11896 | 88.06312 | 1 |

Fragment Spectrum

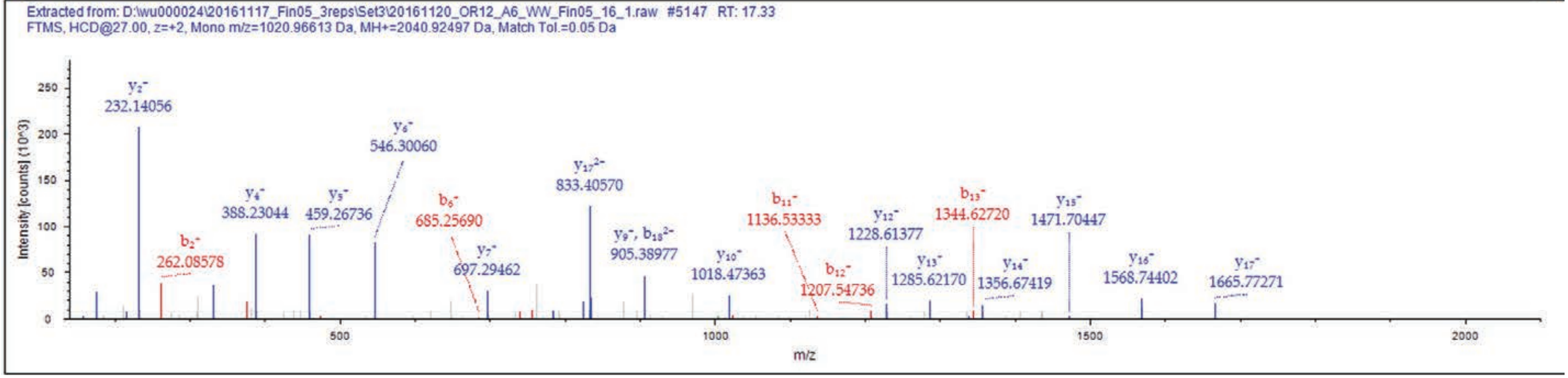

| Uniprot Accession | Description | Gene name | PTP type | Sequence | Hyperoxidised cysteine | Total MS/MS count |
|---|---|---|---|---|---|---|
| A0A0R4ITF5 (C7U125) | Uncharacterized protein | ptprub | Receptor | TSTPLDAGPVVVHcSVGAGR | C14(hyper) | 13 |

Peptide Summary

Sequence: TSTPLDAGPVVVHCSVGAGR, C14-hyperoxidation (47.98474 Da)
Charge: +2, Monoisotopic m/z: 985.98657 Da (+2.88 mmu/+2.92 ppm), MH+: 1970.96587 Da, RT: 19.32 min,
Identified with: Mascot (v1.30); IonScore:103, Exp Value:7.9E-009, Ions matched by search engine: 11/180
Fragment match tolerance used for search: 0.05 Da

Fragment Matches

Value Type: Theo. Mass [Da]

Ion Series | Neutral Losses | Precursor Ions

| #1 | $b^{+}$ | $b^{2+}$ | Seq. | $y^{+}$ | $y^{2+}$ | #2 |
|---|---|---|---|---|---|---|
| 1 | 102.05496 | 51.53112 | T | | | 20 |
| 2 | 189.08699 | 95.04713 | S | 1869.91244 | 935.45986 | 19 |
| 3 | 290.13467 | 145.57097 | T | 1782.88041 | 891.94384 | 18 |
| 4 | 387.18744 | 194.09736 | P | 1681.83273 | 841.42000 | 17 |
| 5 | 500.27151 | 250.63939 | L | 1584.77996 | 792.89362 | 16 |
| 6 | 615.29846 | 308.15287 | D | 1471.69589 | 736.35158 | 15 |
| 7 | 686.33558 | 343.67143 | A | 1356.66894 | 678.83811 | 14 |
| 8 | 743.35705 | 372.18216 | G | 1285.63182 | 643.31955 | 13 |
| 9 | 840.40982 | 420.70855 | P | 1228.61035 | 614.80881 | 12 |
| 10 | 939.47824 | 470.24276 | V | 1131.55758 | 566.28243 | 11 |
| 11 | 1038.54666 | 519.77697 | V | 1032.48916 | 516.74822 | 10 |
| 12 | 1137.61508 | 569.31118 | V | 933.42074 | 467.21401 | 9 |
| 13 | 1274.67399 | 637.84063 | H | 834.35232 | 417.67980 | 8 |
| 14 | 1425.66792 | 713.33760 | C-hyper | 697.29341 | 349.15034 | 7 |
| 15 | 1512.69995 | 756.85361 | S | 546.29947 | 273.65337 | 6 |
| 16 | 1611.76837 | 806.38782 | V | 459.26744 | 230.13736 | 5 |
| 17 | 1668.78984 | 834.89856 | G | 360.19902 | 180.60315 | 4 |
| 18 | 1739.82696 | 870.41712 | A | 303.17755 | 152.09241 | 3 |
| 19 | 1796.84843 | 898.92785 | G | 232.14043 | 116.57385 | 2 |
| 20 | | | R | 175.11896 | 88.06312 | 1 |

Fragment Spectrum

Extracted from: D:\wu000024\20161117_Fin05_3reps\Set3\20161120_OR12_A6_WW_Fin05_16_1.raw #5920 RT: 19.32
FTMS, HCD@27.00, z=+2, Mono m/z=985.98657 Da, MH+=1970.96587 Da, Match Tol.=0.05 Da

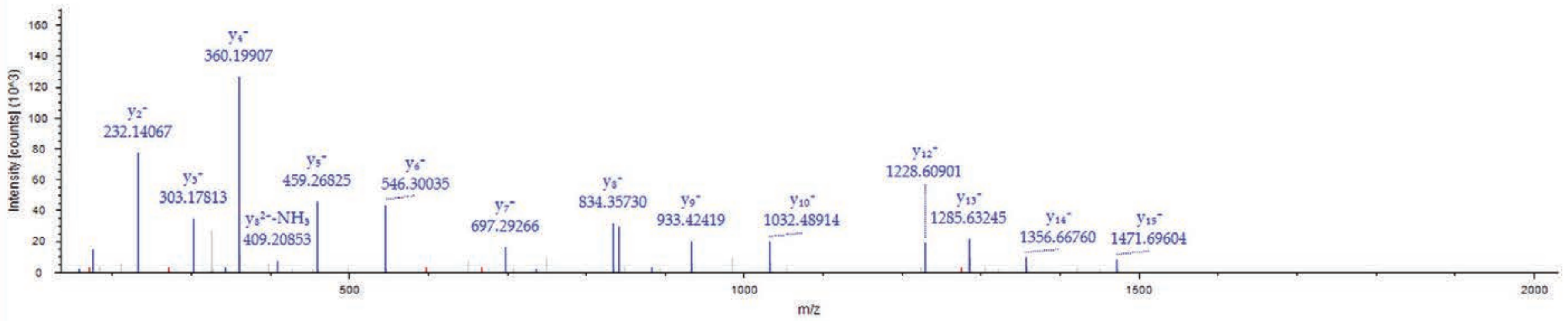

| Uniprot Accession | Description | Gene name | PTP type | Sequence | Hyperoxidised cysteine | Total MS/MS count |
|---|---|---|---|---|---|---|
| X1WET2 | Uncharacterized protein (Fragment) | ptprz1a | Receptor | AQTDGMGPMV**VHcSAGVGR** | C13(hyper) | 16 |

**Peptide Summary**

Sequence: AQTDGMGPMVVHCSAGVGR, M9-Oxidation (15.99492 Da), C13-hyperoxidation (47.98474 Da)
Charge: +2, Monoisotopic m/z: 968.92151 Da (+2.32 mmu/+2.39 ppm), MH+: 1936.83574 Da, RT: 16.47 min,
Identified with: Mascot (v1.30); IonScore:125, Exp Value:2.4E-011, Ions matched by search engine: 13/200
Fragment match tolerance used for search: 0.05 Da

**Fragment Matches**

Value Type: Theo. Mass [Da]

Ion Series | Neutral Losses | Precursor Ions

| #1 | $b^+$ | $b^{2+}$ | Seq. | $y^+$ | $y^{2+}$ | #2 |
|---|---|---|---|---|---|---|
| 1 | 72.04440 | 36.52584 | A | | | 19 |
| 2 | 200.10298 | 100.55513 | Q | 1865.79399 | 933.40063 | 18 |
| 3 | 301.15066 | 151.07897 | T | 1737.73541 | 869.37134 | 17 |
| 4 | 416.17761 | 208.59244 | D | 1636.68773 | 818.84750 | 16 |
| 5 | 473.19908 | 237.10318 | G | 1521.66078 | 761.33403 | 15 |
| 6 | 604.23958 | 302.62343 | M | 1464.63931 | 732.82329 | 14 |
| 7 | 661.26105 | 331.13416 | G | 1333.59881 | 667.30304 | 13 |
| 8 | 758.31382 | 379.66055 | P | 1276.57734 | 638.79231 | 12 |
| 9 | 905.34923 | 453.17825 | M-Oxidation | 1179.52457 | 590.26592 | 11 |
| 10 | 1004.41765 | 502.71246 | V | 1032.48916 | 516.74822 | 10 |
| 11 | 1103.48607 | 552.24667 | V | 933.42074 | 467.21401 | 9 |
| 12 | 1240.54498 | 620.77613 | H | 834.35232 | 417.67980 | 8 |
| 13 | 1391.53892 | 696.27310 | C-hyper | 697.29341 | 349.15034 | 7 |
| 14 | 1478.57095 | 739.78911 | S | 546.29947 | 273.65337 | 6 |
| 15 | 1549.60807 | 775.30767 | A | 459.26744 | 230.13736 | 5 |
| 16 | 1606.62954 | 803.81841 | G | 388.23032 | 194.61880 | 4 |
| 17 | 1705.69796 | 853.35262 | V | 331.20885 | 166.10806 | 3 |
| 18 | 1762.71943 | 881.86335 | G | 232.14043 | 116.57385 | 2 |
| 19 | | | R | 175.11896 | 88.06312 | 1 |

**Fragment Spectrum**

Extracted from: D:\wu000024\20161117_Fin05_3reps\Set1\20161117_OR12_A6_WW_Fin05_1_1.raw #4946 RT: 16.47
FTMS, HCD@27.00, z=+2, Mono m/z=968.92151 Da, MH+=1936.83574 Da, Match Tol.=0.05 Da

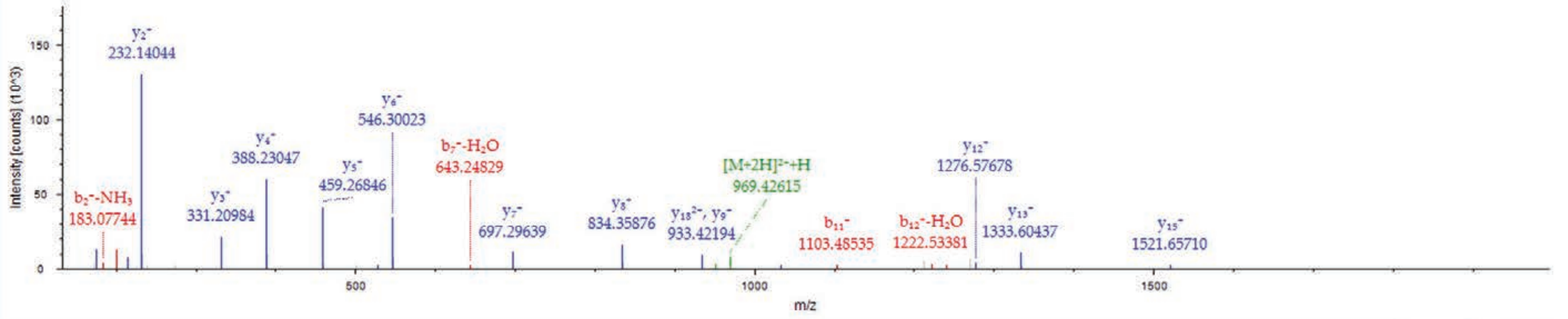

| Uniprot Accession | Description | Gene name | PTP type | Sequence | Hyperoxidised cysteine | Total MS/MS count |
|---|---|---|---|---|---|---|
| B8A5J4 | Uncharacterized protein | ptprz1b | Receptor | TAEMGPVV**VHcSAGVGR** | C11(hyper) | 104 |

Peptide Summary

Sequence: TAEMGPVVVHCSAGVGR, C11-hyperoxidation (47.98474 Da)
Charge: +2, Monoisotopic m/z: 859.40491 Da (+1.41 mmu/+1.64 ppm), MH+: 1717.80254 Da, RT: 17.90 min,
Identified with: Mascot (v1.30); IonScore:105, Exp Value:3.8E-009, Ions matched by search engine: 12/150
Fragment match tolerance used for search: 0.05 Da

Fragment Matches

Value Type: Theo. Mass [Da]

Ion Series | Neutral Losses | Precursor Ions

| #1 | $b^+$ | $b^{2+}$ | Seq. | $y^+$ | $y^{2+}$ | #2 |
|---|---|---|---|---|---|---|
| 1 | 102.05496 | 51.53112 | T | | | 17 |
| 2 | 173.09208 | 87.04968 | A | 1616.75204 | 808.87966 | 16 |
| 3 | 302.13468 | 151.57098 | E | 1545.71492 | 773.36110 | 15 |
| 4 | 433.17518 | 217.09123 | M | 1416.67232 | 708.83980 | 14 |
| 5 | 490.19665 | 245.60196 | G | 1285.63182 | 643.31955 | 13 |
| 6 | 587.24942 | 294.12835 | P | 1228.61035 | 614.80881 | 12 |
| 7 | 686.31784 | 343.66256 | V | 1131.55758 | 566.28243 | 11 |
| 8 | 785.38626 | 393.19677 | V | 1032.48916 | 516.74822 | 10 |
| 9 | 884.45468 | 442.73098 | V | 933.42074 | 467.21401 | 9 |
| 10 | 1021.51359 | 511.26043 | H | 834.35232 | 417.67980 | 8 |
| 11 | 1172.50752 | 586.75740 | C-hyper | 697.29341 | 349.15034 | 7 |
| 12 | 1259.53955 | 630.27341 | S | 546.29947 | 273.65337 | 6 |
| 13 | 1330.57667 | 665.79197 | A | 459.26744 | 230.13736 | 5 |
| 14 | 1387.59814 | 694.30271 | G | 388.23032 | 194.61880 | 4 |
| 15 | 1486.66656 | 743.83692 | V | 331.20885 | 166.10806 | 3 |
| 16 | 1543.68803 | 772.34765 | G | 232.14043 | 116.57385 | 2 |
| 17 | | | R | 175.11896 | 88.06312 | 1 |

Fragment Spectrum

Extracted from: D:\wu000024\20161117_Fin05_3reps\Set3\20161120_OR12_A6_WW_Fin05_13_1.raw #5586 RT: 17.90
FTMS, HCD@27.00, z=+2, Mono m/z=859.40491 Da, MH+=1717.80254 Da, Match Tol.=0.05 Da

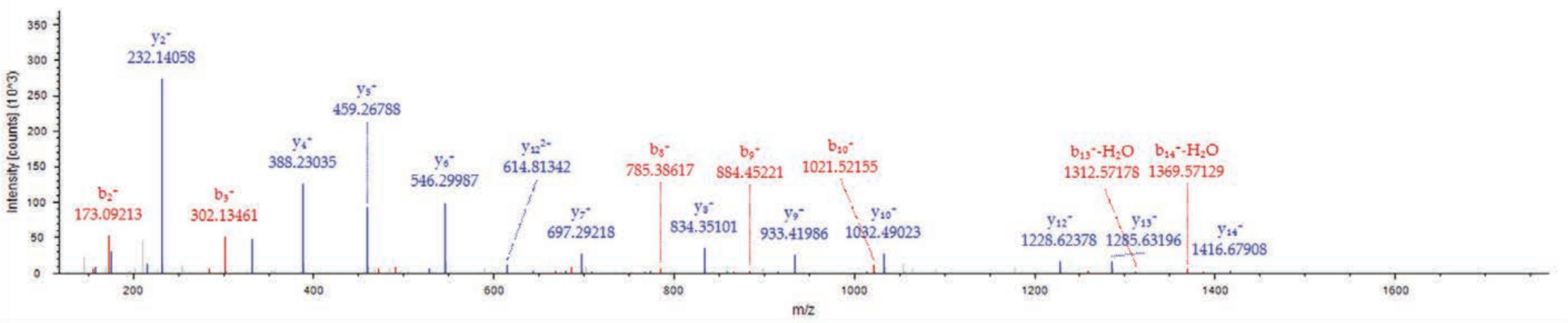

| Uniprot Accession | Description | Gene name | PTP type | Sequence | Hyperoxidised cysteine | Total MS/MS count |
|---|---|---|---|---|---|---|
| C7U142 | Protein-tyrosine phosphatase 1B (Fragment) | ptpn1 | Non-receptor | ESGcLSPELGPVVVHcSAGIGR | C16(hyper) | 142 |

Peptide Summary

Sequence: ESGCLSPELGPVVVHCSAGIGR, C4-hyperoxidation (47.98474 Da), C16-hyperoxidation (47.98474 Da)
Charge: +2, Monoisotopic m/z: 1132.02173 Da (+1.57 mmu/+1.39 ppm), MH+: 2263.03618 Da, RT: 22.55 min,
Identified with: Mascot (v1.30); IonScore:126, Exp Value:3.4E-011, Ions matched by search engine: 13/200
Fragment match tolerance used for search: 0.05 Da

Fragment Matches

Value Type: Theo. Mass [Da]

Ion Series | Neutral Losses | Precursor Ions

| #1 | b+ | b2+ | Seq. | y+ | y2+ | #2 |
|---|---|---|---|---|---|---|
| 1 | 130.04988 | 65.52858 | E | | | 22 |
| 2 | 217.08191 | 109.04459 | S | 2133.99044 | 1067.49886 | 21 |
| 3 | 274.10338 | 137.55533 | G | 2046.95841 | 1023.98284 | 20 |
| 4 | 425.09731 | 213.05229 | C-hyper | 1989.93694 | 995.47211 | 19 |
| 5 | 538.18138 | 269.59433 | L | 1838.94301 | 919.97514 | 18 |
| 6 | 625.21341 | 313.11034 | S | 1725.85894 | 863.43311 | 17 |
| 7 | 722.26618 | 361.63673 | P | 1638.82691 | 819.91709 | 16 |
| 8 | 851.30878 | 426.15803 | E | 1541.77414 | 771.39071 | 15 |
| 9 | 964.39285 | 482.70006 | L | 1412.73154 | 706.86941 | 14 |
| 10 | 1021.41432 | 511.21080 | G | 1299.64747 | 650.32737 | 13 |
| 11 | 1118.46709 | 559.73718 | P | 1242.62600 | 621.81664 | 12 |
| 12 | 1217.53551 | 609.27139 | V | 1145.57323 | 573.29025 | 11 |
| 13 | 1316.60393 | 658.80560 | V | 1046.50481 | 523.75604 | 10 |
| 14 | 1415.67235 | 708.33981 | V | 947.43639 | 474.22183 | 9 |
| 15 | 1552.73126 | 776.86927 | H | 848.36797 | 424.68762 | 8 |
| 16 | 1703.72519 | 852.36624 | C-hyper | 711.30906 | 356.15817 | 7 |
| 17 | 1790.75722 | 895.88225 | S | 560.31512 | 280.66120 | 6 |
| 18 | 1861.79434 | 931.40081 | A | 473.28309 | 237.14518 | 5 |
| 19 | 1918.81581 | 959.91155 | G | 402.24597 | 201.62662 | 4 |
| 20 | 2031.89988 | 1016.45358 | I | 345.22450 | 173.11589 | 3 |
| 21 | 2088.92135 | 1044.96432 | G | 232.14043 | 116.57385 | 2 |
| 22 | | | R | 175.11896 | 88.06312 | 1 |

Fragment Spectrum

Extracted from: D:\wu000024\20161117_Fin05_3reps\Set2\20161118_OR12_A6_WW_Fin05_10_4.raw #7170 RT: 22.55
FTMS, HCD@27.00, z=+2, Mono m/z=1132.02173 Da, MH+=2263.03618 Da, Match Tol.=0.05 Da

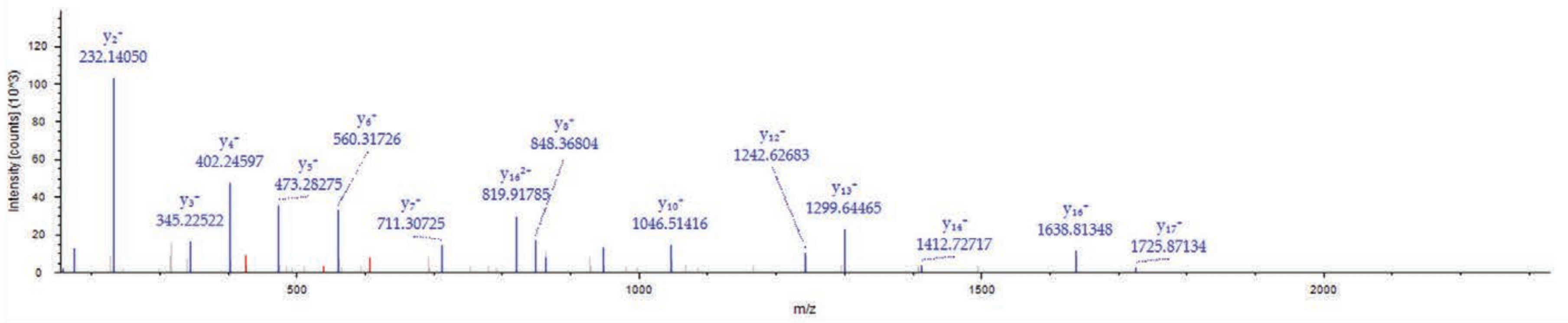

| Uniprot Accession | Description | Gene name | PTP type | Sequence | Hyperoxidised cysteine | Total MS/MS count |
|---|---|---|---|---|---|---|
| C7U151 | Protein-tyrosine phosphatase SHP-2a (Fragment) | ptpn11a | Non-receptor | QEGITGAGPIVVHcSAGIGR | C14(hyper) | 112 |

Peptide Summary

Sequence: QEGITGAGPIVVHCSAGIGR, C14-hyperoxidation (47.98474 Da)
Charge: +2, Monoisotopic m/z: 985.49371 Da (+2.02 mmu/+2.05 ppm), MH+: 1969.98015 Da, RT: 19.81 min,
Identified with: Mascot (v1.30); IonScore:146, Exp Value:3.8E-013, Ions matched by search engine: 14/216
Fragment match tolerance used for search: 0.05 Da

Fragment Matches

Value Type: Theo. Mass [Da]

Ion Series | Neutral Losses | Precursor Ions

| #1 | $b^+$ | $b^{2+}$ | Seq. | $y^+$ | $y^{2+}$ | #2 |
|---|---|---|---|---|---|---|
| 1 | 129.06586 | 65.03657 | Q | | | 20 |
| 2 | 258.10846 | 129.55787 | E | 1841.91753 | 921.46240 | 19 |
| 3 | 315.12993 | 158.06860 | G | 1712.87493 | 856.94110 | 18 |
| 4 | 428.21400 | 214.61064 | I | 1655.85346 | 828.43037 | 17 |
| 5 | 529.26168 | 265.13448 | T | 1542.76939 | 771.88833 | 16 |
| 6 | 586.28315 | 293.64521 | G | 1441.72171 | 721.36449 | 15 |
| 7 | 657.32027 | 329.16377 | A | 1384.70024 | 692.85376 | 14 |
| 8 | 714.34174 | 357.67451 | G | 1313.66312 | 657.33520 | 13 |
| 9 | 811.39451 | 406.20089 | P | 1256.64165 | 628.82446 | 12 |
| 10 | 924.47858 | 462.74293 | I | 1159.58888 | 580.29808 | 11 |
| 11 | 1023.54700 | 512.27714 | V | 1046.50481 | 523.75604 | 10 |
| 12 | 1122.61542 | 561.81135 | V | 947.43639 | 474.22183 | 9 |
| 13 | 1259.67433 | 630.34080 | H | 848.36797 | 424.68762 | 8 |
| 14 | 1410.66826 | 705.83777 | C-hyper | 711.30906 | 356.15817 | 7 |
| 15 | 1497.70029 | 749.35378 | S | 560.31512 | 280.66120 | 6 |
| 16 | 1568.73741 | 784.87234 | A | 473.28309 | 237.14518 | 5 |
| 17 | 1625.75888 | 813.38308 | G | 402.24597 | 201.62662 | 4 |
| 18 | 1738.84295 | 869.92511 | I | 345.22450 | 173.11589 | 3 |
| 19 | 1795.86442 | 898.43585 | G | 232.14043 | 116.57385 | 2 |
| 20 | | | R | 175.11896 | 88.06312 | 1 |

Fragment Spectrum

Extracted from: D:\wu000024\20161117_Fin05_3reps\Set2\20161118_OR12_A6_WW_Fin05_7_1.raw #6349 RT: 19.81
FTMS, HCD@27.00, z=+2, Mono m/z=985.49371 Da, MH+=1969.98015 Da, Match Tol.=0.05 Da

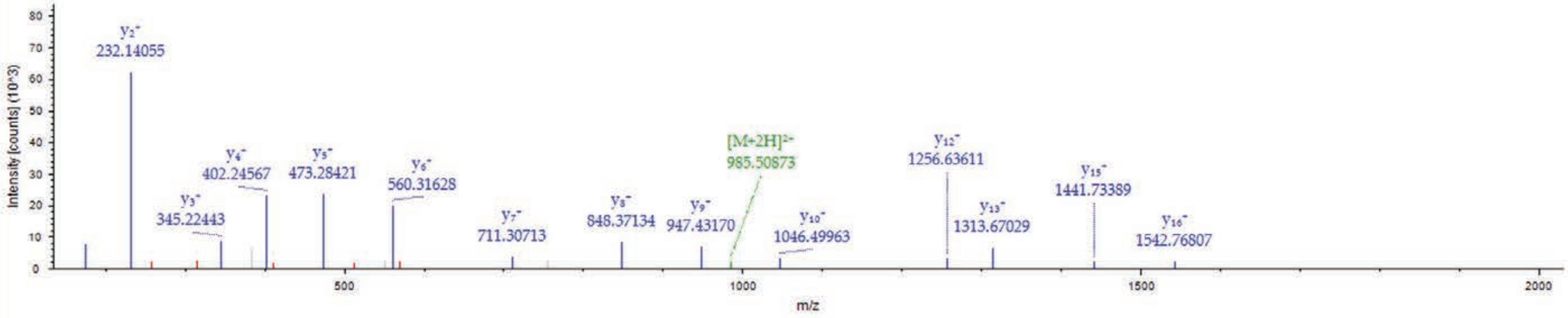

| Uniprot Accession | Description | Gene name | PTP type | Sequence | Hyperoxidised cysteine | Total MS/MS count |
|---|---|---|---|---|---|---|
| C7U120 | Protein-tyrosine phosphatase SHP-2b (Fragment) | ptpn11b | Non-receptor | TQSAIPESGPIV**VHcSAGIGR** | C15(hyper) | 59 |

Peptide Summary

Sequence: TQSAIPESGPIVVHCSAGIGR, C15-hyperoxidation (47.98474 Da)
Charge: +2, Monoisotopic m/z: 1064.03064 Da (+2 mmu/+1.88 ppm), MH+: 2127.05400 Da, RT: 19.62 min,
Identified with: Mascot (v1.30); IonScore:116, Exp Value:3.7E-010, Ions matched by search engine: 16/228
Fragment match tolerance used for search: 0.05 Da

Fragment Matches

Value Type: Theo. Mass [Da]

Ion Series | Neutral Losses | Precursor Ions

| #1 | $b^+$ | $b^{2+}$ | Seq. | $y^+$ | $y^{2+}$ | #2 |
|---|---|---|---|---|---|---|
| 1 | 102.05496 | 51.53112 | T | | | 21 |
| 2 | 230.11354 | 115.56041 | Q | 2026.00232 | 1013.50480 | 20 |
| 3 | 317.14557 | 159.07642 | S | 1897.94374 | 949.47551 | 19 |
| 4 | 388.18269 | 194.59498 | A | 1810.91171 | 905.95949 | 18 |
| 5 | 501.26676 | 251.13702 | I | 1739.87459 | 870.44093 | 17 |
| 6 | 598.31953 | 299.66340 | P | 1626.79052 | 813.89890 | 16 |
| 7 | 727.36213 | 364.18470 | E | 1529.73775 | 765.37251 | 15 |
| 8 | 814.39416 | 407.70072 | S | 1400.69515 | 700.85121 | 14 |
| 9 | 871.41563 | 436.21145 | G | 1313.66312 | 657.33520 | 13 |
| 10 | 968.46840 | 484.73784 | P | 1256.64165 | 628.82446 | 12 |
| 11 | 1081.55247 | 541.27987 | I | 1159.58888 | 580.29808 | 11 |
| 12 | 1180.62089 | 590.81408 | V | 1046.50481 | 523.75604 | 10 |
| 13 | 1279.68931 | 640.34829 | V | 947.43639 | 474.22183 | 9 |
| 14 | 1416.74822 | 708.87775 | H | 848.36797 | 424.68762 | 8 |
| 15 | 1567.74215 | 784.37471 | C-hyper | 711.30906 | 356.15817 | 7 |
| 16 | 1654.77418 | 827.89073 | S | 560.31512 | 280.66120 | 6 |
| 17 | 1725.81130 | 863.40929 | A | 473.28309 | 237.14518 | 5 |
| 18 | 1782.83277 | 891.92002 | G | 402.24597 | 201.62662 | 4 |
| 19 | 1895.91684 | 948.46206 | I | 345.22450 | 173.11589 | 3 |
| 20 | 1952.93831 | 976.97279 | G | 232.14043 | 116.57385 | 2 |
| 21 | | | R | 175.11896 | 88.06312 | 1 |

Fragment Spectrum

Extracted from: D:\wu000024\20161117_Fin05_3reps\Set3\20161120_OR12_A6_WW_Fin05_16_1.raw #6033 RT: 19.62
FTMS, HCD@27.00, z=+2, Mono m/z=1064.03064 Da, MH+=2127.05400 Da, Match Tol.=0.05 Da

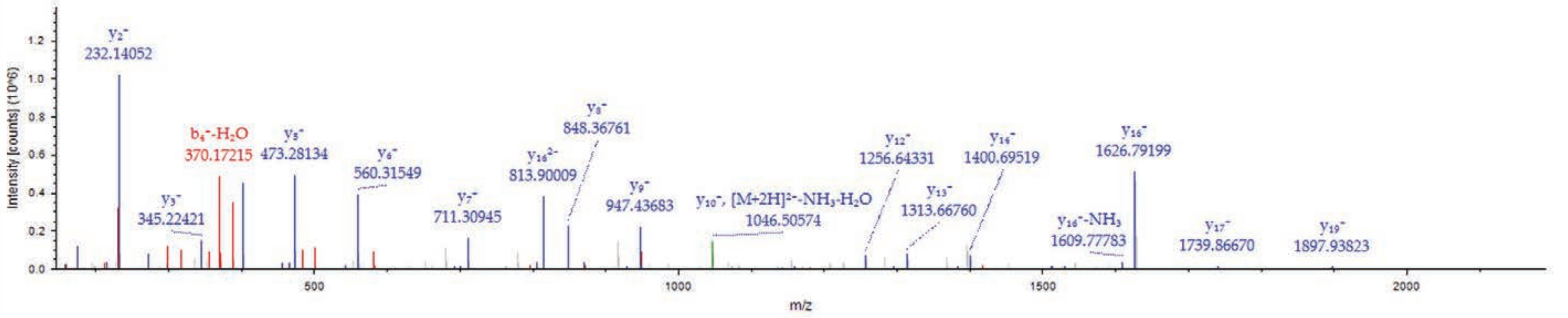

| Uniprot Accession | Description | Gene name | PTP type | Sequence | Hyperoxidised cysteine | Total MS/MS count |
|---|---|---|---|---|---|---|
| Q6PEI4 | Protein tyrosine phosphatase, non-receptor type 12 | ptpn12 | Non-receptor | DTTPIcVHcSAGcGR | C13(hyper) | 71 |

Peptide Summary

Sequence: DTTPICVHCSAGCGR, C6-Carbamidomethyl (57.02146 Da), C9-hyperoxidation (47.98474 Da)
Charge: +2, Monoisotopic m/z: 841.33966 Da (+28527.44 mmu/+33907.17 ppm), MH+: 1681.67204 Da, RT: 15.04 min,
Identified with: Mascot (v1.30); IonScore:102, Exp Value:2.3E-009, Ions matched by search engine: 10/130
Fragment match tolerance used for search: 0.05 Da

Fragment Matches

Value Type: Theo. Mass [Da]

Ion Series | Neutral Losses | Precursor Ions

| #1 | b+ | b2+ | Seq. | y+ | y2+ | #2 |
|---|---|---|---|---|---|---|
| 1 | 116.03423 | 58.52075 | D | | | 15 |
| 2 | 217.08191 | 109.04459 | T | 1509.62436 | 755.31582 | 14 |
| 3 | 318.12959 | 159.56843 | T | 1408.57668 | 704.79198 | 13 |
| 4 | 415.18236 | 208.09482 | P | 1307.52900 | 654.26814 | 12 |
| 5 | 528.26643 | 264.63685 | I | 1210.47623 | 605.74175 | 11 |
| 6 | 688.29708 | 344.65218 | C-Carbami... | 1097.39216 | 549.19972 | 10 |
| 7 | 787.36550 | 394.18639 | V | 937.36151 | 469.18439 | 9 |
| 8 | 924.42441 | 462.71584 | H | 838.29309 | 419.65018 | 8 |
| 9 | 1075.41834 | 538.21281 | C-hyper | 701.23418 | 351.12073 | 7 |
| 10 | 1162.45037 | 581.72883 | S | 550.24024 | 275.62376 | 6 |
| 11 | 1233.48749 | 617.24739 | A | 463.20821 | 232.10774 | 5 |
| 12 | 1290.50896 | 645.75812 | G | 392.17109 | 196.58918 | 4 |
| 13 | 1393.51815 | 697.26272 | C | 335.14962 | 168.07845 | 3 |
| 14 | 1450.53962 | 725.77345 | G | 232.14043 | 116.57385 | 2 |
| 15 | | | R | 175.11896 | 88.06312 | 1 |

Fragment Spectrum

Extracted from: D:\wu000024\20161117_Fin05_3reps\Set3\20161120_OR12_A6_WW_Fin05_13_2.raw #4523 RT: 15.04
FTMS, HCD@27.00, z=+2, Mono m/z=841.33966 Da, MH+=1681.67204 Da, Match Tol.=0.05 Da

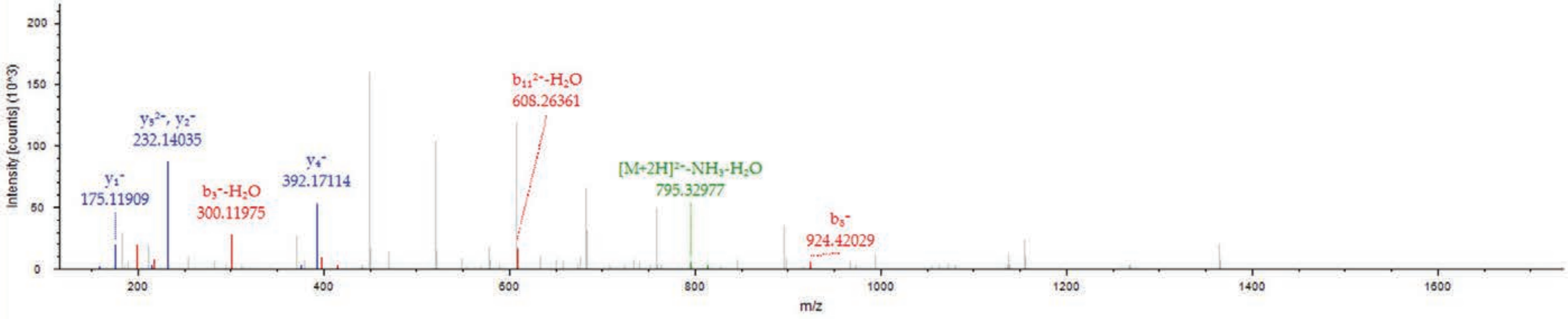

| Uniprot Accession | Description | Gene name | PTP type | Sequence | Hyperoxidised cysteine | Total MS/MS count |
|---|---|---|---|---|---|---|
| C7U124 | Protein-tyrosine phosphatase BAS (Fragment) | ptpn13 | Non-receptor | HIHLSGPII**THcSAGIGR** | C12(hyper) | 64 |

Peptide Summary

Sequence: HIHLSGPIITHCSAGIGR, C12-hyperoxidation (47.98474 Da)
Charge: +2, Monoisotopic m/z: 958.99292 Da (+1.27 mmu/+1.33 ppm), MH+: 1916.97856 Da, RT: 18.52 min,
Identified with: Mascot (v1.30); IonScore:111, Exp Value:1.0E-009, Ions matched by search engine: 13/152
Fragment match tolerance used for search: 0.05 Da

Fragment Matches

Value Type: Theo. Mass [Da]

Ion Series | Neutral Losses | Precursor Ions

| #1 | $b^+$ | $b^{2+}$ | Seq. | $y^+$ | $y^{2+}$ | #2 |
|---|---|---|---|---|---|---|
| 1 | 138.06619 | 69.53673 | H | | | 18 |
| 2 | 251.15026 | 126.07877 | I | 1779.91711 | 890.46219 | 17 |
| 3 | 388.20917 | 194.60822 | H | 1666.83304 | 833.92016 | 16 |
| 4 | 501.29324 | 251.15026 | L | 1529.77413 | 765.39070 | 15 |
| 5 | 588.32527 | 294.66627 | S | 1416.69006 | 708.84867 | 14 |
| 6 | 645.34674 | 323.17701 | G | 1329.65803 | 665.33265 | 13 |
| 7 | 742.39951 | 371.70339 | P | 1272.63656 | 636.82192 | 12 |
| 8 | 855.48358 | 428.24543 | I | 1175.58379 | 588.29553 | 11 |
| 9 | 968.56765 | 484.78746 | I | 1062.49972 | 531.75350 | 10 |
| 10 | 1069.61533 | 535.31130 | T | 949.41565 | 475.21146 | 9 |
| 11 | 1206.67424 | 603.84076 | H | 848.36797 | 424.68762 | 8 |
| 12 | 1357.66817 | 679.33772 | C-hyper | 711.30906 | 356.15817 | 7 |
| 13 | 1444.70020 | 722.85374 | S | 560.31512 | 280.66120 | 6 |
| 14 | 1515.73732 | 758.37230 | A | 473.28309 | 237.14518 | 5 |
| 15 | 1572.75879 | 786.88303 | G | 402.24597 | 201.62662 | 4 |
| 16 | 1685.84286 | 843.42507 | I | 345.22450 | 173.11589 | 3 |
| 17 | 1742.86433 | 871.93580 | G | 232.14043 | 116.57385 | 2 |
| 18 | | | R | 175.11896 | 88.06312 | 1 |

Fragment Spectrum

Extracted from: D:\wu000024\20161117_Fin05_3reps\Set3\20161120_OR12_A6_WW_Fin05_13_5.raw #5994 RT: 18.52
FTMS, HCD@27.00, z=+2, Mono m/z=958.99292 Da, MH+=1916.97856 Da, Match Tol.=0.05 Da

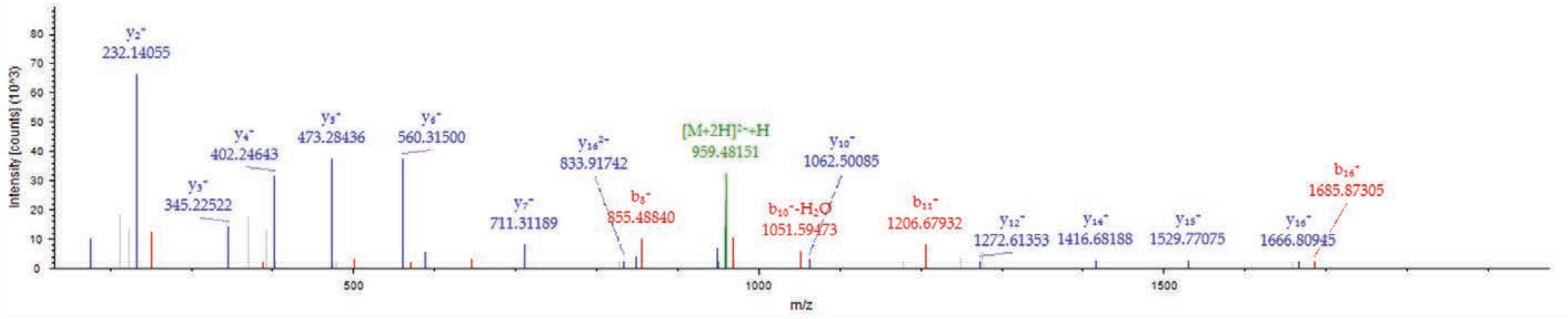

| Uniprot Accession | Description | Gene name | PTP type | Sequence | Hyperoxidised cysteine | Total MS/MS count |
|---|---|---|---|---|---|---|
| F1QIX3 | Uncharacterized protein | ptpn20 | Non-receptor | GPITVHcSAGIGR | C7(hyper) | 38 |

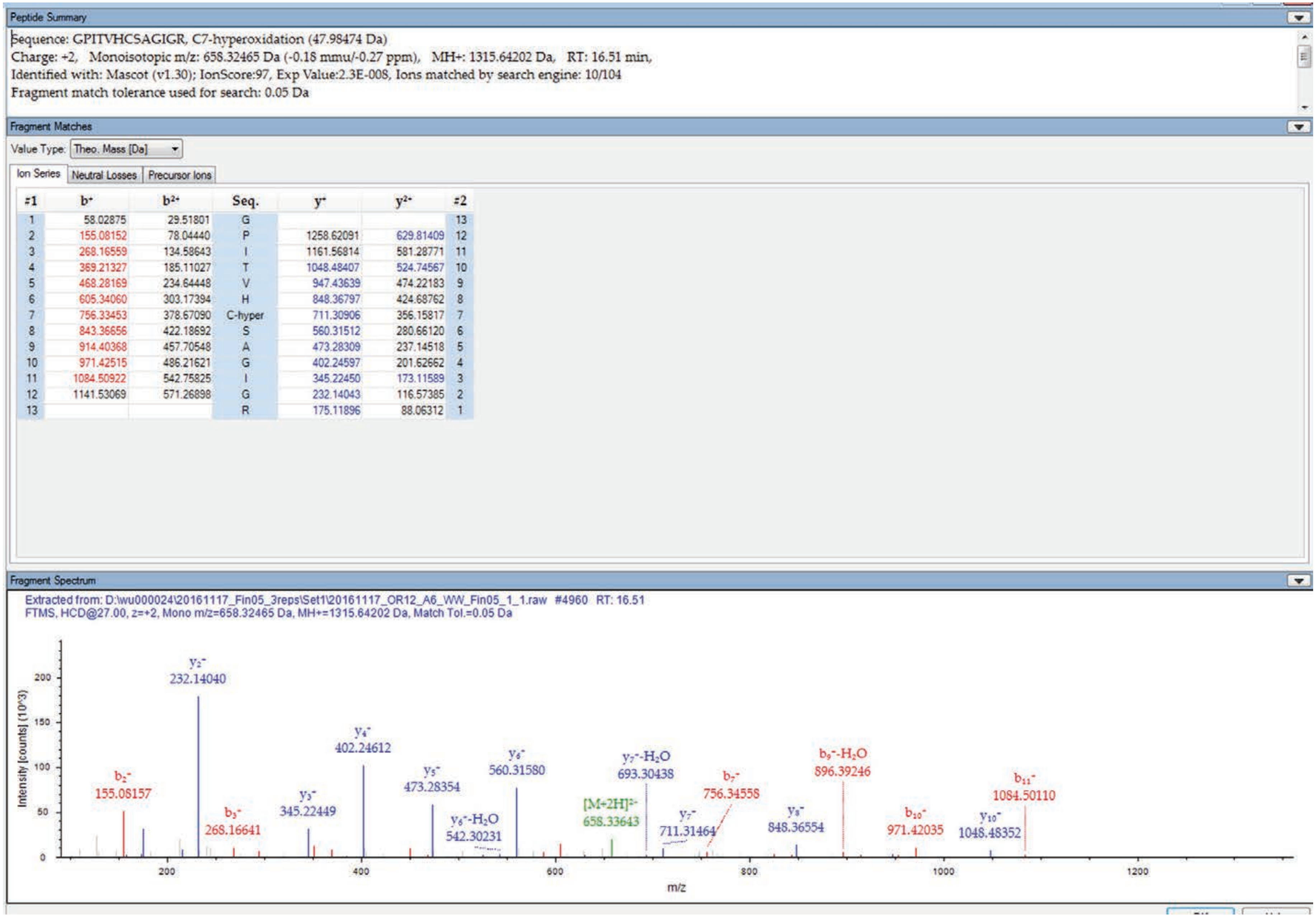

Peptide Summary

Sequence: GPITVHCSAGIGR, C7-hyperoxidation (47.98474 Da)
Charge: +2, Monoisotopic m/z: 658.32465 Da (-0.18 mmu/-0.27 ppm), MH+: 1315.64202 Da, RT: 16.51 min,
Identified with: Mascot (v1.30); IonScore:97, Exp Value:2.3E-008, Ions matched by search engine: 10/104
Fragment match tolerance used for search: 0.05 Da

Fragment Matches

Value Type: Theo. Mass [Da]

Ion Series | Neutral Losses | Precursor Ions

| #1 | b+ | b2+ | Seq. | y+ | y2+ | #2 |
|---|---|---|---|---|---|---|
| 1 | 58.02875 | 29.51801 | G | | | 13 |
| 2 | 155.08152 | 78.04440 | P | 1258.62091 | 629.81409 | 12 |
| 3 | 268.16559 | 134.58643 | I | 1161.56814 | 581.28771 | 11 |
| 4 | 369.21327 | 185.11027 | T | 1048.48407 | 524.74567 | 10 |
| 5 | 468.28169 | 234.64448 | V | 947.43639 | 474.22183 | 9 |
| 6 | 605.34060 | 303.17394 | H | 848.36797 | 424.68762 | 8 |
| 7 | 756.33453 | 378.67090 | C-hyper | 711.30906 | 356.15817 | 7 |
| 8 | 843.36656 | 422.18692 | S | 560.31512 | 280.66120 | 6 |
| 9 | 914.40368 | 457.70548 | A | 473.28309 | 237.14518 | 5 |
| 10 | 971.42515 | 486.21621 | G | 402.24597 | 201.62662 | 4 |
| 11 | 1084.50922 | 542.75825 | I | 345.22450 | 173.11589 | 3 |
| 12 | 1141.53069 | 571.26898 | G | 232.14043 | 116.57385 | 2 |
| 13 | | | R | 175.11896 | 88.06312 | 1 |

Fragment Spectrum

| Uniprot Accession | Description | Gene name | PTP type | Sequence | Hyperoxidised cysteine | Total MS/MS count |
|---|---|---|---|---|---|---|
| F1Q6T9 | Uncharacterized protein | ptpn21 | Non-receptor | NTNLPVLVHcSAGVGR | C10(hyper) | 29 |

Peptide Summary

Sequence: NTNLPVLVHCSAGVGR, C10-hyperoxidation (47.98474 Da)
Charge: +2, Monoisotopic m/z: 842.92761 Da (+2.17 mmu/+2.58 ppm), MH+: 1684.84795 Da, RT: 19.95 min,
Identified with: Mascot (v1.30); IonScore:123, Exp Value:7.2E-011, Ions matched by search engine: 11/168
Fragment match tolerance used for search: 0.05 Da

Fragment Matches

Value Type: Theo. Mass [Da]

Ion Series | Neutral Losses | Precursor Ions

| #1 | $b^+$ | $b^{2+}$ | Seq. | $y^+$ | $y^{2+}$ | #2 |
|---|---|---|---|---|---|---|
| 1 | 115.05021 | 58.02874 | N | | | 16 |
| 2 | 216.09789 | 108.55258 | T | 1570.80068 | 785.90398 | 15 |
| 3 | 330.14082 | 165.57405 | N | 1469.75300 | 735.38014 | 14 |
| 4 | 443.22489 | 222.11608 | L | 1355.71007 | 678.35867 | 13 |
| 5 | 540.27766 | 270.64247 | P | 1242.62600 | 621.81664 | 12 |
| 6 | 639.34608 | 320.17668 | V | 1145.57323 | 573.29025 | 11 |
| 7 | 752.43015 | 376.71871 | L | 1046.50481 | 523.75604 | 10 |
| 8 | 851.49857 | 426.25292 | V | 933.42074 | 467.21401 | 9 |
| 9 | 988.55748 | 494.78238 | H | 834.35232 | 417.67980 | 8 |
| 10 | 1139.55141 | 570.27934 | C-hyper | 697.29341 | 349.15034 | 7 |
| 11 | 1226.58344 | 613.79536 | S | 546.29947 | 273.65337 | 6 |
| 12 | 1297.62056 | 649.31392 | A | 459.26744 | 230.13736 | 5 |
| 13 | 1354.64203 | 677.82465 | G | 388.23032 | 194.61880 | 4 |
| 14 | 1453.71045 | 727.35886 | V | 331.20885 | 166.10806 | 3 |
| 15 | 1510.73192 | 755.86960 | G | 232.14043 | 116.57385 | 2 |
| 16 | | | R | 175.11896 | 88.06312 | 1 |

Fragment Spectrum

Extracted from: D:\wu000024\20161117_Fin05_3reps\Set2\20161118_OR12_A6_WW_Fin05_7_2.raw #6150 RT: 19.95
FTMS, HCD@27.00, z=+2, Mono m/z=842.92761 Da, MH+=1684.84795 Da, Match Tol.=0.05 Da

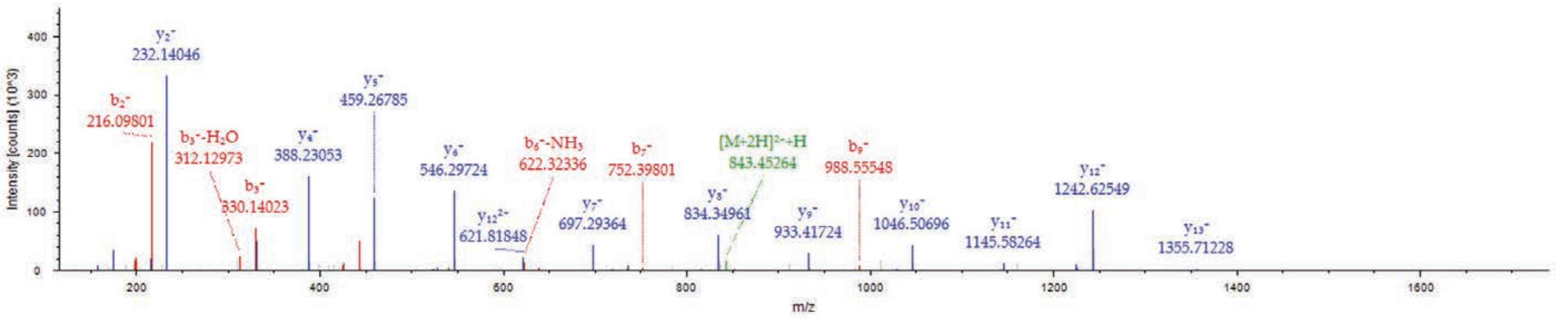

| Uniprot Accession | Description | Gene name | PTP type | Sequence | Hyperoxidised cysteine | Total MS/MS count |
|---|---|---|---|---|---|---|
| C7U128 | Protein-tyrosine phosphatase LyPTP (Fragment) | ptpn22 | Non-receptor | IYQDNEEIPIcIHcSAGcGR | C18(hyper) | 13 |

Peptide Summary

Sequence: IYQDNEEIPICIHCSAGCGR, C11-Carbamidomethyl (57.02146 Da), C14-hyperoxidation (47.98474 Da)
Charge: +2, Monoisotopic m/z: 1192.01038 Da (+28523.9 mmu/+23929.24 ppm), MH+: 2383.01348 Da, RT: 20.68 min,
Identified with: Mascot (v1.30); IonScore:99, Exp Value:9.1E-009, Ions matched by search engine: 10/208
Fragment match tolerance used for search: 0.05 Da

Fragment Matches

Value Type: Theo. Mass [Da]

Ion Series | Neutral Losses | Precursor Ions

| #1 | $b^+$ | $b^{2+}$ | Seq. | $y^+$ | $y^{2+}$ | #2 |
|---|---|---|---|---|---|---|
| 1 | 114.09135 | 57.54931 | I | | | 20 |
| 2 | 277.15467 | 139.08097 | Y | 2212.90570 | 1106.95649 | 19 |
| 3 | 405.21325 | 203.11026 | Q | 2049.84238 | 1025.42483 | 18 |
| 4 | 520.24020 | 260.62374 | D | 1921.78380 | 961.39554 | 17 |
| 5 | 634.28313 | 317.64520 | N | 1806.75685 | 903.88206 | 16 |
| 6 | 763.32573 | 382.16650 | E | 1692.71392 | 846.86060 | 15 |
| 7 | 892.36833 | 446.68780 | E | 1563.67132 | 782.33930 | 14 |
| 8 | 1005.45240 | 503.22984 | I | 1434.62872 | 717.81800 | 13 |
| 9 | 1102.50517 | 551.75622 | P | 1321.54465 | 661.27596 | 12 |
| 10 | 1215.58924 | 608.29826 | I | 1224.49188 | 612.74958 | 11 |
| 11 | 1375.61989 | 688.31358 | C-Carbami... | 1111.40781 | 556.20754 | 10 |
| 12 | 1488.70396 | 744.85562 | I | 951.37716 | 476.19222 | 9 |
| 13 | 1625.76287 | 813.38507 | H | 838.29309 | 419.65018 | 8 |
| 14 | 1776.75680 | 888.88204 | C-hyper | 701.23418 | 351.12073 | 7 |
| 15 | 1863.78883 | 932.39806 | S | 550.24024 | 275.62376 | 6 |
| 16 | 1934.82595 | 967.91662 | A | 463.20821 | 232.10774 | 5 |
| 17 | 1991.84742 | 996.42735 | G | 392.17109 | 196.58918 | 4 |
| 18 | 2094.85661 | 1047.93195 | C | 335.14962 | 168.07845 | 3 |
| 19 | 2151.87808 | 1076.44268 | G | 232.14043 | 116.57385 | 2 |
| 20 | | | R | 175.11896 | 88.06312 | 1 |

Fragment Spectrum

Extracted from: D:\wu000024\20161117_Fin05_3reps\Set3\20161120_OR12_A6_WW_Fin05_16_1.raw #6432 RT: 20.68
FTMS, HCD@27.00, z=+2, Mono m/z=1192.01038 Da, MH+=2383.01348 Da, Match Tol.=0.05 Da

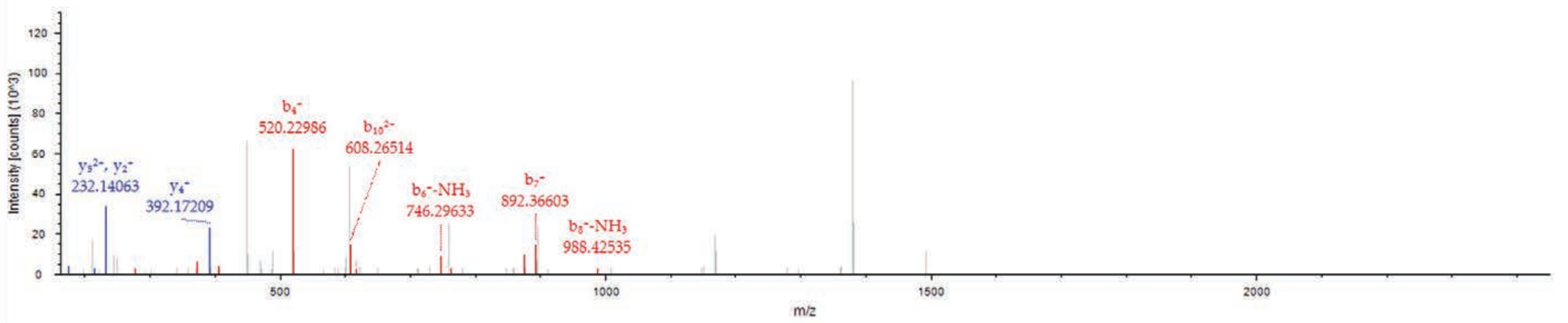

| Uniprot Accession | Description | Gene name | PTP type | Sequence | Hyperoxidised cysteine | Total MS/MS count |
|---|---|---|---|---|---|---|
| F1R1D1 | Uncharacterized protein | ptpn23a | Non-receptor | VScTHFQcSAGAFAYLR | C8(hyper) | 138 |

Peptide Summary

Sequence: VSCTHFQCSAGAFAYLR, C3-Carbamidomethyl (57.02146 Da), C8-hyperoxidation (47.98474 Da)
Charge: +2, Monoisotopic m/z: 983.43164 Da (-1.13 mmu/-1.15 ppm), MH+: 1965.85600 Da, RT: 23.60 min,
Identified with: Mascot (v1.30); IonScore:72, Exp Value:5.4E-006, Ions matched by search engine: 8/162
Fragment match tolerance used for search: 0.05 Da

Fragment Matches

Value Type: Theo. Mass [Da]

Ion Series | Neutral Losses | Precursor Ions

| #1 | b+ | b2+ | Seq. | y+ | y2+ | #2 |
|---|---|---|---|---|---|---|
| 1 | 100.07570 | 50.54149 | V | | | 17 |
| 2 | 187.10773 | 94.05750 | S | 1866.78984 | 933.89856 | 16 |
| 3 | 347.13838 | 174.07283 | C-Carbami... | 1779.75781 | 890.38254 | 15 |
| 4 | 448.18606 | 224.59667 | T | 1619.72716 | 810.36722 | 14 |
| 5 | 585.24497 | 293.12612 | H | 1518.67948 | 759.84338 | 13 |
| 6 | 732.31339 | 366.66033 | F | 1381.62057 | 691.31392 | 12 |
| 7 | 860.37197 | 430.68962 | Q | 1234.55215 | 617.77971 | 11 |
| 8 | 1011.36590 | 506.18659 | C-hyper | 1106.49357 | 553.75042 | 10 |
| 9 | 1098.39793 | 549.70261 | S | 955.49963 | 478.25345 | 9 |
| 10 | 1169.43505 | 585.22117 | A | 868.46760 | 434.73744 | 8 |
| 11 | 1226.45652 | 613.73190 | G | 797.43048 | 399.21888 | 7 |
| 12 | 1297.49364 | 649.25046 | A | 740.40901 | 370.70814 | 6 |
| 13 | 1444.56206 | 722.78467 | F | 669.37189 | 335.18958 | 5 |
| 14 | 1515.59918 | 758.30323 | A | 522.30347 | 261.65537 | 4 |
| 15 | 1678.66250 | 839.83489 | Y | 451.26635 | 226.13681 | 3 |
| 16 | 1791.74657 | 896.37693 | L | 288.20303 | 144.60515 | 2 |
| 17 | | | R | 175.11896 | 88.06312 | 1 |

Fragment Spectrum

Extracted from: D:\wu000024\20161117_Fin05_3reps\Set2\20161118_OR12_A6_WW_Fin05_7_1.raw #7717 RT: 23.60
FTMS, HCD@27.00, z=+2, Mono m/z=983.43164 Da, MH+=1965.85600 Da, Match Tol.=0.05 Da

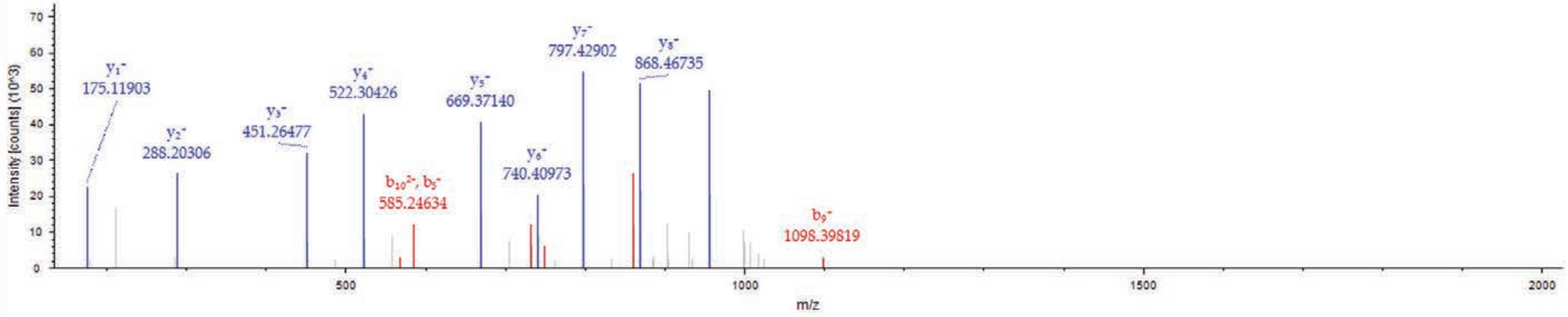

| Uniprot Accession | Description | Gene name | PTP type | Sequence | Hyperoxidised cysteine | Total MS/MS count |
|---|---|---|---|---|---|---|
| F1QXQ5 (F1QGX6) | Tyrosine-protein phosphatase non-receptor type | ptpn2a | Non-receptor | ESGSLGPENGPVVVHcSAGIGR | C16(hyper) | 86 |

Peptide Summary

Sequence: ESGSLGPENGPVVVHCSAGIGR, C16-hyperoxidation (47.98474 Da)
Charge: +2, Monoisotopic m/z: 1085.51453 Da (+1.17 mmu/+1.08 ppm), MH+: 2170.02178 Da, RT: 18.70 min,
Identified with: Mascot (v1.30); IonScore:139, Exp Value:1.8E-012, Ions matched by search engine: 14/226
Fragment match tolerance used for search: 0.05 Da

Fragment Matches

Value Type: Theo. Mass [Da]

Ion Series | Neutral Losses | Precursor Ions

| #1 | $b^+$ | $b^{2+}$ | Seq. | $y^+$ | $y^{2+}$ | #2 |
|---|---|---|---|---|---|---|
| 1 | 130.04988 | 65.52858 | E | | | 22 |
| 2 | 217.08191 | 109.04459 | S | 2040.97684 | 1020.99206 | 21 |
| 3 | 274.10338 | 137.55533 | G | 1953.94481 | 977.47604 | 20 |
| 4 | 361.13541 | 181.07134 | S | 1896.92334 | 948.96531 | 19 |
| 5 | 474.21948 | 237.61338 | L | 1809.89131 | 905.44929 | 18 |
| 6 | 531.24095 | 266.12411 | G | 1696.80724 | 848.90726 | 17 |
| 7 | 628.29372 | 314.65050 | P | 1639.78577 | 820.39652 | 16 |
| 8 | 757.33632 | 379.17180 | E | 1542.73300 | 771.87014 | 15 |
| 9 | 871.37925 | 436.19326 | N | 1413.69040 | 707.34884 | 14 |
| 10 | 928.40072 | 464.70400 | G | 1299.64747 | 650.32737 | 13 |
| 11 | 1025.45349 | 513.23038 | P | 1242.62600 | 621.81664 | 12 |
| 12 | 1124.52191 | 562.76459 | V | 1145.57323 | 573.29025 | 11 |
| 13 | 1223.59033 | 612.29880 | V | 1046.50481 | 523.75604 | 10 |
| 14 | 1322.65875 | 661.83301 | V | 947.43639 | 474.22183 | 9 |
| 15 | 1459.71766 | 730.36247 | H | 848.36797 | 424.68762 | 8 |
| 16 | 1610.71159 | 805.85943 | C-hyper | 711.30906 | 356.15817 | 7 |
| 17 | 1697.74362 | 849.37545 | S | 560.31512 | 280.66120 | 6 |
| 18 | 1768.78074 | 884.89401 | A | 473.28309 | 237.14518 | 5 |
| 19 | 1825.80221 | 913.40474 | G | 402.24597 | 201.62662 | 4 |
| 20 | 1938.88628 | 969.94678 | I | 345.22450 | 173.11589 | 3 |
| 21 | 1995.90775 | 998.45751 | G | 232.14043 | 116.57385 | 2 |
| 22 | | | R | 175.11896 | 88.06312 | 1 |

Fragment Spectrum

Extracted from: D:\wu000024\20161117_Fin05_3reps\Set3\20161120_OR12_A6_WW_Fin05_13_4.raw #5893 RT: 18.70
FTMS, HCD@27.00, z=+2, Mono m/z=1085.51453 Da, MH+=2170.02178 Da, Match Tol.=0.05 Da

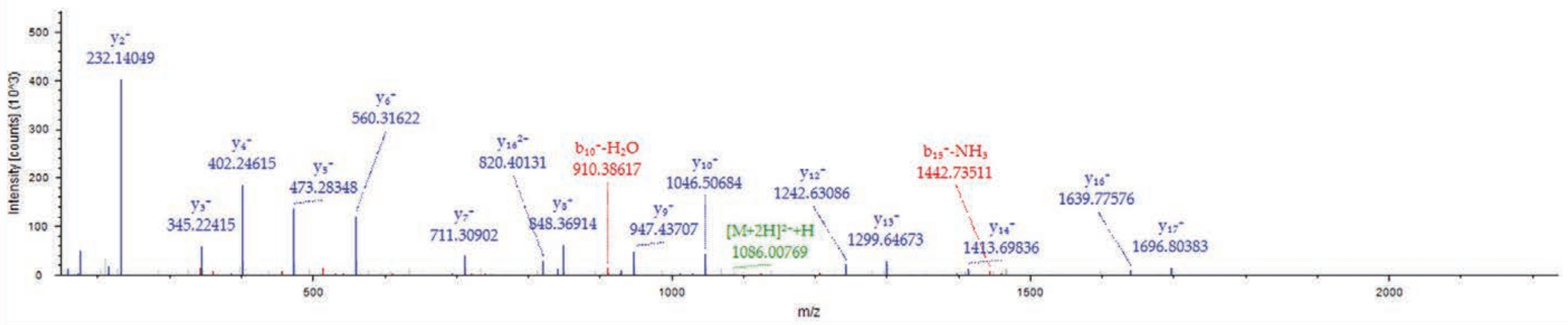

| Uniprot Accession | Description | Gene name | PTP type | Sequence | Hyperoxidised cysteine | Total MS/MS count |
|---|---|---|---|---|---|---|
| Q6NY86 | Tyrosine-protein phosphatase non-receptor type | ptpn2b | Non-receptor | ESGSLGMEQGPAV**VHcSAGIGR** | C16(hyper) | 159 |

**Peptide Summary**

Sequence: ESGSLGMEQGPAVVHCSAGIGR, M7-Oxidation (15.99492 Da), C16-hyperoxidation (47.98474 Da)
Charge: +2, Monoisotopic m/z: 1103.49744 Da (+0.58 mmu/+0.53 ppm), MH+: 2205.98760 Da, RT: 16.47 min,
Identified with: Mascot (v1.30); IonScore:140, Exp Value:1.0E-012, Ions matched by search engine: 13/226
Fragment match tolerance used for search: 0.05 Da

**Fragment Matches**

Value Type: Theo. Mass [Da]

Ion Series | Neutral Losses | Precursor Ions

| #1 | $b^+$ | $b^{2+}$ | Seq. | $y^+$ | $y^{2+}$ | #2 |
|---|---|---|---|---|---|---|
| 1 | 130.04988 | 65.52858 | E | | | 22 |
| 2 | 217.08191 | 109.04459 | S | 2076.94383 | 1038.97555 | 21 |
| 3 | 274.10338 | 137.55533 | G | 1989.91180 | 995.45954 | 20 |
| 4 | 361.13541 | 181.07134 | S | 1932.89033 | 966.94880 | 19 |
| 5 | 474.21948 | 237.61338 | L | 1845.85830 | 923.43279 | 18 |
| 6 | 531.24095 | 266.12411 | G | 1732.77423 | 866.89075 | 17 |
| 7 | 678.27636 | 339.64182 | M-Oxidation | 1675.75276 | 838.38002 | 16 |
| 8 | 807.31896 | 404.16312 | E | 1528.71735 | 764.86231 | 15 |
| 9 | 935.37754 | 468.19241 | Q | 1399.67475 | 700.34101 | 14 |
| 10 | 992.39901 | 496.70314 | G | 1271.61617 | 636.31172 | 13 |
| 11 | 1089.45178 | 545.22953 | P | 1214.59470 | 607.80099 | 12 |
| 12 | 1160.48890 | 580.74809 | A | 1117.54193 | 559.27460 | 11 |
| 13 | 1259.55732 | 630.28230 | V | 1046.50481 | 523.75604 | 10 |
| 14 | 1358.62574 | 679.81651 | V | 947.43639 | 474.22183 | 9 |
| 15 | 1495.68465 | 748.34596 | H | 848.36797 | 424.68762 | 8 |
| 16 | 1646.67859 | 823.84293 | C-hyper | 711.30906 | 356.15817 | 7 |
| 17 | 1733.71062 | 867.35895 | S | 560.31512 | 280.66120 | 6 |
| 18 | 1804.74774 | 902.87751 | A | 473.28309 | 237.14518 | 5 |
| 19 | 1861.76921 | 931.38824 | G | 402.24597 | 201.62662 | 4 |
| 20 | 1974.85328 | 987.93028 | I | 345.22450 | 173.11589 | 3 |
| 21 | 2031.87475 | 1016.44101 | G | 232.14043 | 116.57385 | 2 |
| 22 | | | R | 175.11896 | 88.06312 | 1 |

**Fragment Spectrum**

Extracted from: D:\wu000024\20161117_Fin05_3reps\Set2\20161118_OR12_A6_WW_Fin05_7_4.raw #5037 RT: 16.47
FTMS, HCD@27.00, z=+2, Mono m/z=1103.49744 Da, MH+=2205.98760 Da, Match Tol.=0.05 Da

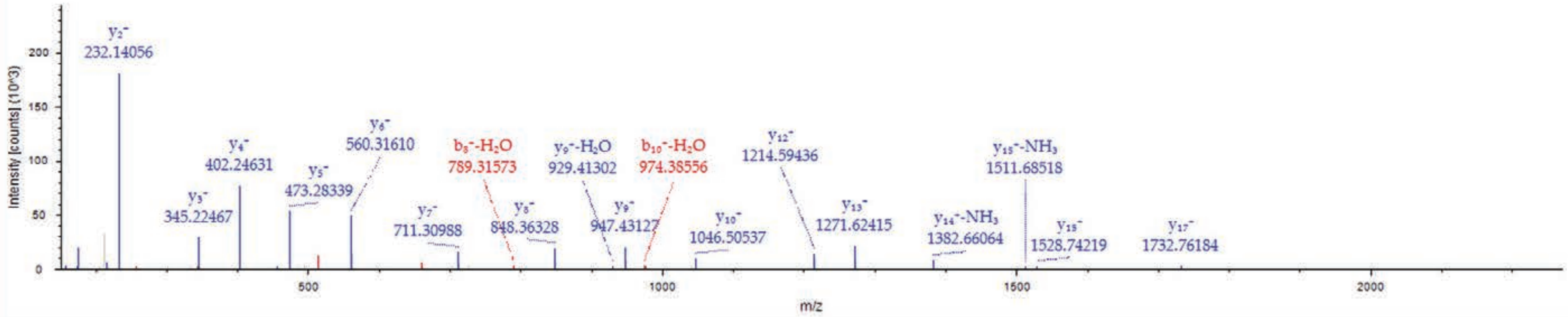

| Uniprot Accession | Description | Gene name | PTP type | Sequence | Hyperoxidised cysteine | Total MS/MS count |
|---|---|---|---|---|---|---|
| F1QLN8 | Uncharacterized protein | ptpn3 | Non-receptor | QESVPLMVHcSAGIGR | C10(hyper) | 97 |

Peptide Summary

Sequence: QESVPLMVHCSAGIGR, C10-hyperoxidation (47.98474 Da)
Charge: +2, Monoisotopic m/z: 866.41370 Da (+2.38 mmu/+2.75 ppm), MH+: 1731.82012 Da, RT: 20.36 min,
Identified with: Mascot (v1.30); IonScore:114, Exp Value:4.8E-010, Ions matched by search engine: 14/168
Fragment match tolerance used for search: 0.05 Da

Fragment Matches

Value Type: Theo. Mass [Da]

Ion Series | Neutral Losses | Precursor Ions

| #1 | b+ | b2+ | Seq. | y+ | y2+ | #2 |
|---|---|---|---|---|---|---|
| 1 | 129.06586 | 65.03657 | Q | | | 16 |
| 2 | 258.10846 | 129.55787 | E | 1603.75678 | 802.38203 | 15 |
| 3 | 345.14049 | 173.07388 | S | 1474.71418 | 737.86073 | 14 |
| 4 | 444.20891 | 222.60809 | V | 1387.68215 | 694.34471 | 13 |
| 5 | 541.26168 | 271.13448 | P | 1288.61373 | 644.81050 | 12 |
| 6 | 654.34575 | 327.67651 | L | 1191.56096 | 596.28412 | 11 |
| 7 | 785.38625 | 393.19676 | M | 1078.47689 | 539.74208 | 10 |
| 8 | 884.45467 | 442.73097 | V | 947.43639 | 474.22183 | 9 |
| 9 | 1021.51358 | 511.26043 | H | 848.36797 | 424.68762 | 8 |
| 10 | 1172.50751 | 586.75739 | C-hyper | 711.30906 | 356.15817 | 7 |
| 11 | 1259.53954 | 630.27341 | S | 560.31512 | 280.66120 | 6 |
| 12 | 1330.57666 | 665.79197 | A | 473.28309 | 237.14518 | 5 |
| 13 | 1387.59813 | 694.30270 | G | 402.24597 | 201.62662 | 4 |
| 14 | 1500.68220 | 750.84474 | I | 345.22450 | 173.11589 | 3 |
| 15 | 1557.70367 | 779.35547 | G | 232.14043 | 116.57385 | 2 |
| 16 | | | R | 175.11896 | 88.06312 | 1 |

Fragment Spectrum

Extracted from: D:\wu000024\20161117_Fin05_3reps\Set3\20161120_OR12_A6_WW_Fin05_16_1.raw #6311 RT: 20.36
FTMS, HCD@27.00, z=+2, Mono m/z=866.41370 Da, MH+=1731.82012 Da, Match Tol.=0.05 Da

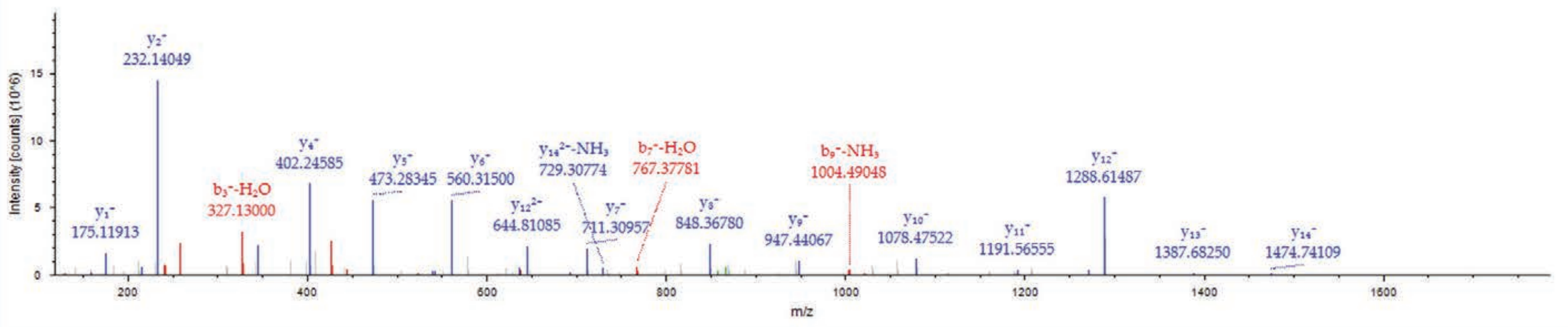

| Uniprot Accession | Description | Gene name | PTP type | Sequence | Hyperoxidised cysteine | Total MS/MS count |
|---|---|---|---|---|---|---|
| C7U127 | Protein-tyrosine phosphatase MEG1a (Fragment) | ptpn4a | Non-receptor | DEPVVVHcSAGIGR | C8(hyper) | 19 |

Peptide Summary

Sequence: DEPVVVHCSAGIGR, C8-hyperoxidation (47.98474 Da)
Charge: +2, Monoisotopic m/z: 743.85236 Da (+0.95 mmu/+1.27 ppm), MH+: 1486.69744 Da, RT: 16.66 min,
Identified with: Mascot (v1.30); IonScore:113, Exp Value:6.2E-010, Ions matched by search engine: 10/120
Fragment match tolerance used for search: 0.05 Da

Fragment Matches

Value Type: Theo. Mass [Da]

Ion Series | Neutral Losses | Precursor Ions

| #1 | $b^+$ | $b^{2+}$ | Seq. | $y^+$ | $y^{2+}$ | #2 |
|---|---|---|---|---|---|---|
| 1 | 116.03423 | 58.52075 | D | | | 14 |
| 2 | 245.07683 | 123.04205 | E | 1371.66860 | 686.33794 | 13 |
| 3 | 342.12960 | 171.56844 | P | 1242.62600 | 621.81664 | 12 |
| 4 | 441.19802 | 221.10265 | V | 1145.57323 | 573.29025 | 11 |
| 5 | 540.26644 | 270.63686 | V | 1046.50481 | 523.75604 | 10 |
| 6 | 639.33486 | 320.17107 | V | 947.43639 | 474.22183 | 9 |
| 7 | 776.39377 | 388.70052 | H | 848.36797 | 424.68762 | 8 |
| 8 | 927.38770 | 464.19749 | C-hyper | 711.30906 | 356.15817 | 7 |
| 9 | 1014.41973 | 507.71350 | S | 560.31512 | 280.66120 | 6 |
| 10 | 1085.45685 | 543.23206 | A | 473.28309 | 237.14518 | 5 |
| 11 | 1142.47832 | 571.74280 | G | 402.24597 | 201.62662 | 4 |
| 12 | 1255.56239 | 628.28483 | I | 345.22450 | 173.11589 | 3 |
| 13 | 1312.58386 | 656.79557 | G | 232.14043 | 116.57385 | 2 |
| 14 | | | R | 175.11896 | 88.06312 | 1 |

Fragment Spectrum

Extracted from: D:\wu000024\20161117_Fin05_3reps\Set3\20161120_OR12_A6_WW_Fin05_13_1.raw #5125 RT: 16.66
FTMS, HCD@27.00, z=+2, Mono m/z=743.85236 Da, MH+=1486.69744 Da, Match Tol.=0.05 Da

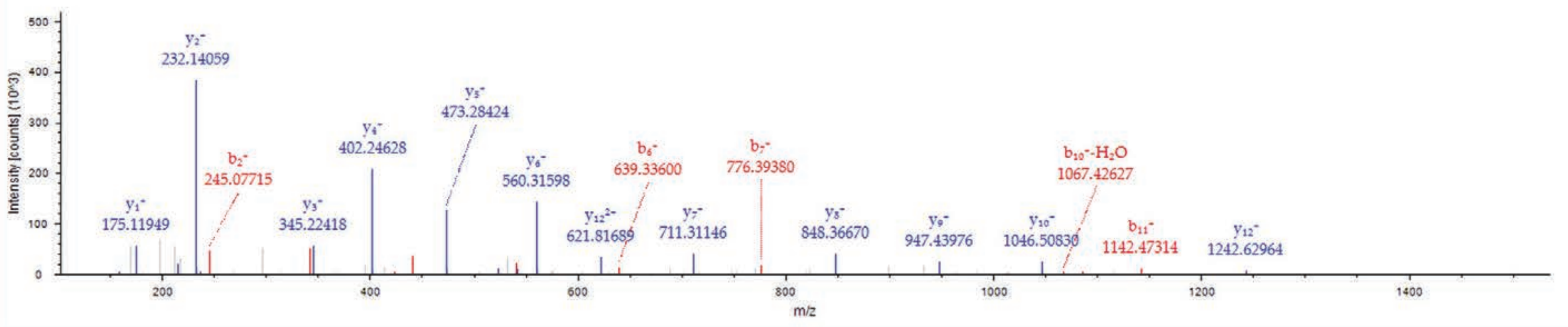

| Uniprot Accession | Description | Gene name | PTP type | Sequence | Hyperoxidised cysteine | Total MS/MS count |
|---|---|---|---|---|---|---|
| C7U113 | Protein-tyrosine phosphatase MEG1a (Fragment) | ptpn4b | Non-receptor | ADGPEPVVVHcSAGIGR | C11(hyper) | 156 |

**Peptide Summary**

Sequence: ADGPEPVVVHCSAGIGR, C11-hyperoxidation (47.98474 Da)
Charge: +2, Monoisotopic m/z: 856.40961 Da (+2.52 mmu/+2.94 ppm), MH+: 1711.81194 Da, RT: 17.34 min,
Identified with: Mascot (v1.30); IonScore:98, Exp Value:2.4E-008, Ions matched by search engine: 12/148
Fragment match tolerance used for search: 0.05 Da

**Fragment Matches**

Value Type: Theo. Mass [Da]

Ion Series | Neutral Losses | Precursor Ions

| #1 | b⁺ | b²⁺ | Seq. | y⁺ | y²⁺ | #2 |
|---|---|---|---|---|---|---|
| 1 | 72.04440 | 36.52584 | A | | | 17 |
| 2 | 187.07135 | 94.03931 | D | 1640.76979 | 820.88853 | 16 |
| 3 | 244.09282 | 122.55005 | G | 1525.74284 | 763.37506 | 15 |
| 4 | 341.14559 | 171.07643 | P | 1468.72137 | 734.86432 | 14 |
| 5 | 470.18819 | 235.59773 | E | 1371.66860 | 686.33794 | 13 |
| 6 | 567.24096 | 284.12412 | P | 1242.62600 | 621.81664 | 12 |
| 7 | 666.30938 | 333.65833 | V | 1145.57323 | 573.29025 | 11 |
| 8 | 765.37780 | 383.19254 | V | 1046.50481 | 523.75604 | 10 |
| 9 | 864.44622 | 432.72675 | V | 947.43639 | 474.22183 | 9 |
| 10 | 1001.50513 | 501.25620 | H | 848.36797 | 424.68762 | 8 |
| 11 | 1152.49906 | 576.75317 | C-hyper | 711.30906 | 356.15817 | 7 |
| 12 | 1239.53109 | 620.26918 | S | 560.31512 | 280.66120 | 6 |
| 13 | 1310.56821 | 655.78774 | A | 473.28309 | 237.14518 | 5 |
| 14 | 1367.58968 | 684.29848 | G | 402.24597 | 201.62662 | 4 |
| 15 | 1480.67375 | 740.84051 | I | 345.22450 | 173.11589 | 3 |
| 16 | 1537.69522 | 769.35125 | G | 232.14043 | 116.57385 | 2 |
| 17 | | | R | 175.11896 | 88.06312 | 1 |

**Fragment Spectrum**

Extracted from: D:\wu000024\20161117_Fin05_3reps\Set3\20161120_OR12_A6_WW_Fin05_13_4.raw #5395 RT: 17.34
FTMS, HCD@27.00, z=+2, Mono m/z=856.40961 Da, MH+=1711.81194 Da, Match Tol.=0.05 Da

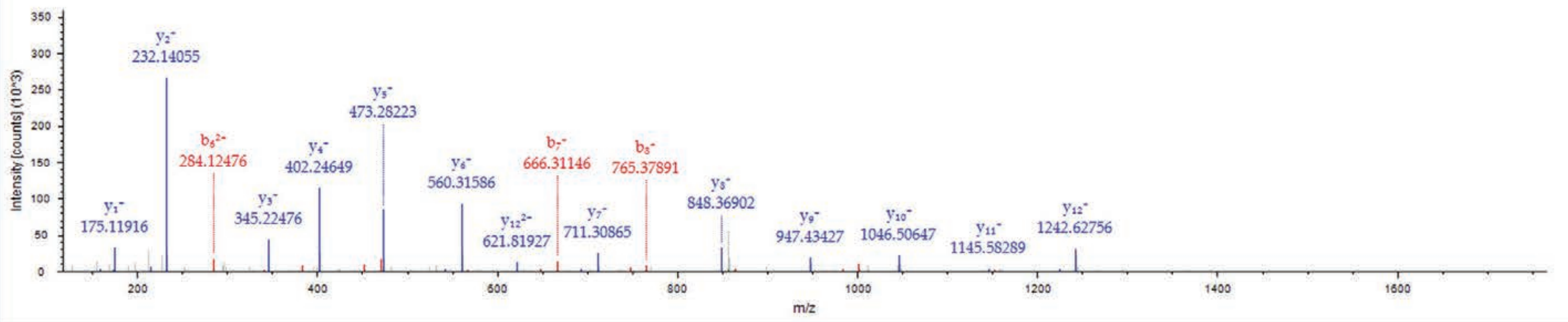

| Uniprot Accession | Description | Gene name | PTP type | Sequence | Hyperoxidised cysteine | Total MS/MS count |
|---|---|---|---|---|---|---|
| C7U118 | Protein-tyrosine phosphatase SHP-1 (Fragment) | ptpn6 | Non-receptor | SSAPIVIHcSAGIGR | C9(hyper) | 82 |

Peptide Summary

Sequence: SSAPIVIHCSAGIGR, C9-hyperoxidation (47.98474 Da)
Charge: +2, Monoisotopic m/z: 758.38428 Da (+1.4 mmu/+1.85 ppm), MH+: 1515.76128 Da, RT: 32.17 min,
Identified with: Mascot (v1.30); IonScore:125, Exp Value:4.1E-011, Ions matched by search engine: 11/130
Fragment match tolerance used for search: 0.05 Da

Fragment Matches

Value Type: Theo. Mass [Da]

Ion Series | Neutral Losses | Precursor Ions

| #1 | b+ | b2+ | Seq. | y+ | y2+ | #2 |
|---|---|---|---|---|---|---|
| 1 | 88.03931 | 44.52329 | S | | | 15 |
| 2 | 175.07134 | 88.03931 | S | 1428.72645 | 714.86686 | 14 |
| 3 | 246.10846 | 123.55787 | A | 1341.69442 | 671.35085 | 13 |
| 4 | 343.16123 | 172.08425 | P | 1270.65730 | 635.83229 | 12 |
| 5 | 456.24530 | 228.62629 | I | 1173.60453 | 587.30590 | 11 |
| 6 | 555.31372 | 278.16050 | V | 1060.52046 | 530.76387 | 10 |
| 7 | 668.39779 | 334.70253 | I | 961.45204 | 481.22966 | 9 |
| 8 | 805.45670 | 403.23199 | H | 848.36797 | 424.68762 | 8 |
| 9 | 956.45063 | 478.72895 | C-hyper | 711.30906 | 356.15817 | 7 |
| 10 | 1043.48266 | 522.24497 | S | 560.31512 | 280.66120 | 6 |
| 11 | 1114.51978 | 557.76353 | A | 473.28309 | 237.14518 | 5 |
| 12 | 1171.54125 | 586.27426 | G | 402.24597 | 201.62662 | 4 |
| 13 | 1284.62532 | 642.81630 | I | 345.22450 | 173.11589 | 3 |
| 14 | 1341.64679 | 671.32703 | G | 232.14043 | 116.57385 | 2 |
| 15 | | | R | 175.11896 | 88.06312 | 1 |

Fragment Spectrum

Extracted from: D:\wu000024\20161117_Fin05_3reps\Set1\20161117_OR12_A6_WW_Fin05_1_1.raw #10765 RT: 32.17
FTMS, HCD@27.00, z=+2, Mono m/z=758.38428 Da, MH+=1515.76128 Da, Match Tol.=0.05 Da

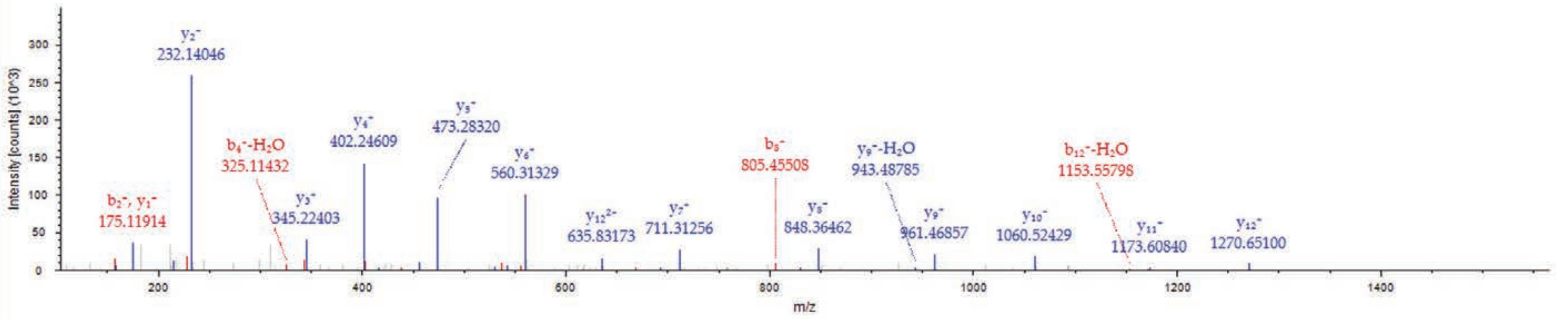

| Uniprot Accession | Description | Gene name | PTP type | Sequence | Hyperoxidised cysteine | Total MS/MS count |
|---|---|---|---|---|---|---|
| X1WFV4 | Uncharacterized protein (Fragment) | ptpn9a | Non-receptor | GHPLGPPMVVHcSAGIGR | C12(hyper) | 170 |

Peptide Summary

Sequence: GHPLGPPMVVHCSAGIGR, M8-Oxidation (15.99492 Da), C12-hyperoxidation (47.98474 Da)
Charge: +2, Monoisotopic m/z: 924.94702 Da (+1.16 mmu/+1.25 ppm), MH+: 1848.88677 Da, RT: 17.30 min,
Identified with: Mascot (v1.30); IonScore:123, Exp Value:7.6E-011, Ions matched by search engine: 12/136
Fragment match tolerance used for search: 0.05 Da

Fragment Matches

Value Type: Theo. Mass [Da]

Ion Series | Neutral Losses | Precursor Ions

| #1 | b+ | b2+ | Seq. | y+ | y2+ | #2 |
|---|---|---|---|---|---|---|
| 1 | 58.02875 | 29.51801 | G | | | 18 |
| 2 | 195.08766 | 98.04747 | H | 1791.86298 | 896.43513 | 17 |
| 3 | 292.14043 | 146.57385 | P | 1654.80407 | 827.90567 | 16 |
| 4 | 405.22450 | 203.11589 | L | 1557.75130 | 779.37929 | 15 |
| 5 | 462.24597 | 231.62662 | G | 1444.66723 | 722.83725 | 14 |
| 6 | 559.29874 | 280.15301 | P | 1387.64576 | 694.32652 | 13 |
| 7 | 656.35151 | 328.67939 | P | 1290.59299 | 645.80013 | 12 |
| 8 | 803.38692 | 402.19710 | M-Oxidation | 1193.54022 | 597.27375 | 11 |
| 9 | 902.45534 | 451.73131 | V | 1046.50481 | 523.75604 | 10 |
| 10 | 1001.52376 | 501.26552 | V | 947.43639 | 474.22183 | 9 |
| 11 | 1138.58267 | 569.79497 | H | 848.36797 | 424.68762 | 8 |
| 12 | 1289.57661 | 645.29194 | C-hyper | 711.30906 | 356.15817 | 7 |
| 13 | 1376.60864 | 688.80796 | S | 560.31512 | 280.66120 | 6 |
| 14 | 1447.64576 | 724.32652 | A | 473.28309 | 237.14518 | 5 |
| 15 | 1504.66723 | 752.83725 | G | 402.24597 | 201.62662 | 4 |
| 16 | 1617.75130 | 809.37929 | I | 345.22450 | 173.11589 | 3 |
| 17 | 1674.77277 | 837.89002 | G | 232.14043 | 116.57385 | 2 |
| 18 | | | R | 175.11896 | 88.06312 | 1 |

Fragment Spectrum

Extracted from: D:\wu000024\20161117_Fin05_3reps\Set3\20161120_OR12_A6_WW_Fin05_13_1.raw #5357 RT: 17.30
FTMS, HCD@27.00, z=+2, Mono m/z=924.94702 Da, MH+=1848.88677 Da, Match Tol.=0.05 Da

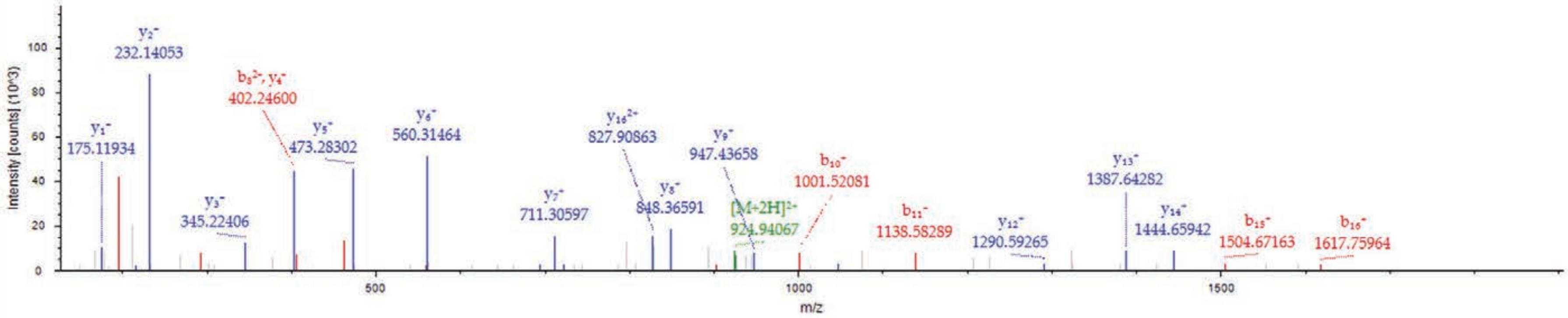

| Uniprot Accession | Description | Gene name | PTP type | Sequence | Hyperoxidised cysteine | Total MS/MS count |
|---|---|---|---|---|---|---|
| F1QTI4 | Uncharacterized protein | ptpn9b | Non-receptor | TLYPDWTGPPGGPPVVVHcSAGIGR | C19(hyper) | 86 |

Peptide Summary

Sequence: TLYPDWTGPPGGPPVVVHCSAGIGR, C19-hyperoxidation (47.98474 Da)
Charge: +2, Monoisotopic m/z: 1291.13000 Da (+1.11 mmu/+0.86 ppm), MH+: 2581.25273 Da, RT: 23.73 min,
Identified with: Mascot (v1.30); IonScore:154, Exp Value:5.0E-014, Ions matched by search engine: 16/230
Fragment match tolerance used for search: 0.05 Da

Fragment Matches

Value Type: Theo. Mass [Da]

Ion Series | Neutral Losses | Precursor Ions

| #1 | b+ | b2+ | Seq. | y+ | y2+ | #2 |
|---|---|---|---|---|---|---|
| 1 | 102.05496 | 51.53112 | T | | | 25 |
| 2 | 215.13903 | 108.07315 | L | 2480.20283 | 1240.60505 | 24 |
| 3 | 378.20235 | 189.60481 | Y | 2367.11876 | 1184.06302 | 23 |
| 4 | 475.25512 | 238.13120 | P | 2204.05544 | 1102.53136 | 22 |
| 5 | 590.28207 | 295.64467 | D | 2107.00267 | 1054.00497 | 21 |
| 6 | 776.36139 | 388.68433 | W | 1991.97572 | 996.49150 | 20 |
| 7 | 877.40907 | 439.20817 | T | 1805.89640 | 903.45184 | 19 |
| 8 | 934.43054 | 467.71891 | G | 1704.84872 | 852.92800 | 18 |
| 9 | 1031.48331 | 516.24529 | P | 1647.82725 | 824.41726 | 17 |
| 10 | 1128.53608 | 564.77168 | P | 1550.77448 | 775.89088 | 16 |
| 11 | 1185.55755 | 593.28241 | G | 1453.72171 | 727.36449 | 15 |
| 12 | 1242.57902 | 621.79315 | G | 1396.70024 | 698.85376 | 14 |
| 13 | 1339.63179 | 670.31953 | P | 1339.67877 | 670.34302 | 13 |
| 14 | 1436.68456 | 718.84592 | P | 1242.62600 | 621.81664 | 12 |
| 15 | 1535.75298 | 768.38013 | V | 1145.57323 | 573.29025 | 11 |
| 16 | 1634.82140 | 817.91434 | V | 1046.50481 | 523.75604 | 10 |
| 17 | 1733.88982 | 867.44855 | V | 947.43639 | 474.22183 | 9 |
| 18 | 1870.94873 | 935.97800 | H | 848.36797 | 424.68762 | 8 |
| 19 | 2021.94266 | 1011.47497 | C-hyper | 711.30906 | 356.15817 | 7 |
| 20 | 2108.97469 | 1054.99098 | S | 560.31512 | 280.66120 | 6 |
| 21 | 2180.01181 | 1090.50954 | A | 473.28309 | 237.14518 | 5 |
| 22 | 2237.03328 | 1119.02028 | G | 402.24597 | 201.62662 | 4 |

Fragment Spectrum

Extracted from: D:\wu000024\20161117_Fin05_3reps\Set2\20161118_OR12_A6_WW_Fin05_7_2.raw #7496 RT: 23.73
FTMS, HCD@27.00, z=+2, Mono m/z=1291.13000 Da, MH+=2581.25273 Da, Match Tol.=0.05 Da

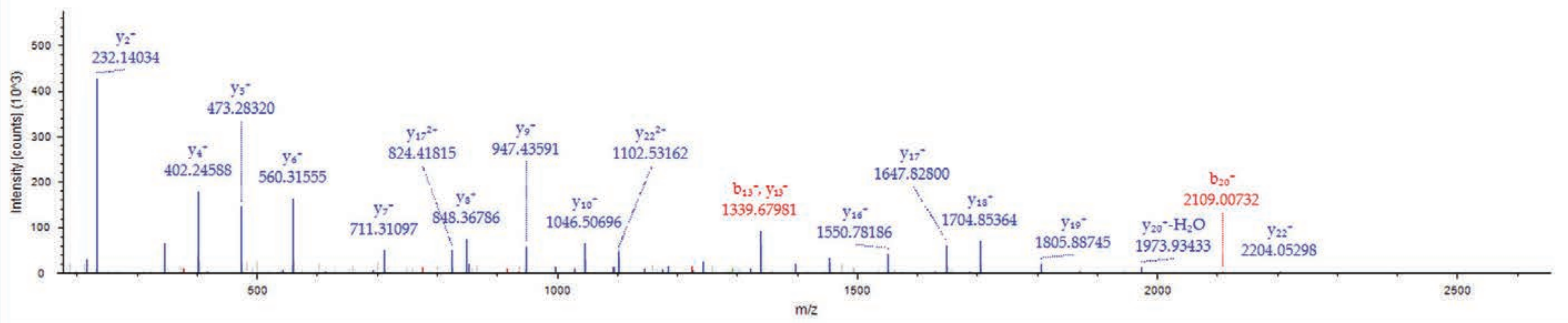

**Fig. S3. Zebrafish *ptpra* knock-out embryos (*ptpra*$^{-/-}$) lack RPTPα protein.** Adult zebrafish heterozygous for *ptpra* (*ptpra*$^{+/-}$) were in-crossed to generate *ptpra* knock-out embryos (*ptpra*$^{-/-}$). At 4 dpf, individual embryos were cut in half: one half lysed for protein, and the other half lysed for DNA extraction. Following genotyping, protein lysates of *ptpra*$^{+/+}$, *ptpra*$^{+/-}$, and *ptpra*$^{-/-}$ embryos were pooled, and 5 embryo equivalents per condition were run on an SDS-PAGE gel, immunoblotted and probed with an Rptpα-specific antibody and with tubulin to monitor equal loading. Control lane represents wild-type TL zebrafish embryos at 3 dpf.

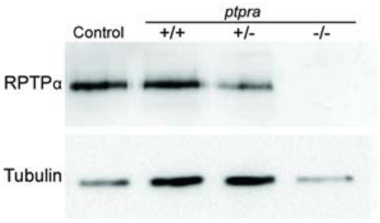

**Table S1. PTP motif peptides detected.** Conserved catalytic site sequence in blue. Position of hyper-oxidized cysteine refers to residue number within a properly cleaved tryptic peptide, without missed cleavage. Total MS/MS count is the sum of all spectra obtained from each biological replicate of PV-treated sample. Peptides oxidized in the second fin clip are highlighted in grey.

| Uniprot Accession | Description | Gene name | PTP type | Sequence | Hyperoxidized cysteine | Total MS/MS count |
|---|---|---|---|---|---|---|
| B3DJL5_site1 (C7U129) | Ptpra protein | ptpra | Receptor | QQQQSGNHPIT**VHcSAGAGR** | C14(hyper) | 76 |
| B3DJL5_site2 (C7U129) | Ptpra protein | ptpra | Receptor | NcNPQYAGPIV**VHcSAGVGR** | C14(hyper) | 33 |
| A0A0R4ITJ9 (C7U135) | Uncharacterized protein | ptprb | Receptor | TNSPGISV**VHcSAGVGR** | C11(hyper) | 51 |
| C7U126 | CD45 antigen (Fragment) | ptprc | Receptor | NFFSGPIV**VHcSAGVGR** | C11(hyper) | 93 |
| E7EZG7_site1 | Receptor-type tyrosine-protein phosphatase | ptprea | Receptor | QVNPSYAGPIV**VHcSAGVGR** | C14(hyper) | 35 |
| E7EZG7_site2 | Receptor-type tyrosine-protein phosphatase | ptprea | Receptor | QQQQSGNHPII**VHcSAGAGR** | C14(hyper) | 119 |
| Q90YJ5_site1 | Receptor protein-tyrosine phosphatase LAR (Fragment) | ptprfa | Receptor | AcNPPDAGPMV**VHcSAGVGR** | C14(hyper) | 100 |
| Q90YJ5_site2 | Receptor protein-tyrosine phosphatase LAR (Fragment) | ptprfa | Receptor | EQFGQDGPIT**VHcSAGVGR** | C13(hyper) | 72 |
| F1Q4V8 | Uncharacterized protein (Fragment) | ptprga | Receptor | SSAAQTPDMGPML**VHcSAGVGR** | C16(hyper) | 42 |
| E7F9R2 | Uncharacterized protein | ptprh | Receptor | QHIESHFSTGPTV**VHcSAGVGR** | C16(hyper) | 159 |
| B6HY54 | Protein-tyrosine phosphatase | ptprja | Receptor | HSPTL**VHcSAGVGR** | C8(hyper) | 52 |
| X1WCM2 | Uncharacterized protein (Fragment) | ptprjb.1 | Receptor | HSPTV**VHcSAGVGR** | C8(hyper) | 21 |
| X1WF45 | Uncharacterized protein (Fragment) | ptprjb.2 | Receptor | EHMNQYScTSPTV**VHcSAGVGR** | C16(hyper) | 6 |
| X1WD65 | Uncharacterized protein | ptprk | Receptor | MSNPPTAGPIV**VHcSAGAGR** | C14(hyper) | 90 |
| A0A0R4IA95 | Uncharacterized protein (Fragment) | ptpro | Receptor | GPIV**VHcSAGVGR** | C7(hyper) | 52 |
| Q90YJ4_site1 | Receptor protein-tyrosine phosphatase sigma (Fragment) | ptprsa | Receptor | EQFGQDGPIS**VHcSAGVGR** | C13(hyper) | 21 |
| Q90YJ4_site2 | Receptor protein-tyrosine phosphatase sigma (Fragment) | ptprsa | Receptor | TcNPPDAGPII**AHcSAGVGR** | C14(hyper) | 45 |
| A0A0R4ITF5 (C7U125) | Uncharacterized protein | ptprub | Receptor | TSTPLDAGPVV**VHcSVGAGR** | C14(hyper) | 13 |
| X1WET2 | Uncharacterized protein (Fragment) | ptprz1a | Receptor | AQTDGMGPMV**VHcSAGVGR** | C13(hyper) | 16 |
| B8A5J4 | Uncharacterized protein | ptprz1b | Receptor | TAEMGPVV**VHcSAGVGR** | C11(hyper) | 104 |
| C7U142 | Protein-tyrosine phosphatase 1B (Fragment) | ptpn1 | Non-receptor | ESGcLSPELGPVV**VHcSAGIGR** | C16(hyper) | 142 |
| C7U151 | Protein-tyrosine phosphatase SHP-2a (Fragment) | ptpn11a | Non-receptor | QEGITGAGPIV**VHcSAGIGR** | C14(hyper) | 112 |
| C7U120 | Protein-tyrosine phosphatase SHP-2b (Fragment) | ptpn11b | Non-receptor | TQSAIPESGPIV**VHcSAGIGR** | C15(hyper) | 59 |
| Q6PEI4 | Protein tyrosine phosphatase, non-receptor type 12 | ptpn12 | Non-receptor | DTTPIc**VHcSAGcGR** | C13(hyper) | 71 |
| C7U124 | Protein-tyrosine phosphatase BAS (Fragment) | ptpn13 | Non-receptor | HIHLSGPII**THcSAGIGR** | C12(hyper) | 64 |
| F1QIX3 | Uncharacterized protein | ptpn20 | Non-receptor | GPIT**VHcSAGIGR** | C7(hyper) | 38 |
| F1Q6T9 | Uncharacterized protein | ptpn21 | Non-receptor | NTNLPVL**VHcSAGVGR** | C10(hyper) | 29 |
| C7U128 | Protein-tyrosine phosphatase LyPTP (Fragment) | ptpn22 | Non-receptor | IYQDNEEIPIc**IHcSAGcGR** | C18(hyper) | 13 |
| F1R1D1 | Uncharacterized protein | ptpn23a | Non-receptor | VScTHFQ**cSAGA**FAYLR | C8(hyper) | 138 |
| F1QXQ5 (F1QGX6) | Tyrosine-protein phosphatase non-receptor type | ptpn2a | Non-receptor | ESGSLGPENGPVV**VHcSAGIGR** | C16(hyper) | 86 |
| Q6NY86 | Tyrosine-protein phosphatase non-receptor type | ptpn2b | Non-receptor | ESGSLGMEQGPAV**VHcSAGIGR** | C16(hyper) | 159 |
| F1QLN8 | Uncharacterized protein | ptpn3 | Non-receptor | QESVPLM**VHcSAGIGR** | C10(hyper) | 97 |
| C7U127 | Protein-tyrosine phosphatase MEG1a (Fragment) | ptpn4a | Non-receptor | DEPVV**VHcSAGIGR** | C8(hyper) | 19 |
| C7U113 | Protein-tyrosine phosphatase MEG1a (Fragment) | ptpn4b | Non-receptor | ADGPEPVV**VHcSAGIGR** | C11(hyper) | 156 |
| C7U118 | Protein-tyrosine phosphatase SHP-1 (Fragment) | ptpn6 | Non-receptor | SSAPIV**IHcSAGIGR** | C9(hyper) | 82 |
| X1WFV4 | Uncharacterized protein (Fragment) | ptpn9a | Non-receptor | GHPLGPPMV**VHcSAGIGR** | C12(hyper) | 170 |
| F1QTI4 | Uncharacterized protein | ptpn9b | Non-receptor | TLYPDWTGPPGGPPVV**VHcSAGIGR** | C19(hyper) | 86 |

**Table S2. PTP motif peptide oxidation in response to caudal fin amputation.** Mean spectra count and standard error obtained from 3 biological replicates. Peptides oxidized in second fin clip highlighted in grey. Student *t*-test statistics for difference between first and second fin clip reported as exact two-tailed *p*-value.

| | | | | First fin clip | | | | | | Second fin clip | | | | | | | |
|---|---|---|---|---|---|---|---|---|---|---|---|---|---|---|---|---|---|
| Uniprot Accession | Gene name | PTP type | Total MS/MS count | Mean spectra count PV | SEM PV | Mean spectra count NEM | SEM NEM | Mean spectra count Control | SEM Control | Mean spectra count PV | SEM PV | Mean spectra count NEM | SEM NEM | Mean spectra count Control | SEM Control | T.test (PV 1st clip: PV 2nd clip) | T.test (NEM 2nd clip : NEM 1st clip) |
| B3DJL5_site1 (C7U129) | ptpra | Receptor | 76 | 13,0 | 2,1 | 0,7 | 0,3 | 0,0 | 0,0 | 11,3 | 1,2 | 0,3 | 0,3 | 0,0 | 0,0 | 0,62 | 0,21 |
| B3DJL5_site2 (C7U129) | ptpra | Receptor | 33 | 6,7 | 0,9 | 0,7 | 0,7 | 0,0 | 0,0 | 3,3 | 0,9 | 0,3 | 0,3 | 0,0 | 0,0 | 0,20 | 0,42 |
| A0A0R4ITJ9 (C7U135) | ptprb | Receptor | 51 | 9,0 | 0,6 | 0,0 | 0,0 | 0,0 | 0,0 | 8,0 | 1,7 | 0,0 | 0,0 | 0,0 | 0,0 | 0,48 | Not detected |
| C7U126 | ptprc | Receptor | 93 | 18,0 | 1,7 | 0,3 | 0,3 | 0,0 | 0,0 | 12,3 | 0,9 | 0,3 | 0,3 | 0,0 | 0,0 | 0,14 | Not detected |
| E7EZG7_site1 | ptprea | Receptor | 35 | 6,3 | 0,9 | 0,7 | 0,7 | 0,0 | 0,0 | 4,0 | 1,2 | 0,7 | 0,3 | 0,0 | 0,0 | 0,12 | 1,00 |
| E7EZG7_site2 | ptprea | Receptor | 119 | 18,0 | 1,5 | 0,3 | 0,3 | 0,0 | 0,0 | 15,3 | 0,9 | 6,0 | 1,2 | 0,0 | 0,0 | 0,37 | 0,04 |
| Q90YJ5_site1 | ptprfa | Receptor | 100 | 22,0 | 2,3 | 0,0 | 0,0 | 0,0 | 0,0 | 11,3 | 0,7 | 0,0 | 0,0 | 0,0 | 0,0 | 0,07 | Not detected |
| Q90YJ5_site2 | ptprfa | Receptor | 72 | 12,3 | 1,8 | 0,3 | 0,3 | 0,0 | 0,0 | 11,0 | 1,5 | 0,3 | 0,3 | 0,0 | 0,0 | 0,63 | Not detected |
| F1Q4V8 | ptprga | Receptor | 42 | 8,7 | 1,5 | 0,0 | 0,0 | 0,0 | 0,0 | 5,3 | 0,3 | 0,0 | 0,0 | 0,0 | 0,0 | 0,20 | Not detected |
| E7F9R2 | ptprh | Receptor | 159 | 22,0 | 1,5 | 0,0 | 0,0 | 0,0 | 0,0 | 19,3 | 2,0 | 11,7 | 1,5 | 0,0 | 0,0 | 0,38 | 0,02 |
| B6HY54 | ptprja | Receptor | 52 | 8,0 | 0,6 | 1,0 | 0,0 | 0,0 | 0,0 | 8,3 | 0,9 | 0,0 | 0,0 | 0,0 | 0,0 | 0,83 | Not detected |
| X1WCM2 | ptprjb.1 | Receptor | 21 | 4,0 | 1,2 | 0,0 | 0,0 | 0,0 | 0,0 | 3,0 | 1,0 | 0,0 | 0,0 | 0,0 | 0,0 | 0,68 | Not detected |
| X1WF45 | ptprjb.2 | Receptor | 6 | 1,0 | 0,0 | 0,0 | 0,0 | 0,0 | 0,0 | 0,7 | 0,3 | 0,3 | 0,3 | 0,0 | 0,0 | 0,42 | 0,42 |
| X1WD65 | ptprk | Receptor | 90 | 16,7 | 0,9 | 0,0 | 0,0 | 0,0 | 0,0 | 13,0 | 0,6 | 0,3 | 0,3 | 0,0 | 0,0 | 0,05 | 0,42 |
| A0A0R4IA95 | ptpro | Receptor | 52 | 8,3 | 0,9 | 0,3 | 0,3 | 0,0 | 0,0 | 8,3 | 1,2 | 0,3 | 0,3 | 0,0 | 0,0 | 1,00 | Not detected |
| Q90YJ4_site1 | ptprsa | Receptor | 21 | 4,0 | 0,6 | 0,0 | 0,0 | 0,0 | 0,0 | 2,7 | 0,3 | 0,3 | 0,3 | 0,0 | 0,0 | 0,06 | 0,42 |
| Q90YJ4_site2 | ptprsa | Receptor | 45 | 8,0 | 1,0 | 0,0 | 0,0 | 0,0 | 0,0 | 7,0 | 1,7 | 0,0 | 0,0 | 0,0 | 0,0 | 0,42 | Not detected |
| A0A0R4ITF5 (C7U125) | ptprub | Receptor | 13 | 2,3 | 0,3 | 0,0 | 0,0 | 0,0 | 0,0 | 2,0 | 0,0 | 0,0 | 0,0 | 0,0 | 0,0 | 0,42 | Not detected |
| X1WET2 | ptprz1a | Receptor | 16 | 3,7 | 0,3 | 0,0 | 0,0 | 0,0 | 0,0 | 1,3 | 0,3 | 0,3 | 0,3 | 0,0 | 0,0 | 0,07 | 0,42 |
| B8A5J4 | ptprz1b | Receptor | 104 | 21,7 | 2,4 | 0,0 | 0,0 | 0,0 | 0,0 | 12,7 | 1,5 | 0,3 | 0,3 | 0,0 | 0,0 | 0,12 | 0,42 |
| C7U142 | ptpn1 | Non-receptor | 142 | 22,0 | 2,1 | 0,7 | 0,7 | 0,3 | 0,3 | 20,7 | 1,5 | 3,7 | 0,7 | 0,0 | 0,0 | 0,73 | 0,04 |
| C7U151 | ptpn11a | Non-receptor | 112 | 14,7 | 1,5 | 0,7 | 0,3 | 0,3 | 0,3 | 11,3 | 0,9 | 10,3 | 0,9 | 0,0 | 0,0 | 0,27 | 0,00 |
| C7U120 | ptpn11b | Non-receptor | 59 | 7,3 | 0,3 | 0,0 | 0,0 | 0,0 | 0,0 | 5,0 | 0,6 | 7,3 | 0,7 | 0,0 | 0,0 | 0,07 | 0,01 |
| Q6PEI4 | ptpn12 | Non-receptor | 71 | 13,0 | 1,2 | 0,0 | 0,0 | 0,0 | 0,0 | 10,7 | 1,5 | 0,0 | 0,0 | 0,0 | 0,0 | 0,46 | Not detected |
| C7U124 | ptpn13 | Non-receptor | 64 | 13,3 | 2,0 | 1,0 | 0,6 | 0,0 | 0,0 | 6,7 | 0,7 | 0,3 | 0,3 | 0,0 | 0,0 | 0,12 | 0,53 |
| F1QIX3 | ptpn20 | Non-receptor | 38 | 7,0 | 1,0 | 0,0 | 0,0 | 0,0 | 0,0 | 5,7 | 1,2 | 0,0 | 0,0 | 0,0 | 0,0 | 0,06 | Not detected |
| F1Q6T9 | ptpn21 | Non-receptor | 29 | 4,0 | 0,6 | 1,0 | 0,6 | 0,0 | 0,0 | 4,3 | 1,2 | 0,3 | 0,3 | 0,0 | 0,0 | 0,67 | 0,42 |
| C7U128 | ptpn22 | Non-receptor | 13 | 3,0 | 0,0 | 0,0 | 0,0 | 0,0 | 0,0 | 1,0 | 0,6 | 0,3 | 0,3 | 0,0 | 0,0 | 0,07 | 0,42 |
| F1R1D1 | ptpn23a | Non-receptor | 138 | 24,7 | 2,0 | 0,0 | 0,0 | 0,0 | 0,0 | 21,0 | 1,5 | 0,3 | 0,3 | 0,0 | 0,0 | 0,39 | 0,42 |
| F1QXQ5 (F1QGX6) | ptpn2a | Non-receptor | 86 | 15,7 | 0,7 | 0,3 | 0,3 | 0,0 | 0,0 | 9,7 | 0,9 | 3,0 | 0,6 | 0,0 | 0,0 | 0,06 | 0,04 |
| Q6NY86 | ptpn2b | Non-receptor | 159 | 32,7 | 2,8 | 0,3 | 0,3 | 0,0 | 0,0 | 19,7 | 3,3 | 0,0 | 0,0 | 0,3 | 0,3 | 0,16 | 0,42 |
| F1QLN8 | ptpn3 | Non-receptor | 97 | 17,0 | 1,5 | 0,7 | 0,3 | 0,0 | 0,0 | 14,3 | 2,3 | 0,3 | 0,3 | 0,0 | 0,0 | 0,37 | 0,42 |
| C7U127 | ptpn4a | Non-receptor | 19 | 3,3 | 0,3 | 0,0 | 0,0 | 0,0 | 0,0 | 3,0 | 1,2 | 0,0 | 0,0 | 0,0 | 0,0 | 0,81 | Not detected |
| C7U113 | ptpn4b | Non-receptor | 156 | 27,7 | 1,5 | 0,0 | 0,0 | 0,0 | 0,0 | 22,3 | 0,7 | 5,7 | 0,3 | 0,0 | 0,0 | 0,09 | 0,00 |
| C7U118 | ptpn6 | Non-receptor | 82 | 15,7 | 1,5 | 0,7 | 0,7 | 0,3 | 0,3 | 10,3 | 0,3 | 0,0 | 0,0 | 0,3 | 0,3 | 0,09 | 0,42 |
| X1WFV4 | ptpn9a | Non-receptor | 170 | 28,7 | 1,9 | 0,3 | 0,3 | 0,0 | 0,0 | 22,3 | 1,5 | 5,3 | 0,9 | 0,0 | 0,0 | 0,18 | 0,01 |
| F1QTI4 | ptpn9b | Non-receptor | 86 | 16,3 | 0,9 | 0,7 | 0,3 | 0,0 | 0,0 | 11,3 | 1,5 | 0,3 | 0,3 | 0,0 | 0,0 | 0,13 | 0,42 |
